# Differential host gene responses from infection with neurovirulent and partially-neurovirulent strains of Venezuelan equine encephalitis virus

**DOI:** 10.1186/s12879-017-2355-3

**Published:** 2017-04-26

**Authors:** Paridhi Gupta, Anuj Sharma, Jing Han, Amy Yang, Manish Bhomia, Barbara Knollmann-Ritschel, Raj K Puri, Radha K Maheshwari

**Affiliations:** 1 Department of Pathology, Uniformed Services University of the Health Sciences, 4301 Jones Bridge Road, Bethesda, MD 20814 USA; 2 Division of Cellular and Gene Therapies, Center for Biologics Evaluation and Research, Food and Drug Administration, Bethesda, MD USA

**Keywords:** Venezuelan equine encephalitis virus, Host responses, Whole genome microarray, Neurovirulence, V3000, V3034

## Abstract

**Background:**

Venezuelan equine encephalitis virus (VEEV) is an alphavirus in the family *Togaviridae*. VEEV causes a bi-phasic illness in mice where primary replication in lymphoid organs is followed by entry into the central nervous system (CNS). The CNS phase of infection is marked by encephalitis and large scale neuronal death ultimately resulting in death. Molecular determinants of VEEV neurovirulence are not well understood. In this study, host gene expression response to highly neurovirulent VEEV (V3000 strain) infection was compared with that of a partially neurovirulent VEEV (V3034 strain) to identify host factors associated with VEEV neurovirulence.

**Methods:**

Whole genome microarrays were performed to identify the significantly modulated genes. Microarray observations were classified into three categories *i.e.*, genes that were similarly modulated against both V3000 and V3034 infections, and genes that were uniquely modulated in infection with V3034 or V3000. Histologic sections of spleen and brain were evaluated by hematoxylin and eosin stains from all the mice.

**Results:**

V3000 infection induced a greater degree of pathology in both the spleen and brain tissue of infected mice compared to V3034 infection. Genes commonly modulated in the spleens after V3000 or V3034 infection were associated with innate immune responses, inflammation and antigen presentation, however, V3000 induced a gene response profile that suggests a stronger inflammatory and apoptotic response compared to V3034. In the brain, both the strains of VEEV induced an innate immune response reflected by an upregulation of the genes involved in antigen presentation, interferon response, and inflammation. Similar to the spleen, V3000 was found to induce a stronger inflammatory response than V3034 in terms of induction of pro-inflammatory genes and associated pathways. *Ccl2*, *Ccl5*, *Ccl6*, and *Ly6* were uniquely upregulated in V3000 infected mouse brains and correlated with the extensive inflammation observed in the brain.

**Conclusion:**

The common gene profile identified from V3000 and V3034 exposure can help in understanding a generalized host response to VEEV infection. Inflammatory genes that were uniquely identified in mouse brains with V3000 infection will help in better understanding the lethal neurovirulence of VEEV. Future studies are needed to explore the roles played by the genes identified in VEEV induced encephalitis.

**Electronic supplementary material:**

The online version of this article (doi:10.1186/s12879-017-2355-3) contains supplementary material, which is available to authorized users.

## Background

Venezuelan equine encephalitis virus (VEEV) is a highly infectious emerging pathogen of bioterrorism interest. VEEV is an arthropod borne virus in the family *Togaviridae*. VEEV causes a characteristic bi-phasic illness where the primary replication in lymphoid tissues is followed by viral replication in central nervous system (CNS) (Johnston and Peter, 1996). CNS infection is dependent on the ability of VEEV to replicate to a high titer in the circulation, which helps the escape of the virus from the blood circulation into the fenestrated neuroepithelium of the olfactory tract, and ultimately into the olfactory lobe of the brain [1–3]. Once in brain, VEEV replicates in the neurons and glia and spreads centripetally from olfactory lobe to the mid brain and cerebellum [2]. Mortality in humans is low (< 1%), but in equines VEEV can cause mortality as high as 83% [4]. Severe morbidity and mortality is associated with viral replication in CNS, as well as possible permanent long term neurological deficits [5]. Within the CNS, excessive inflammation has been suggested to contribute to increased neuronal and glial cell death in addition to direct neuronal cell death from VEEV infection [6, 7]. VEEV infection has been shown to induce inflammatory and innate immune response genes in the brain [8–10]. However, the molecular mechanisms underlying the neurovirulence of VEEV are not fully understood.

The host-virus interactions are generally reflected as specific changes in the gene-expression pattern and signal transduction pathways in the host [11]. Earlier, Grieder et al [3] have developed various site directed VEEV mutants that showed varying degree of neurovirulence [3]. These mutants can be used to study the differential pathogenesis of VEEV strains and may help in better understanding the neuropathology of VEEV. Therefore, in this study we evaluated the gene expression modulation in response to infection with a neurovirulent, V3000, and a partially neurovirulent, V3034, strains of VEEV. V3000 is a full length cDNA clone of the Trinidad Donkey (TrD) strain of VEEV. It is a neuroinvasive strain that is as neurovirulent and infectious as the wild type virus (TrD strain) by peripheral, intracranial or aerosol inoculation [12]. V3034 is neuroinvasive but a partially lethal mutant of TrD strain with a single substitution (Threonine for Alanine) at El codon 272, resulting in 11% mortality in mice infected either by peripheral or intracranial infection or 80% mortality via aerosol exposure [3]. In this study, we show that VEEV infection induced activation of germinal centers in the spleen. The severity of tissue pathology was markedly pronounced in V3000 infected mice as compared to V3034 infected mice. In the brain, inflammation was induced by both the viral strains and, similar to spleen, V3000 infection demonstrated increased and more widespread inflammation. Several genes were similarly modulated during infection with both the strains of VEEV suggesting that there may be a VEEV typical host response irrespective of the level of the virulence of the infecting strain. Several genes were differentially or uniquely modulated during infection with V3000 and V3034 infection suggesting induction of a viral strain-specific response from the host.

## Results

### Neurovirulent and partially neurovirulent strains of VEEV induce markedly different pathological response in the spleen and brain.

#### VEEV infection in spleen

**Fig. 1:**
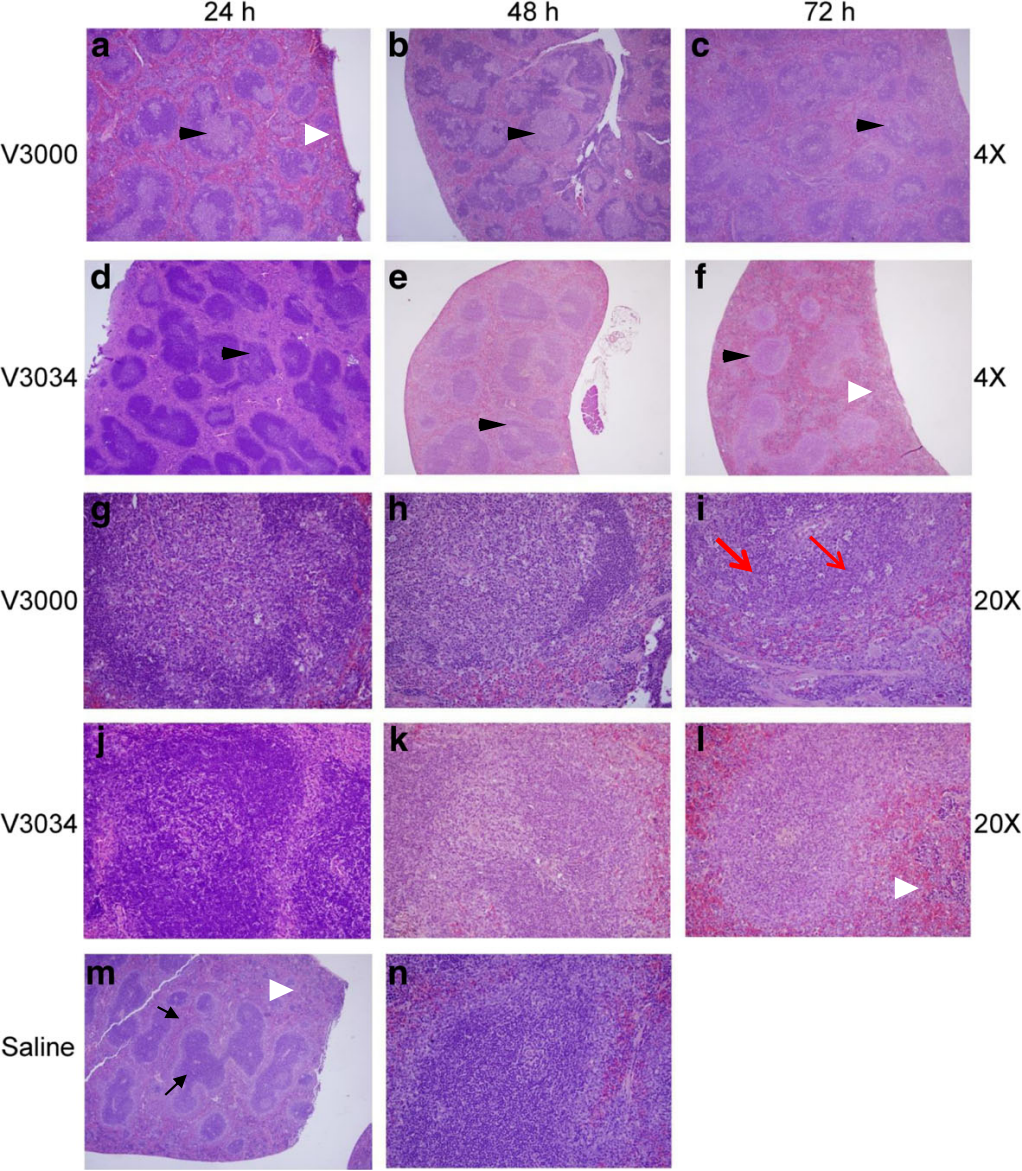
Histology of the spleen following infection with V3000 or V3034 strains of VEEV. Spleen sections were analyzed by H&E staining (**a**) V3000 at 24 h (4X), (**b**) V3000 at 48 h (4X), (**c**) V3000 at 72 h (4X), (**d**) V3034 at 24 h (4X), (**e**) V3034 at 48 h (4X), (**f**) V3034 at 72 h (4X), (**g**) V3000 at 24 h (20X), (**h**) V3000 at 48 h (20X), (**i**) V3000 at 72 h (20X), (**j**) V3034 at 24 h (20X), (**k**) V3034 at 48 h (20X), (**l**) V3034 at 72 h (20X), (**m**) Saline (4X) and (**n**) Saline (20X). Spleens from saline-injected control animals showed normal red and white pulp area (thin arrows). Spleens from V3000 or V3034 infected mice exhibited germinal center activity (black arrow heads), which was more extensive in V3000 infected mice compared to V3034 infection. A starry sky pattern (indicated by red arrows) due to increased apoptosis was apparent with V3000 infection. EMH (white arrow head) was observed in V3034 infected spleens unlike V3000 at 72 h

The uninfected spleen displayed normal red and white pulp areas with some lymphoid aggregates in the white pulp containing few small germinal centers (Fig. 1m, n). A small mantle of mature lymphocytes was observed surrounding the germinal centers and occasional macrophages were present in the lymphoid aggregates; large multilobated megakaryocytes and extramedullary hematopoiesis was present predominantly beneath the capsule of the spleen.

At 24 h post infection (pi) significantly enlarged germinal centers were observed in V3000 infected mouse spleens. Germinal centers contained few neutrophils, but significantly increased numbers of macrophages, lymphocytes karryohrexis, apoptosis, and cellular debris (Fig. 1a, g). Similar changes were observed in the V3034 infected spleens; however, the extent of injury was markedly decreased at 24 h pi. (Fig. 1d, j). At 48 h pi, in V3000 infected mice, spleens displayed very active lymphoid aggregates containing markedly enlarged germinal centers. In case of V3034 infected mice spleens, far less germinal center activity was observed in comparison to V3000. Significantly decreased karyorrhexis and activity was evident in germinal center of V3034 infected spleens when compared to V3000 infection at 48 h pi (Fig. 1b, e, h, k). At 72 h pi, germinal centers were less active with V3000 infection in comparison to 48 h pi. A starry sky pattern was observed due to presence of numerous macrophages containing fragments of degenerating cells. Germinal centers in the white pulp appeared to be recovering their normal architecture (Fig. 1c, i). In comparison, spleens of V3034 infected mice at 72 h pi showed little starry sky pattern, inflammation and necrosis (Fig. 1f, l). Germinal center activity was very close to a normal tissue and germinal centers consisted primarily of normal appearing macrophages and lymphocytes. Interestingly, the amount of extra medullary hematopoiesis (EMH) in the subcapsular region of the spleen significantly decreased in the mice infected with V3000 over the progression of time with little to no hematopoiesis present at 72 h pi. However, in mice infected with V3034, the EMH decreased at 48 h pi, but returned to almost normal at 72 h pi (Fig. 1d-f, j-l).

#### VEEV infection in brain

**Fig. 2:**
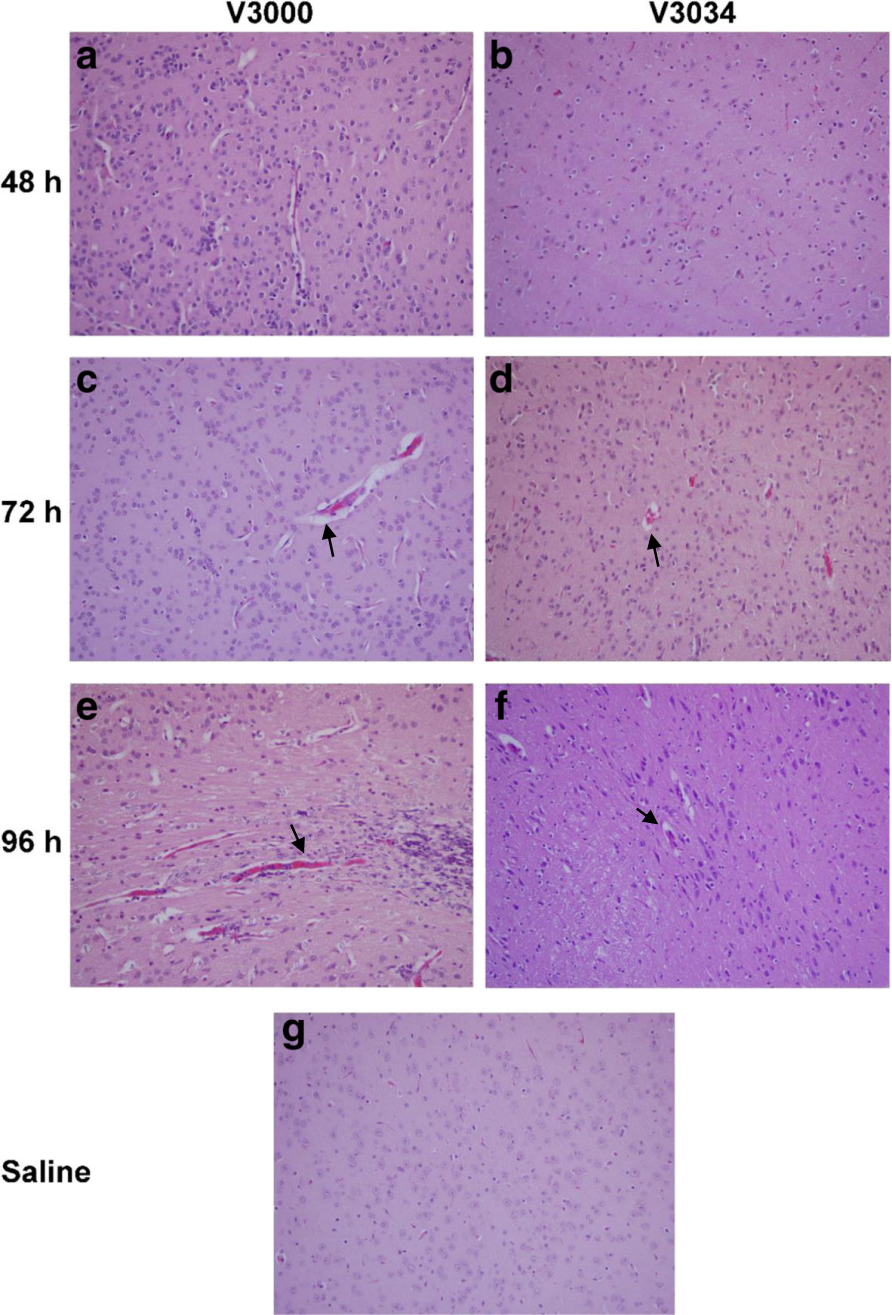
Histology of the brain following infection with V3000 or V3034 strains of VEEV. Brain sections from animals infected with V3000 and V3034 were analyzed by H&E stain. Both the strains induced perivascular inflammation (arrows) and parenchymal inflammation. (**a**) V3000 at 48 h, (**b**) V3034 at 48 h, (**c**) V3000 at 72 h, (**d**) V3034 at 72 h, (**e**) V3000 at 96 h, (**f**) V3034 at 96 h and (**g**) Saline. Magnification: 20X

At 24 h pi, V3000 or V3034 infected mice brain looked similar to that of the uninfected controls with minimal inflammatory response. In case of V3000 infected mice, a mild increase in the cellularity was observed in the brains and progressive parencymal and perivascular inflammation as evidenced by focal mild endothelial cuffing, perivascular edema and parenchymal cellularity, which progressed from 48–72 h pi (Fig. 2a, c). V3034 infection resulted in decreased perivascular inflammation and cellularity as compared to V3000 at 48 h pi (Fig. 2b). At 72 h pi, 3 out of 4 mice brains infected with V3034 showed focal areas of mild inflammation throughout the brain as noted by the endothelial cuffing, edema around vessels, and escaping mononuclear cells in neocortex, striatum, thalamus, and hypothalamus regions of the brain (Fig. 2d). One of the remaining brains showed mild inflammation only in the neocortex region. An increase in inflammation was observed with V3000 infection at 96 h pi demonstrated by both the increase in number of vessels with perivascular cuffing and the degree of escaping mononuclear lymphocytes in comparison to 72 h pi (Fig. 2e). Similar increase in number of inflamed vessels was also observed in all animals infected with V3034 at 96 h pi however, the extent of inflammation was less than that seen in V3000 infection (Fig. 2f). Overall the extent of inflammation was more pronounced and widespread in brains of mice infected with V3000 as compared to those of the mice infected with V3034.

**Fig. 3:**
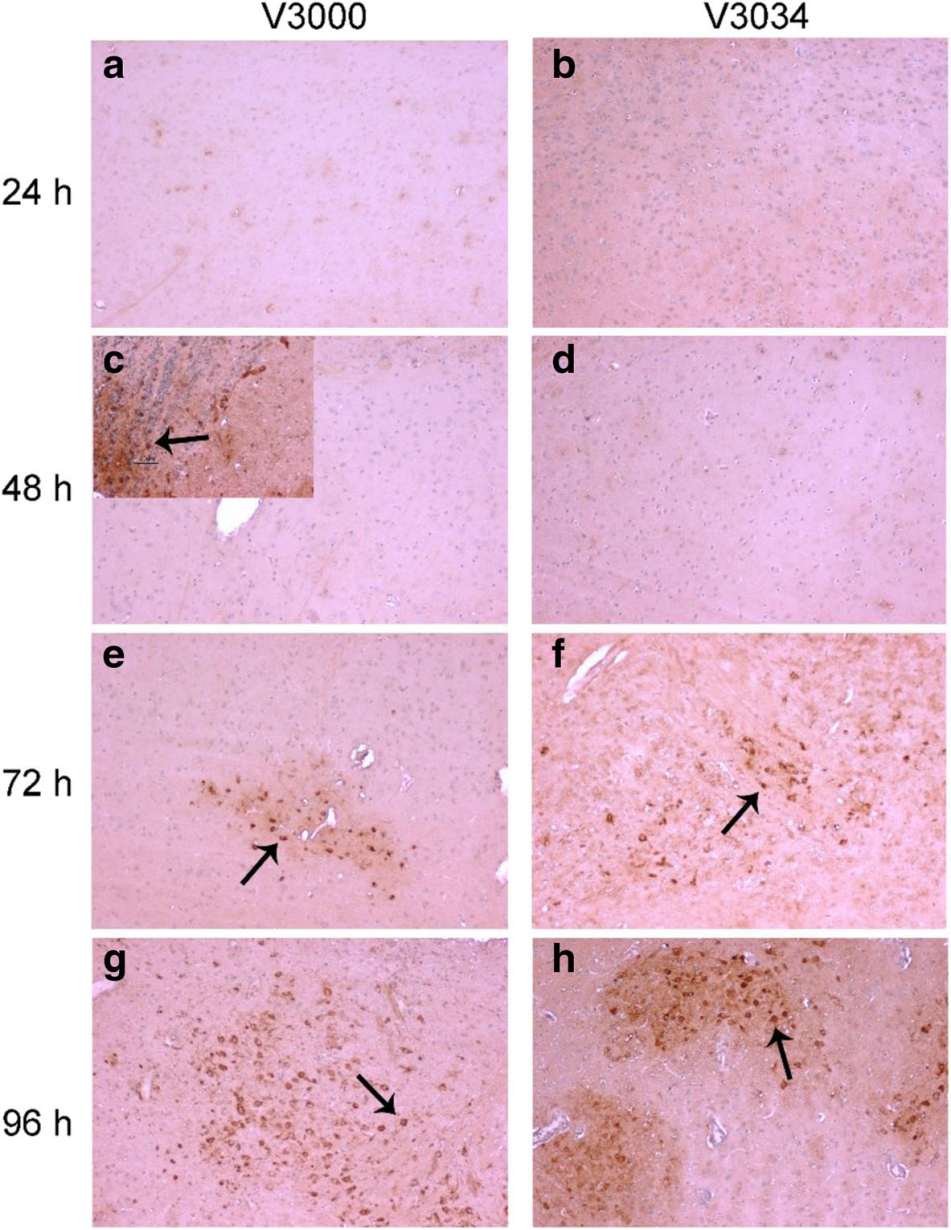
Staining for VEEV antigen in the brain following infection with V3000 or V3034 strains of VEEV. Brain sections from animals infected with V3000 and V3034 were stained with VEEV-specific antibody to detect the presence of virus in the brain. (**a**) V3000 at 24 h, (**b**) V3034 at 24 h, (**c**) V3000 at 48 h, (**d**) V3034 at 48 h, (**e**) V3000 at 72 h, (**f**) V3034 at 72 h, (**g**) V3000 at 96 h and (**h**) V3034 at 96 h. At 48 h pi olfactory lobe (insert) showed positive staining for VEEV. Both the viruses showed positive VEEV staining (arrows) by 72 h pi. V3000 resulted in a more widespread staining in various regions of the brain which increased from 72 h to 96 h pi (hypothalamus shown here). In V3034 infected group at 96 h pi, one brain section showed VEEV staining only in the thalamus region (shown here) and one showed VEEV staining only in the olfactory lobe. Rest of the brain regions and sections were negative for VEEV staining after V3034 infection. Magnification: 20X

No VEEV specific immunohistochemical staining was observed in the brains of mice infected with either V3000 or V3034 at 24 h pi (Fig. 3a, b). At 48 h pi, VEEV staining could be seen in the olfactory lobe of the brains of mice infected with V3000 (Fig. 3c). However, no VEEV specific staining was observed in the brains of mice infected with V3034 at this time point (Fig. 3d). By 72 h pi, small focal VEEV staining was observed largely in neurons in the olfactory bulb, neocortex, striatum and hypothalamus region of the brain in all the animals infected with V3000 (Fig. 3e). Medulla, tegument and thalamus were generally negative for VEEV staining. In mice infected with V3034, 2 out of 3 brain samples were focally positive for VEEV staining at 72 h pi and VEEV staining was concentrated in the thalamus and hypothalamus regions of the brain (Fig. 3f). At 96 h pi, VEEV was significantly more wide-spread involving multiple regions of the brains of mice infected with V3000 (Fig. 3g). In contrast to the extensive staining of V3000 at 96 h pi, brain samples from V3034 infected mice showed only focal positive staining for VEEV predominantly in the olfactory lobe of one mouse and thalamus region of another mouse (Fig. 3g-h). Overall VEEV staining was observed in both V3000 and V3034 infected brain sections, however, was more wide-spread and prominent in mice infected with V3000.

### VEEV strains induced differential host gene expression kinetics.

#### VEEV induced gene expression in spleen

Lymphoid organs such as the lymph nodes and spleen are primary target sites of VEEV replication during the peripheral phase of infection. Both these strains have been shown to infect and replicate well in the spleen [3]. Therefore, to determine the host responses triggered during the peripheral phase of infection by neurovirulent as well as partially neurovirulent strains of VEEV, we evaluated the gene expression profiles in the spleen during infection with V3000 and V3034 strains of VEEV at 24 h, 48 h and 72 h pi. V3000 infection resulted in significant modulation of 324, 531 and 486 gene transcripts in the spleen at 24 h, 48 h and 72 h pi, respectively, compared to the uninfected controls (Table 1). V3034 infection resulted in significant modulation of 89, 505 and 248 gene transcripts in the spleen at 24 h, 48 h and 72 h pi, respectively, compared to the uninfected controls (Table 1). Comparison of the different time points showed that 66 genes with V3000 infection and 16 genes with V3034 infection were similarly modulated at all the three time points (Additional file 1: Figure S1a and b). The number of significantly modulated genes increased at 48 h pi during infection with either of the viral strains, which was concomitant with the increased germinal center activity at 48 h pi for both the viruses.

**Table 1:** Number of significantly modulated genes in spleen and brain

	Spleen			Brain		
	24 h pi	48 h pi	72 h pi	48 h pi	72 h pi	96 h pi
V3000						
Upregulated	136	255	241	75	57	210
Downregulated	188	276	245	166	111	169
Total	324	531	486	241	168	379
V3034						
Upregulated	58	252	210	62	79	71
Downregulated	31	253	38	92	127	153
Total	89	505	248	154	206	224

Total RNA extracted from V3000 and V3034 infected spleen and brain was used to perform whole genome microarray as described in methods. Gene’s modulated in all the replicates (*n* = 3), ≥2.0 fold (*i.e.*, log_2_ ratio ≥ 1.0 or ≤ −1.0) relative expression level over uninfected controls, and *p*-value ≤0.05 were considered significant

#### VEEV induced gene expression in brain

The CNS phase of VEEV replication is characterized by active viral replication in neurons and glia cells in the brain. Therefore, to evaluate the host responses in the brain, gene expression kinetics was evaluated during infection with the neurovirulent (V3000) or the partially neurovirulent (V3034) strains of VEEV at 48 h, 72 h, and 96 h pi. V3000 infection resulted in significant modulation of 241, 168 and 379 gene transcripts at 48 h, 72 h and 96 h pi, respectively (Table 1). V3034 infection resulted in significant modulation of 154, 206 and 224 gene transcripts in brain at 48 h, 72 h and 96 h pi, respectively (Table 1). Comparison of the different time points showed that 62 genes with V3000 infection and 70 genes with V3034 infection were similarly modulated at all the three time points (Additional file 1: Figure S1c and d).

#### VEEV infection induced a subset of genes that was common between both V3000 and V3034 strains.

To identify a host response to VEEV infection irrespective of the virulence of VEEV, the genes that were commonly modulated with both the neurovirulent as well as the partially neurovirulent strains of VEEV were identified. In the spleen, 32, 203 and 48 genes were found to be commonly modulated against both V3000 and V3034 infections at 24 h, 48 h and 72 h pi, respectively. All of the common modulated genes show a similar expression pattern for both the strains of VEEV, except for hemoglobin subunit beta-1 (*Beta-s*) gene, which showed downregulation with V3000 and upregulation with V3034 infections at 72 h pi. A complete list of genes commonly modulated against both the strains at different time points in the spleen is given in Additional file 2: Table S1. Four genes *i.e., Fyb*, *Zbp1*, I *fi27l 2a* and *Slc7a6os* were commonly modulated in spleens of mice infected with either of the VEEV strains at all the three time points studied. In brain, 70, 83 and 121 genes were commonly modulated with both the VEEV strains at 48 h, 72 h and 96 h pi respectively. All the genes displayed similar expression patterns, however, the extent of the modulation varied between infections with the two strains of VEEV. Of the commonly modulated genes, thirty two genes in the brain (such as *Fyb*, *Mst1*, *Ilf3* and *Socs3*) were modulated at all the three time points studied. A complete list of genes commonly modulated against both the VEEV strains at different time points in the brain is given in Additional file 3: Table S2.

#### VEEV infection induced subsets of genes that were unique to either the neurovirulent V3000 strain or the partially-neurovirulent V3034 strain

To identify host responses specific to neurovirulent and partially-neurovirulent VEEV infection, genes that are uniquely modulated with V3000 or V3034 infection were identified. V3000 infection triggered significant modulation of 228, 204 and 316 unique genes at 24 h, 48 h and 72 h pi, respectively, in spleen. The 10 most upregulated genes for each time point are given in Table 2. The remainder of the gene list, including the downregulated genes, is given in Additional file 4: Table S3. Of these, 11 genes (*Socs1*, *Serpina3g*, *LOC100042025*, *Map4k4*, *Olfr819*, *Amfr*, *Vps37a*, *P2ry1*, *Slc12a5*, *Dtx3* and *Trib2*) were commonly modulated at all the three points studied in spleen. In the brain, 122, 59 and 99 genes were uniquely modulated at 48 h, 72 h and 96 h pi respectively, by V3000 infection. The 10 most upregulated genes for each time point are given in Table 3. The remainder of the gene list, including the downregulated genes is given in Additional file 5: Table S4. Only histocompatibility 2, T region locus 23 (*H2-T23*) gene was found to be modulated at all the time points studied in the brain.

**Table 2:** Significantly modulated genes in spleen unique to V3000

UniGene	Gene	Description	Log_2_ Exp ± SEM
Genes uniquely modulated at 24 h pi			
Mm.389688	Oas1g	2′-5′ oligoadenylate synthetase 1G	4.50 ± 0.33
Mm.218770	Ifi202b	Interferon activated gene 202B	4.42 ± 0.41
Mm.130	Socs1	Suppressor of cytokine signaling 1	4.26 ± 0.40
Mm.461583	Zfp456	Zinc finger protein 456	3.77 ± 0.20
Mm.271809	Daxx	Fas death domain-associated protein	3.77 ± 0.16
Mm.377095	Ly6f	Lymphocyte antigen 6 complex, locus F	3.62 ± 0.26
Mm.482110	Ly6c2	Lymphocyte antigen 6 complex, locus C2	3.56 ± 0.18
Mm.312628	Serpina3g	Serine (or cysteine) peptidase inhibitor, clade A, member 3G	3.25 ± 0.12
Mm.20079	Calml3	Calmodulin-like 3	3.06 ± 0.41
Mm.261270	Ifi204	Interferon activated gene 204	2.83 ± 0.06
Genes uniquely modulated at 48 h pi			
Mm.136573	Stfa3	Stefin A3	4.36 ± 0.42
Mm.766	Cxcl9	Chemokine (C-X-C motif) ligand 9	3.85 ± 0.50
Mm.390870	BC117090	CDNA sequence BC1179090	3.76 ± 0.16
Mm.8369	Mst1	Macrophage stimulating 1	3.65 ± 0.34
Mm.276739	Sox10	SRY-box containing gene 10	3.42 ± 0.25
Mm.44176	Efemp1	Epidermal growth factor-containing fibulin-like extracellular matrix protein 1	3.26 ± 0.24
Mm.338001	Pld5	Phospholipase D family, member 5	3.07 ± 0.19
Mm.272115	Myom2	Myomesin 2	3.03 ± 0.17
Mm.1583	Ly6c1	Lymphocyte antigen 6 complex, locus C1	2.92 ± 0.09
Mm.312628	Serpina3g	Serine (or cysteine) peptidase inhibitor, clade A, member 3G	2.91 ± 0.19
Genes uniquely modulated at 72 h pi			
Mm.40965	Nt5c2	5′-nucleotidase, cytosolic II	3.61 ± 0.16
Mm.136573	Stfa3	Stefin A3	3.41 ± 0.38
Mm.312628	Serpina3g	Serine (or cysteine) peptidase inhibitor, clade A, member 3G	3.11 ± 0.32
Mm.482110	Ly6c2	Lymphocyte antigen 6 complex, locus C2	3.10 ± 0.19
Mm.272115	Myom2	Myomesin 2	2.98 ± 0.28
Mm.20079	Calml3	Calmodulin-like 3	2.92 ± 0.26
Mm.359610	Stfa2	Stefin A2	2.86 ± 0.38
Mm.276389	Hmox1	Heme oxygenase (decycling) 1	2.47 ± 0.25
Mm.461583	Zfp456	Zinc finger protein 456	2.29 ± 0.41
Mm.34609	Plac8	Placenta-specific 8	2.28 ± 0.09

Genes that were uniquely modulated against V3000 infection in the spleen were identified. The list summarizes 10 genes with maximum amount of upregulation for each time point. Values are expressed as average values of (log_2_) fold expression for each gene over uninfected controls ± standard error mean (SEM). * *P* ≤ 0.05

**Table 3:** Significantly modulated genes in brain unique to V3000 infection

UniGene	Gene	Description	Log_2_ Exp ± SEM
Genes uniquely modulated at 48 h pi			
Mm.40965	Nt5c2	5′-nucleotidase, cytosolic II	3.44 ± 0.61
Mm.461583	Zfp456	Zinc finger protein 456	2.79 ± 0.56
Mm.341423	Dock4	Dedicator of cytokinesis 4	2.16 ± 0.05
Mm.338001	Pld5	Phospholipase D family, member 5	1.90 ± 0.21
Mm.33902	Igtp	Interferon gamma induced GTPase	1.84 ± 0.26
Mm.211477	Phldb2	Pleckstrin homology-like domain, family B, member 2	1.76 ± 0.13
Mm.247453	Abhd10	Abhydrolase domain containing 10	1.50 ± 0.22
Mm.6856	Pttg1	Pituitary tumor-transforming gene 1	1.46 ± 0.04
Mm.290764	Lrrc61	Leucine rich repeat containing 61	1.43 ± 0.26
Mm.442861	H2-D4	Histocompatibility 2, D region locus 4	1.40 ± 0.15
Genes uniquely modulated at 72 h pi			
Mm.141021	Ifitm3	Interferon induced transmembrane protein 3	2.98 ± 0.37
Mm.425949	Ly6a	Lymphocyte antigen 6 complex, locus A	2.64 ± 0.46
Mm.44176	Efemp1	Epidermal growth factor-containing fibulin-like extracellular matrix protein 1	2.44 ± 0.17
Mm.439743	H2-Q7	Histocompatibility 2, Q region locus 7	2.35 ± 0.13
Mm.132226	Ehd4	EH-domain containing 4	2.05 ± 0.30
Mm.41075	Drd4	Dopamine receptor 4	1.97 ± 0.15
Mm.39825	Lmx1b	LIM homeobox transcription factor 1 beta	1.84 ± 0.12
Mm.131422	Osm	Oncostatin M	1.67 ± 0.26
Mm.475107	Rtp4	Receptor transporter protein 4	1.51 ± 0.10
Mm.69751	Pepd	Peptidase D	1.44 ± 0.11
Genes uniquely modulated at 96 h pi			
Mm.4950	Isg15	ISG15 ubiquitin-like modifier	4.04 ± 0.46
Mm.425949	Ly6a	Lymphocyte antigen 6 complex, locus A	3.89 ± 0.12
Mm.284248	Ccl5	Chemokine (C-C motif) ligand 5	3.76 ± 0.69
Mm.290320	Ccl2	Chemokine (C-C motif) ligand 2	2.80 ± 0.25
Mm.24045	Rsad2	Radical S-adenosyl methionine domain containing 2	2.74 ± 0.57
Mm.133342	Rnf213	Ring finger protein 213	2.66 ± 0.37
Mm.24125	Col4a3bp	Collagen, type IV, alpha 3 (Goodpasture antigen) binding protein	2.59 ± 0.43
Mm.426537	LOC100041885	PREDICTED: similar to C130026I21Rik protein	2.45 ± 0.19
Mm.439648	H2-T23	Histocompatibility 2, T region locus 23	2.32 ± 0.44
Mm.357727	Gm46	PREDICTED: gene model 46, (NCBI)	2.31 ± 0.23

Genes that were uniquely modulated against V3000 infection in the brain were identified. The list summarizes 10 genes with maximum amount of upregulation for each time point. Values are expressed as average values of (log_2_) fold expression for each gene over uninfected controls ± standard error mean (SEM). * *P* ≤ 0.05

In the spleen, V3034 infection resulted in modulation of 46, 198 and 172 unique genes at 24 h, 48 h and 72 h pi, respectively. The top 10 most upregulated genes for each time point are given in Table 4. The remainder of the gene list, including the downregulated genes, is given in Additional file 6: Table S5. In the brain, V3034 infection resulted in modulation of 55, 89 and 51 genes unique genes at 48 h, 72 h and 96 h pi, respectively. The top 10 most upregulated genes for each time point are given in Table 5. The remainder of the gene list, including the downregulated genes, is given in Additional file 7: Table S6. Only 3 genes each in the spleen (*B2m*, *Lst1* and *Edf1*) and the brain (*Six6*, *Cd163* and *Bmp10*) were modulated at all three time points after V3034 infection.

**Table 4:** Significantly modulated genes in spleen unique to V3034 infection

UniGene	Gene	Description	Log_2_ Exp ± SEM
Genes uniquely modulated at 24 h pi			
Mm.276926	Prss2	Protease, serine, 2	5.61 ± 0.83
Mm.45316	Cela2a	Chymotrypsin-like elastase family, member 2A	5.17 ± 0.20
Mm.297477	Cela3b	Chymotrypsin-like elastase family, member 3B	5.10 ± 0.29
Mm.243758	Apol9a	Apolipoprotein L 9a	3.89 ± 0.66
Mm.4950	Isg15	ISG15 ubiquitin-like modifier	3.47 ± 0.38
Mm.33902	Igtp	Interferon gamma induced GTPase	3.10 ± 0.39
Mm.23067	Ufm1	Ubiquitin-fold modifier 1	2.43 ± 0.49
Mm.32881	Spnb1	Spectrin beta 1	2.19 ± 0.45
Mm.439732	C1qc	Complement component 1, q subcomponent, C chain	1.91 ± 0.32
Mm.259916	Chdh	Choline dehydrogenase	1.86 ± 0.08
Genes uniquely modulated at 48 h pi			
Mm.9714	Gdf9	Growth differentiation factor 9	4.80 ± 1.03
Mm.376121	Olfr1152	Olfactory receptor 1152	3.57 ± 0.49
Mm.132226	Ehd4	EH-domain containing 4	2.71 ± 0.22
Mm.386931	Ecm2	Extracellular matrix protein 2, female organ and adipocyte specific	2.25 ± 0.04
Mm.302516	Fsip2	PREDICTED: fibrous sheath-interacting protein 2	2.19 ± 0.34
Mm.483584	Serpina3f	Serine (or cysteine) peptidase inhibitor, clade A, member 3F	2.08 ± 0.20
Mm.447	Pyhin1	Pyrin and HIN domain family, member 1	2.04 ± 0.28
Mm.265806	Ankrd13c	Ankyrin repeat domain 13c	1.89 ± 0.12
Mm.90450	Myo1a	Myosin IA	1.84 ± 0.02
Mm.41337	Akr1c18	Aldo-keto reductase family 1, member C18	1.84 ± 0.14
Genes uniquely modulated at 72 h pi			
Mm.297477	Cela3b	Chymotrypsin-like elastase family, member 3B	6.33 ± 0.59
Mm.45316	Cela2a	Chymotrypsin-like elastase family, member 2A	6.24 ± 0.26
Mm.485316	Gm5409	Predicted pseudogene 5409	6.11 ± 0.50
Mm.276926	Prss2	Protease, serine, 2	6.01 ± 0.55
Mm.8369	Mst1	Macrophage stimulating 1	5.87 ± 0.50
Mm.212333	Pnliprp2	Pancreatic lipase-related protein 2	5.59 ± 0.66
Mm.389688	Oas1g	2′-5′ oligoadenylate synthetase 1G	4.85 ± 0.45
Mm.245154	Tmeff2	Transmembrane protein with EGF-like and two follistatin-like domains 2	4.32 ± 0.45
Mm.2745	Ctrl	Chymotrypsin-like	3.77 ± 0.67
Mm.243758	Apol9a	Apolipoprotein L 9a	3.27 ± 0.23

Genes that were uniquely modulated against V3034 infection in the spleen were identified. The list summarizes 10 genes with maximum amount of upregulation for each time point. Values are expressed as average values of (log_2_) fold expression for each gene over uninfected controls ± standard error mean (SEM). * *P* ≤ 0.05

**Table 5:** Significantly modulated genes in brain unique to V3034 infection

UniGene	Gene	Description	Log_2_ Exp ± SEM
Genes uniquely modulated at 48 h pi			
Mm.40655	Fam185a	Family with sequence similarity 185, member A	2.97 ± 0.56
Mm.57138	Six6	Sine oculis-related homeobox 6 homolog	2.57 ± 0.57
Mm.141021	Ifitm3	Interferon induced transmembrane protein 3	2.54 ± 0.31
Mm.46418	Chia	Chitinase, acidic	2.34 ± 0.38
Mm.44176	Efemp1	Epidermal growth factor-containing fibulin-like extracellular matrix protein 1	1.79 ± 0.28
Mm.103748	Extl3	Exostoses (multiple)-like 3	1.62 ± 0.28
Mm.207062	Hoxc13	Homeobox C13	1.59 ± 0.28
Mm.28162	Nup210	Nucleoporin 210	1.53 ± 0.16
Mm.41075	Drd4	Dopamine receptor D4	1.51 ± 0.22
Mm.42150	Rasgrp1	RAS guanyl releasing protein 1	1.44 ± 0.31
Genes uniquely modulated at 72 h pi			
Mm.40965	Nt5c2	5′-nucleotidase, cytosolic II	4.22 ± 0.85
Mm.461583	Zfp456	Zinc finger protein 456	3.18 ± 0.52
Mm.196581	Mapk1	Mitogen-activated protein kinase 1	2.75 ± 0.29
Mm.57138	Six6	Sine oculis-related homeobox 6 homolog	2.70 ± 0.37
Mm.460823	Gm10731	Predicted gene 10,731	2.66 ± 0.29
Mm.357727	Gm46	PREDICTED: predicted gene 46	2.66 ± 0.45
Mm.364180	5033411D12Rik	RIKEN cDNA 5033411D12 gene	2.54 ± 0.37
Mm.426537	LOC100041885	PREDICTED: sp110 nuclear body protein-like	2.50 ± 0.40
Mm.245154	Tmeff2	Transmembrane protein with EGF-like and two follistatin-like domains 2	2.13 ± 0.30
Mm.276739	Sox10	SRY-box containing gene 10	1.99 ± 0.35
Genes uniquely modulated at 96 h pi			
Mm.57138	Six6	Sine oculis-related homeobox 6 homolog	3.24 ± 0.13
Mm.196581	Mapk1	Mitogen-activated protein kinase 1	2.85 ± 0.19
Mm.196013	Samd9l	Sterile alpha motif domain containing 9-like	2.21 ± 0.08
Mm.41075	Drd4	Dopamine receptor D4	2.18 ± 0.13
Mm.32881	Spnb1	Spectrin beta 1	2.02 ± 0.18
Mm.323595	Tob2	Transducer of ERBB2, 2	1.76 ± 0.19
Mm.256414	Slc9a2	Solute carrier family 9 (sodium/hydrogen exchanger), member 2	1.64 ± 0.13
Mm.269029	Slc7a6os	Solute carrier family 7, member 6 opposite strand	1.61 ± 0.24
Mm.34428	Pias4	Protein inhibitor of activated STAT 4	1.38 ± 0.02
Mm.271745	Nrp1	Neuropilin 1	1.30 ± 0.08

Genes that were uniquely modulated against V3034 infection in the brain were identified. The list summarizes 10 genes with maximum amount of upregulation for each time point. Values are expressed as average values of (log_2_) fold expression for each gene over uninfected controls ± standard error mean (SEM). * *P* ≤ 0.05

#### Real time PCR validation.

The microarray gene expression validation was completed by real time RT-PCR for randomly selected genes at different time points in the brain samples. *Stat1*, *Stat2*, *NfκB2, Zfp456* and *Nt5c2* from the V3000-unique gene list, were shown to have similar expression pattern in both microarrays and real time PCR for the V3000 infected brain samples. In addition, *Samd9l* belonging to the VEEV specific subset, was found significantly up-regulated at 48 h pi in the V3000 and V3034 infected brain samples by both microarrays and real time PCR (Additional file 1: Figure S2 and Additional file 8: Table S7).

### Signaling pathway analysis of genes modulated during VEEV infection

#### Pathway analysis of the genes uniquely expressed during infection with either V3000 or V3034

**Fig. 4:**
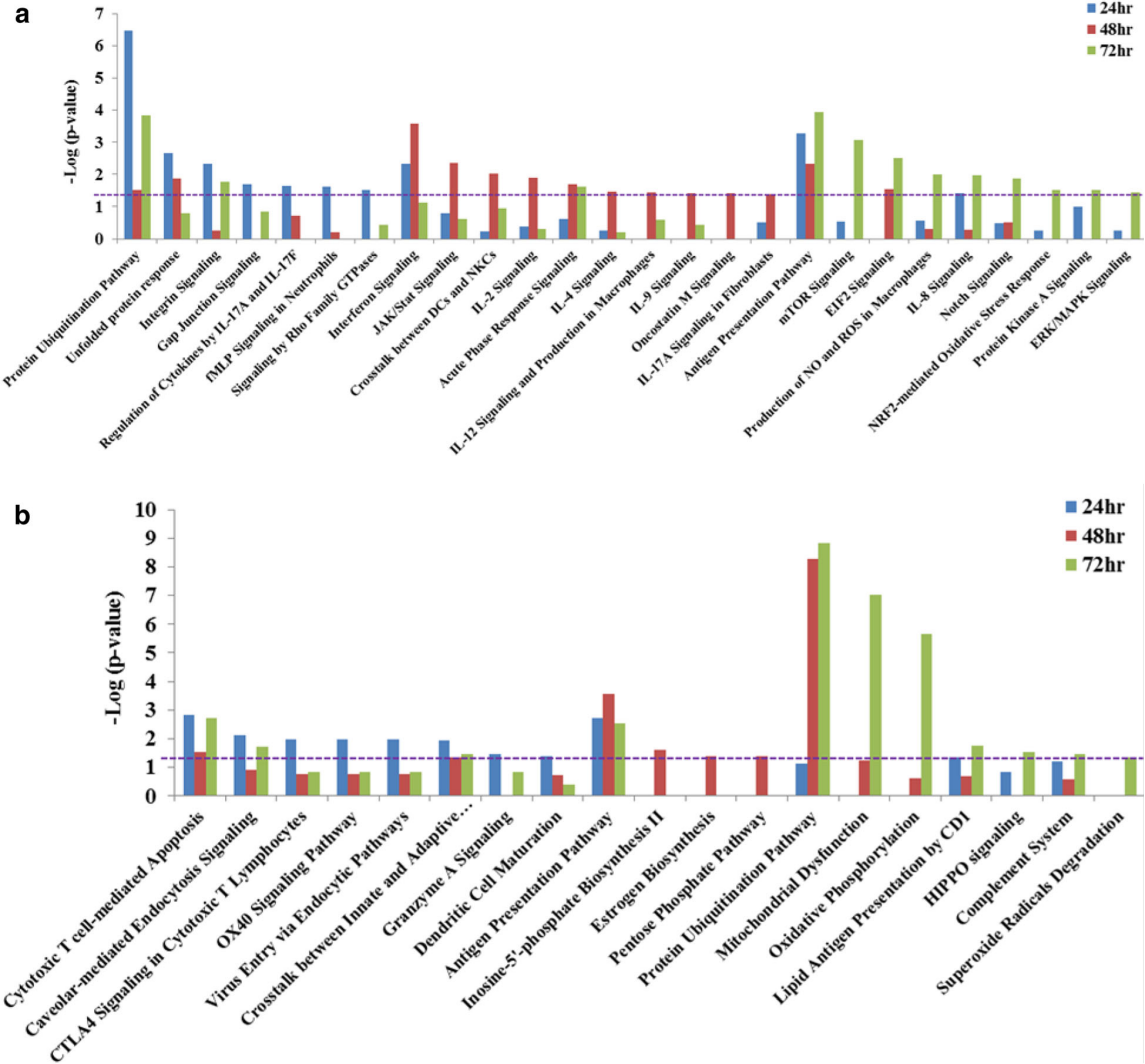
Pathway analysis of genes modulated in the spleen in response to virus infections. Unique significantly modulated genes in the spleen with (**a**) V3000 and (**b**) V3034 infection were analyzed by Ingenuity Pathway analysis software. Pathways significantly modulated during at least one of the three time points studied are shown here. Y-axis represents the level of significance as –log (*p*-value), therefore, the height of bar is directly proportional to the level of significance. Purple dotted line indicates *p*-value =0.05

Pathway analysis was performed on the genes uniquely modulated against V3000 and V3034 infection in the spleen and brain using the Ingenuity Pathway Analysis (IPA) software suit (Fig. 4). Antigen presentation and protein ubiquitination pathways were found to be commonly modulated by V3000 and V3034 infection in the spleen. The antigen presentation pathway was significantly modulated at all the time points by both virus infections. Protein ubiquitination pathway was significantly modulated at 24 h pi with V3000 infection and remained so for the remainder of the time points (Fig. 4a). In V3034 infection, the protein ubiquitination pathway was significantly modulated only at 48 h and 72 h pi (Fig. 4b). Some pathways were unique to infection with each of the strains of VEEV. V3000 infection uniquely modulated several inflammatory signaling pathways such as IL-2, IL-4, IL-8, IL-9, IL-12 and IL-17A along with the unfolded protein response, gap junction signaling, crosstalk between DCs and NKCs, interferon and JAK/Stat signaling pathways (Fig. 4a). On the other hand, pathways such as virus entry via the endocytic pathway, or granzyme A signaling, dendritic cell maturation and complement system pathways were found to be significantly modulated only during V3034 infection (Fig. 4b).

**Fig. 5:**
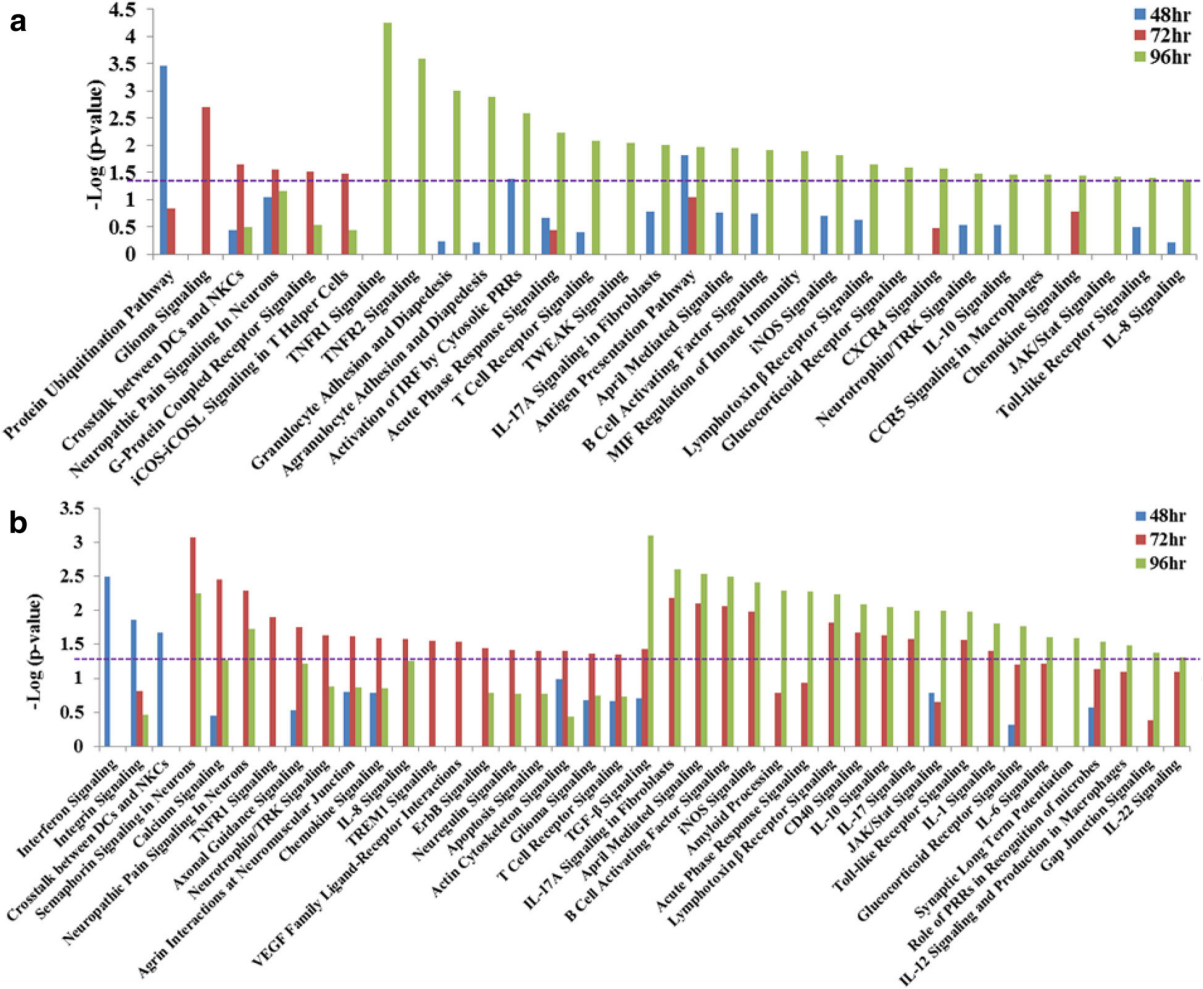
Pathway analysis of genes modulated in the brain in response to virus infections. Unique significantly modulated genes in the brain with (**a**) V3000 and (**b**) V3034 infection were analyzed by Ingenuity Pathway analysis software. Pathways significantly modulated during at least one of the three time points studied are shown here. Y-axis represents the level of significance as –log (p-value). Purple dotted line indicates *p*-value =0.05

In the brain, pathway analysis of uniquely modulated genes identified pathways such as, glioma, neuropathic pain, acute phase, JAK/Stat, Toll-like Receptor (TLR), chemokine and T-cell receptor (TCR) signaling pathways to be similarly modulated during infection with either of the VEEV strains (Fig. 5). Some of these pathways such as glioma, neuropathic pain, and acute phase response signaling showed significant modulation at the same points in both viral strains. In contrast, pathways such as TCR, tumor necrosis factor receptor1 (TNFR1), and chemokine signaling showed differential kinetics in response to the infection with the two viral strains (Fig. 5a and b). Similar to the observations in spleen, several pathways were found to be significantly modulated during infection with only one of the VEEV strains in the brain as well. Pathways such as protein ubiquitination pathway, activation of interferon regulatory factors (IRFs) by cytosolic pattern recognition receptors (PRRs), CXC chemokine receptor4 (CXCR4) and C-C motif Chemokine Receptor 5 (CCR5) signaling in macrophages were found to be significantly modulated only with V3000 infection (Fig. 5a). Pathways such as interferon, axonal guidance, synaptic long term potentiation, integrin, calcium, actin cytoskeleton and gap junction signaling were significantly modulated only with V3034 infection (Fig. 5b).

#### Network analysis of differentially modulated genes during infection with V3000 and V3034 strains

**Fig. 6:**
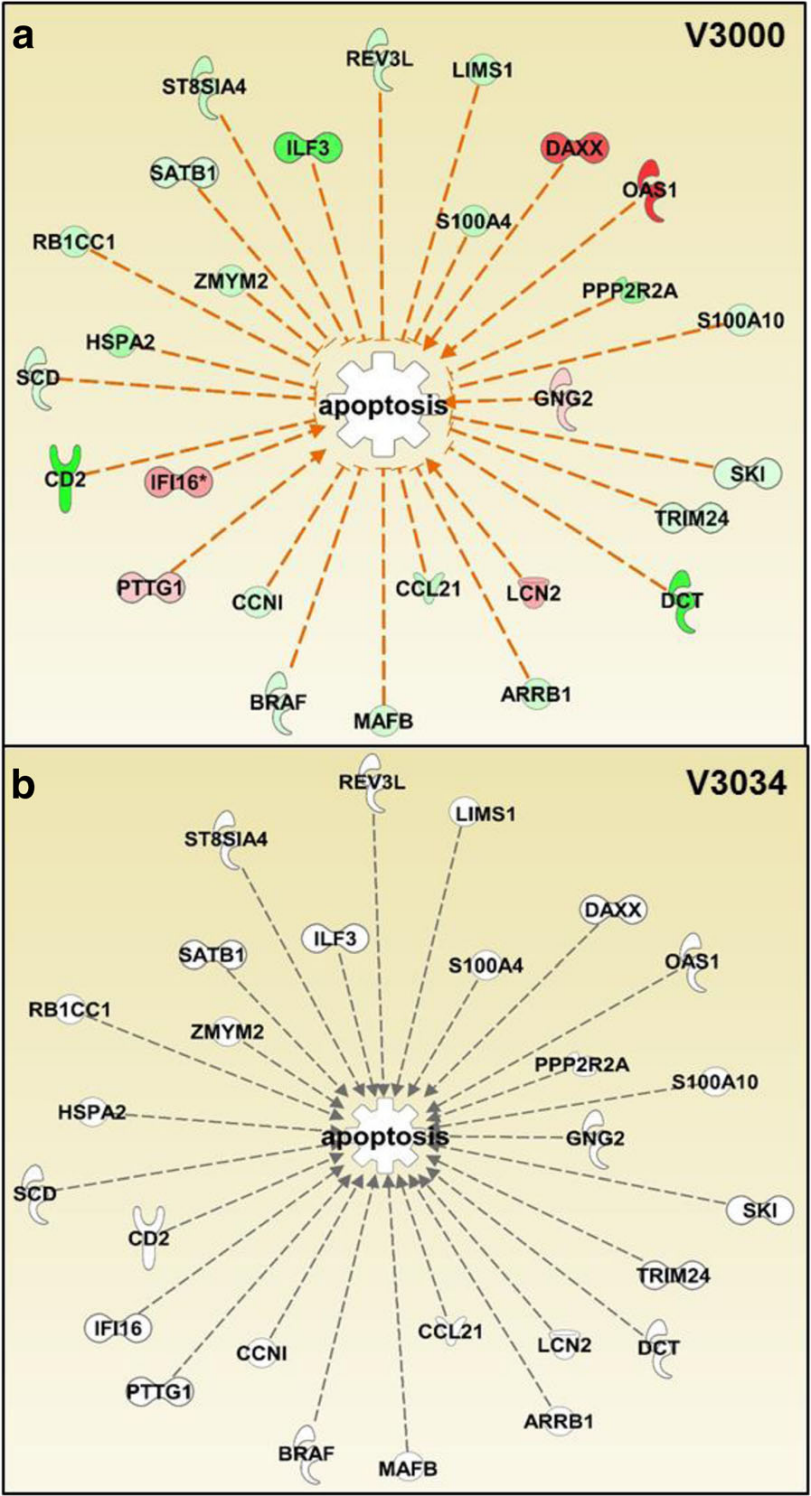
Network analysis of apoptotic genes modulated in response to V3000 and V3034 infections in spleen at 24 h pi. *In silico* network analysis of significantly modulated genes in the spleen was performed for the apoptotic pathway using the Ingenuity Pathway analysis software. Analysis showed modulation of the apoptotic pathway in V3000 infection (**a**) mediated by upregulation of inducers and downregulation of repressors of apoptotic pathway. In the case of V3034 infection (**b**), such involvement of the apoptotic pathway was not observed. Legends: : leads to activation, : effect not predicted, : upregulated, : downregulated, : not expressed; intensity of the color is directly proportional to the level of modulation

**Fig. 7:**
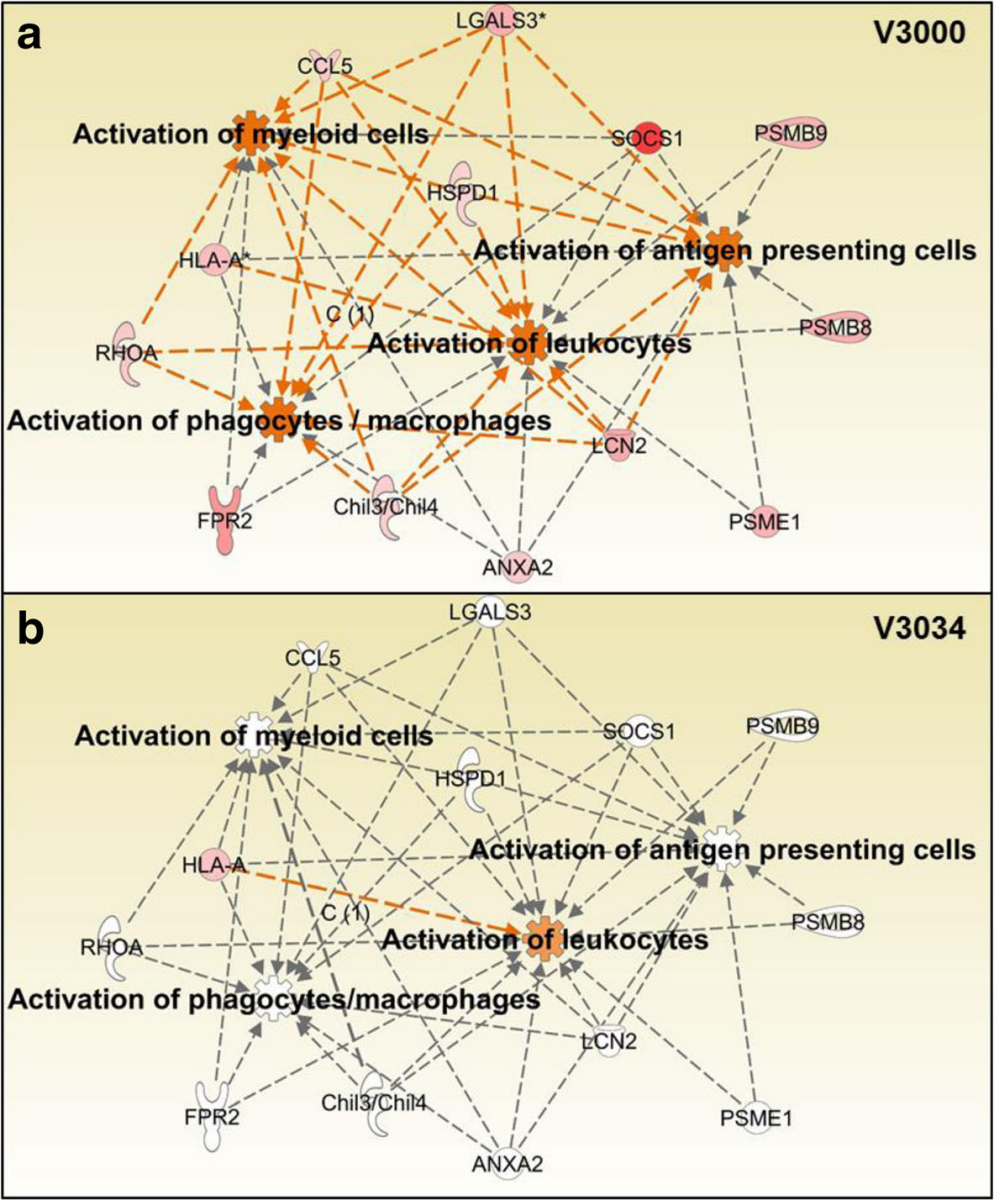
Network analysis of inflammatory genes modulated in response to V3000 and V3034 infections in spleen. Genes significantly modulated against (**a**) V3000 and (**b**) V3034 infection in the spleen were used to perform *in silico network* analysis using the Ingenuity Pathway analysis software. V3000 infection resulted in increased inflammation by upregulating the genes associated with activation of myeloid cells, antigen presenting cells, phagocytes/macrophages and leukocytes unlike V3034, which only resulted in activation of leukocytes at 24 h pi. Legends: : leads to activation, : effect not predicted, : upregulated, : downregulated, : not expressed; intensity of the color is directly proportional to the level of modulation

The molecule activity predictor (MAP) tool of IPA was used to generate gene networks overlaid with the relative gene expression values from the microarray data to identify differences in apoptosis, inflammation, and activation of immune cells against V3000 and V3034 infections. The software predicted overall activation or inhibition of various pathways based on the level of confidence by comparing gene expression values from the microarray data and the available published literature related to the gene interactions. Gene network analysis showed clear differences in the kinetics of apoptotic pathway in spleen with V3000 and V3034 infections. Various repressors of apoptosis such as *Ilf3*, *Zmym2*, *Satb1*, *Ccl21* and *Scd* were downregulated and expression levels of inducers of apoptosis such as *Daxx*, *Oas1*, *Lcn2* and *Gng2* were upregulated with V3000 infection at 24 h pi resulting in an overall activation of the apoptotic pathway (Fig. 6). None of these genes were modulated by V3034 infection at 24 h pi. However, by 48 h and 72 h pi, the apoptotic network was predicted to be activated in spleen during infection with both the viral strains, however, the number of genes involved in apoptosis was less in V3034 as compared to V3000 at 72 h pi (Additional file 1: Figure S3). Inflammation represented by activation of various immune cells showed similar changes in the spleen. The spleen histology showed activation of macrophages in the germinal centers, which was more pronounced in case of V3000 infection as compared to the infection with V3034 at all the time points suggesting a difference in the kinetics of host response against infection by the two strains of VEEV. At 24 h pi, network analysis of genes that predict activation of phagocytes, macrophages, leukocytes, and antigen presenting cells were upregulated after infection with V3000 (Fig. 7). For example, V3000 infection resulted in significant upregulation of *Lcn2* gene (also known as *NGAL*) which is expressed in a variety of cells and tissues including neutrophils, and has been implicated in autoimmune diseases, infections, inflammation, musculoskeletal diseases, metabolic diseases and is being investigated in a clinical trial as a biomarker for acute kidney injury [13]. Upregulation of *Lcn2* along with *RhoA*, *Ccl5*, *Lgals3*, *HspD1* and *Chil3/4* were predicted to activate myeloid and antigen presenting cells. In case of V3034 infection, these cells are not predicted to be activated due to lack of modulation in the expression of these genes at 24 h pi. Due to modulation of, *HLA-A,* only activation of leukocytes was predicted with V3034 infection. At 48 h and 72 h pi, both V3000 and V3034 infections resulted in activation of various immune cells due to modulation of a larger number of genes by both the viruses (Additional file 1: Figure S4).

**Fig. 8:**
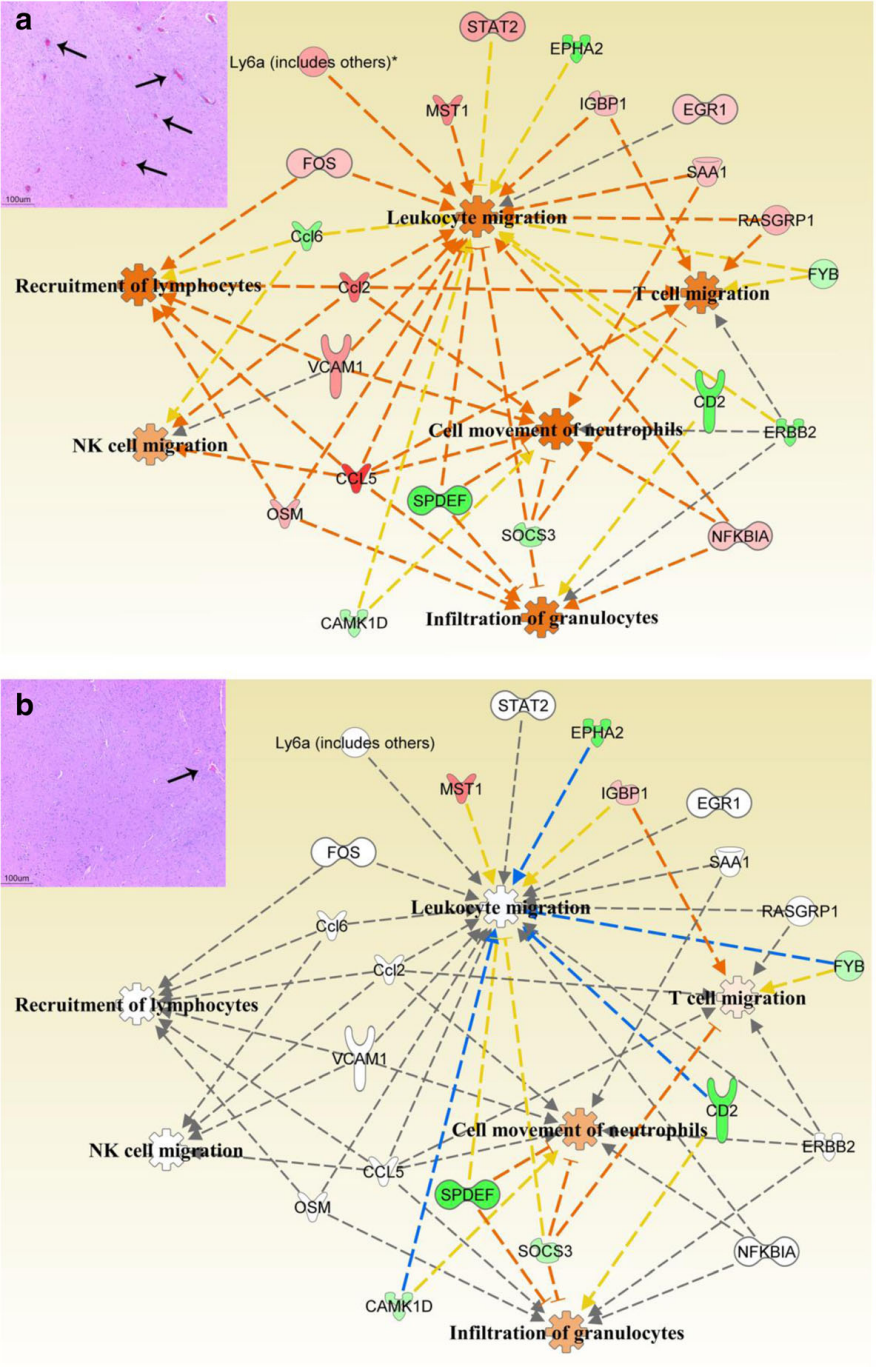
Network analysis of inflammatory genes modulated in response to V3000 and V3034 infections in brain. Genes significantly modulated against (**a**) V3000 and (**b**) V3034 infection in brain at 96 h pi were used to perform *in silico network* analysis using the Ingenuity Pathway analysis software. V3000 infection resulted in enhanced inflammation by significantly upregulating the genes causing leukocyte migration, T cell migration, recruitment of lymphocytes, NK cell migration, infiltration of granulocytes and neutrophil movement. V3034, on the other hand, only resulted in enhanced infiltration of granulocytes and neutrophil cell movement to some extent. Legends: : leads to activation, : leads to inhibition, : finding inconsistent with the state of downstream molecule, : effect not predicted, : upregulated, : downregulated, : not expressed; intensity of the color is directly proportional to the level of modulation

Histological evaluation of the brain in our study demonstrated notable differences in inflammation and mononuclear cell infiltration into the brain as evident by perivascular cuffing (Fig. 8 inserts). Therefore, specific pathway analysis was done for inflammation and immune cell migration to identify genes that may play important roles in differential regulation of these pathways with infection of neurovirulent and partially-neurovirulent strains of VEEV. A clear correlation of modulation of inflammatory pathways and the histology data was observed at 96 h pi. A greater numbers of genes were found to be modulated during infection with V3000 and accordingly inflammatory pathway and immune cell migration pathways were predicted to be significantly more affected during V3000 infection (Fig. 8a). In comparison, during V3034 infection, only the neutrophil migration pathway was predicted to be significantly modulated, other pathways such as movement/recruitment of T lymphocytes, monocytes and granulocytes were not predicted to have any significant changes (Fig. 8b). This is in accordance with the reduced inflammatory response observed by histological staining in V3034 infected brain sections as compared to the V3000 infected brains.

## Discussion

Lethal infection of VEEV results in neuronal cell death, increased cellularity, and an intense inflammatory response characterized by perivascular and interstitial mononuclear infiltrate in the brain, which ultimately results in death [3, 6, 7, 14–16]. In the case of avirulent or partially virulent VEEV infections, the virus is generally cleared from the brain tissue with mild clinical symptoms and therefore, infection is usually not fatal [3]. Host factors that may play role in restricting VEEV replication during infection with avirulent or partially virulent strains are not well understood. Understanding of the molecular changes induced in the host upon VEEV infection resulting in either restriction or progression of viral replication and neurovirulence can identify potential host derived targets for drug development. Therefore, in this study, we compared host responses against two VEEV strains, V3000 and V3034 that reach the host brain, but show difference in neuropathology and lethality.

V3000 and V3034 have been shown to replicate in the spleen of mice [3]. In this study we found that the extent of spleen pathology was markedly different in between the two strains. V3000 infection induced a higher degree of germinal center activation that peaked at 48 h pi. V3034 infection also followed this pattern, however, degree of germinal center activation was significantly less at each time point in comparison with V3000 infection. These observations correlate with the increased number of genes significantly modulated in the spleen with VEEV infection at the three time points. Pathway and network analyses identified a stronger apoptotic and inflammatory response with V3000 infection at 24 h pi as compared to V3034 infection which was similar to the observed histologic changes in these tissues. Both strains induced a similar pattern of modulation among common genes except for that of *Beta-s*, which was down-regulated during V3000 infection but upregulated during V3034 infection. *Beta-s* also known as *Hbb* encodes for beta-globulin that form part of the hemoglobin complex in red blood cells. Interestingly, increase in *Beta-s* expression corresponds with the increased EMH in the spleen of V3034 infected mice at 72 h pi. More detailed study will be needed to establish direct role of *Beta-s* in promoting EMH in the spleen during VEEV infection. Genes such as *Ifi27l2a,* which regulate immune cell influx [17], and *Ifitm3*, which has antiviral function [18], were upregulated in both V3000 and V3034 infection suggesting there role in antiviral response during infection with VEEV. *Ifitm3* has been reported to be upregulated in spleen during VEEV-TrD infection in macaques [19] and has been shown to play a critical role in protection against several viral infections [18, 20, 21]. Taken together, these observations indicate that although both the strains trigger host-mediated defense responses which probably help in clearance of the virus from spleen, the highly virulent V3000 strain induced a stronger inflammatory response which may result in the more extensive pathology identified in the spleen tissue as compared to the less virulent V3034 strain.

Neurovirulence is the hallmark of lethal VEEV infection. Replication of VEEV in the neurons and glia, and associated inflammatory response leads to extensive encephalitis and death of animals [3, 6, 7, 14–16]. Both V3000 and V3034 have been shown to enter the CNS. V3000 causes extensive neuronal death and inflammation of CNS leading to 100% mortality in mice, whereas V3034 only causes death in 11% of the infected mice [3]. Several host genes were commonly modulated against the neurovirulent as well as partially-neurovirulent strain of VEEV. Genes like *B2m*, *Mst1*, *Igsf1*, *Ifitm3* and *Nt5c2* were found to be upregulated whereas genes like *Fyb*, *Ilf3, Wnt4* and *Socs3* were found to be downregulated in the brain against both V3000 and V3034 infections. *Mst1*, a serine/threonine kinase activated by caspases during apoptosis, has been shown to be critical to influenza A virus replication [22]. *Mst1* has also been shown to prevent autophagy and facilitates neurodegeneration during amyotrophic lateral sclerosis [23, 24]. *Mst1* was upregulated in spleen during V3000 and V3034 infection and therefore, likely may contribute positively towards VEEV replication in the brain and spleen. *Ifitm3* is an antiviral protein that has been shown to induce innate immune response against influenza A, West nile, rift valley fever, dengue and vesicular stomatitis viruses [18, 20, 25, 26]. *Ifitm3* was also upregulated in spleen and brains of both V3000 and V3034 infected mice. *Socs3* expression has been shown to reduce the virus replication, and in this study its downregulation in both V3000 and V3034 infected mice brain and spleen suggests that VEEV may inhibit *Socs3* expression for its own replication, which is also reflected in the brain histology that shows active replication of V3000 and V3034 in the brains of infected mice [27, 28]. We have previously shown that VEEV infection increases the expression of *B2m* and *H2Q7*, which are involved in the antigen presentation pathway [8]. In this study, *B2m*, *H2Q7* and *H2Q6* expression was upregulated by both the viral strains suggesting a common innate immune response to both the viral strains. Pathway analyses also identified activation of several innate immune pathways such as crosstalk between DCs and NKCs, T cell receptor, acute phase response, chemokine, JAK/stat and TLR signaling pathways against both the virulent and partially-virulent VEEV strains suggesting these to be common immune responses to VEEV infection, although the kinetics of their modulation varied with the strain of VEEV. Surprisingly, V3034 resulted in modulation of a larger number of host signaling pathways at 72 h pi. This probably suggests a much stronger host response during a partially virulent VEEV infection, which may help in controlling the virus infection during the later time points.

Activation of inflammatory cells is critical to virus clearance however; and the excessive inflammation in the brain caused by VEEV has been implicated in neurodegeneration [6, 7, 9, 12]. Several chemokines *Ccl2*, *Ccl5* and *Ccl6*, and genes from the lymphocyte antigen 6 superfamily, *Ly6a*, *Ly6e* and *Ly6f* were found to be modulated only with V3000 infection in brain. This correlates with the higher degree and more extensive inflammation that was observed in the brains of V3000 infected mice. We have earlier reported the upregulation of Ccl2 in the brains of mice infected with V3000 [10]. Ccl2 which is also known as monocyte chemoattractant protein-1 (MCP-1) has also been implicated in encephalitis caused by viruses including VEEV, and has been shown to play role in permeability of blood brain barrier and leukocyte migration into the brain parenchyma [29–33]. Ccl6 is expressed by the microglia and regulates their migration in the normal brain [34]. Ccl6 expression is also increased in the CNS during inflammatory demyelinating diseases and it acts as a chemoattractant for leukocytes [35]. In addition, Ccl6 has been implicated in encephalitis induced by West Nile virus [36]. Lymphocyte antigen 6 (Ly6) gene encodes for lycosyl-phosphatidyl-inositol anchored cell-surface proteins [37]. Function of Ly6 superfamily of genes have not been completely defined, however, some of these genes have been implicated in various immune response functions such as T-cell homing and activation, and regulation of the complement attack complex [38, 39]. More specifically, *Ly6a*, also known as Stem cell antigen-1 (*Sca-1*) or T-cell activating protein (*TAP*), is expressed on hematopoietic stem cells and has been shown to be involved in T-cell development and cell-cell adhesion [40, 41]. *Ly6e*, also known as *Sca-2* or Thymic Shared Antigen-1 (*TSA-1* ), has been identified as a biomarker for systemic lupus erythematosus and shown to be involved in cell-cell adhesion, as well as B-cell and T-cell regulation [42–45]. Ly6 genes were preferentially upregulated in V3000 infected brains (as well as spleens), and may play a role in microvascular inflammation and mononuclear cell migration in the brain which was markedly increased during V3000 infection as compared to the V3034 infection. Significant upregulation of VCAM-1 was also observed in V3000 infected mice brain, but not in V3034 infected mice brain. VCAM-1 is a regulator of T-cell mediated inflammatory response and has been shown to contribute adversely towards immunopathology during viral infections [46]. Earlier, we have shown an increased expression of VCAM-1 in VEEV infected mouse brain [9]. Although V3000 and V3034 both demonstrated inflamed vessels in the brain, gene network analysis found a much stronger activation of inflammatory response such as recruitment of lymphocytes, infiltration of granulocytes, movement of neutrophils and migration of leukocytes, NK cells and T-cells with V3000 infection as compared to V3034 infection in the mouse brain. Other studies have also demonstrated that infiltration of T cells and a strong NK cell response in the brain is associated with lethal alphavirus infections [47, 48]. Selective upregulation of the chemokines and adhesion molecules in V3000 infected mouse brains thereby, may be responsible for the higher degree of inflammation that was observed in V3000 infected brain as compared to the V3034 infected brain, and genes such as *Ccl2*, *Ccl6*, *Ly6* and *VCAM-1* may play role in adverse outcome of VEEV replication in brain.

## Conclusion

In conclusion, comparative analysis of gene expression from the spleen and brain of mice infected with highly neurovirulent V3000 or partially neurovirulent V3034 strains of VEEV identified several host factors that support the increased inflammatory reaction, spleen pathology and lethal encephalitis following V3000 infection. One of the limitations of this study is that the differences in the number of significantly modulated genes and their expression levels could be affected by the level of virus replication in the tissue. At the same time, the decreased pathology observed in the tissue of mice infected with less pathogenic virus could be a reflection of better control and repair by the host response against the virus replication. The goal of present study was to evaluate the molecular responses in the host tissues that are primarily targeted by VEEV. Overall responses discussed in this study are representative of the combined response of various cell types present in the lymphoid tissue (spleen) and the brain. Further studies will be needed to identify responses of individual cell types against the different strains of VEEV and the role of individual genes in supporting or controlling the VEEV infection and disease.

## Methods

### Virus strains and animals

Virus stocks used in this study were acquired from Dr. Franziska B. Grieder, USUHS, Bethesda, MD. Two different strains of VEEV were used: V3000 and V3034. Six-ten weeks old male CD-1 mice (n = 3 per group, 10-12 g, purchased from Jackson Laboratory, Bar Harbor, ME) were used for the studies. Mice were given mild anesthesia using isoflurane and 1000 pfu of either of the 2 strains of VEEV in 25 μl volume was inoculated in the left rear footpad using sterile 26G(3/8) intradermal bevel needle on 1cm^3^ sterile syringe (Becton Dickinson and Company, Franklin Lakes, NJ). Footpad inoculation was chosen as it closely mimics the natural route of VEEV infection via mosquito bite. Working dilutions of virus were made by diluting stock virus solution in 1X Dulbecco’s phosphate buffered saline (DPBS) (Gibco, Invitrogen Corporation, Carlsbad, CA). Control mice were inoculated with 25 μl of sterile 1X DPBS in left rear footpad. Spleen samples were collected at 24 h, 48 h and 72 h pi and brain samples were collected at 48 h, 72 h and 96 h pi and were immediately stored in trizol at −80 °C for RNA extraction or in formalin at room temperature for histological evaluation. The experiments with virulent VEEV were conducted in the BSL3 facility at USUHS during the time period when USUHS was registered with the CDC select agent program. All the animal handling and euthanasia protocols were reviewed and approved by the Institutional Animal Care and Use Committees.

### Total RNA isolation

Total RNA from spleen was collected at 24 h, 48 h and 72 h pi and from brain was collected at 48 h, 72 h and 96 h pi using TriZol method (Invitrogen Inc., Carlsbad, CA) according to the manufacturers’ protocols. Briefly, the tissue was minced in TriZol reagent (1X volume) and mixed with chloroform (0.2X volume). After 5 min of incubation, the aqueous and organic phases were separated by centrifugation at 11,000 rpm for 15 min at 4 °C. The aqueous phase was collected and mixed with isopropanol (0.5X volume) followed by centrifugation at 11,000 rpm for 10 min at 4C. The pellet was washed with 1 ml 70% ethanol and centrifuged at 9500 rpm for 5 min twice. RNA pellet was then air dried and dissolved in nuclease free water. The total RNA isolated was then purified using the RNeasy mini kit (Qiagen Inc.) to remove any contamination. Briefly, 100 μl RNA sample was mixed with 350 μl RLT buffer and 250 μl 100% ethanol. After vigorous mixing, it was filtered through the filter cartridge provided with the kit at 10,000 rpm for 30 s. Filter cartridge was washed twice by loading 500 μl RPE buffer and centrifugation at 10,000 rpm for 30 s. Filter cartridge was transferred onto a fresh tube and spin dried at 10,000 rpm for 1 min. RNA was then eluted in 30-50 μl nuclease free water. Purified RNA was quantified spectrophotometrically using Beckman DU640 spectrophotometer (Beckman Instruments Inc., Columbia, MD) and stored at −80 °C. RNA quality was determined by running it on 1% denaturing formaldehyde agarose gel.

### Microarray experiment

The high quality mouse microarrays containing oligonucleotides were produced in the laboratory at CBER/FDA. These arrays contain 35,852 oligonucleotide (70-mer) probes, representing approximately 25,000 unique genes and 38,000 transcripts, and additional 380 oligonucleotides as positive, negative and spike-in controls. The oligo set (version 4.0) used for these arrays was purchased from Operon Molecules for Life, Inc. The design of probes was based on the Ensembl Mouse Database release 26.33b.1, Mouse Genome Sequencing Project, NCBI RefSeq, Riken full-length cDNA clone sequences and other GenBank sequences. The detailed information regarding array printing, post-printing processing, and testing array quality is described elsewhere [49].

The total RNA isolated from brain and spleen samples was used for microarray hybridization and labeling using 3DNA Array 900 expression array detection kit from Genisphere Inc. as described by the manufacturer. Briefly, cDNA was synthesized from 1.5 μg of total RNA at 42 °C using SS-II reverse transcription enzyme and RT primers specific to cy5/cy3 dye dendrimers (Genisphere Inc.). The cDNAs were first hybridized with microarray slides overnight at 42 °C in MAUI microarray hybridization chambers (Biomicro systems Inc.) followed by stringent washes. Slides hybridized with cDNA were then end labeled with Cy5/Cy3 dyes containing dendrimers at 65 °C for 5 h in MAUI microarray hybridization chambers followed by stringent washes. The slides were scanned using the Axon GenePix 4000B scanner with a 10-μm resolution.

### Microarray data analysis

Microarray slides were scanned on an Axon GenePix 4000B scanner (Axon Instruments, Inc., Foster City, CA) with a 10-μm resolution. The raw data files were generated from scanned microarray images using GenePix Pro 5.1 software were imported into mAdb (microarray database), and analyzed by the software tools in the mAdb database provided by Center for Information Technology (CIT), National Institutes of Health (NIH) as described before [8]. The advanced filters were applied before data analysis to select only the spots with spot size of 10 μm to 300 μm, ≤80% signal saturation, minimum fluorescent intensity of 150 and signal ≥2SD (standard deviation) above background in both Cy3 and Cy5 channels. Global normalization was performed utilizing the Loess normalization method. All the biological replicates (n = 3 at each time point) shared significant homology in gene expression pattern with correlation coefficients of ≥0.80 and clustered together in hierarchical clustering analysis. The genes with ≥2.0 fold relative expression (log_2_ ratio ≥ 1.0 or ≤ −1.0) over the uninfected controls in all the three biological replicates and with *p*-value ≤0.05 were considered significantly modulated. Further analysis was performed using software tools in the Ingenuity Pathway Analysis (IPA) Software suit (https://www.qiagenbioinformatics.com/products/ingenuity-pathway-analysis/) to identify biological functions and relevant pathways representing these genes. Gene networks were also generated using the web-based tools from IPA.

### Histology

Spleen and brain samples collected at various time points pi were immediately fixed in 10% phosphate buffered formalin for 3–4 weeks. Paraffin embedded tissue was sectioned at 5 μm thickness and stained with haematoxylin and eosin (H & E) for histopathological analysis. For detecting the presence of virus, tissue sections were incubated with anti-VEEV antibody (rabbit polyclonal antiserum (1 : 10,000), raised against VEEV, eastern equine encephalitis virus, western equine encephalitis virus and sindbis virus; kindly provided by Dr. Cindy Rossi and Dr. George Ludwig, United States Army Medical Research Institute for Infectious Diseases, Frederick, MD) as previously described [8]. An indirect avidin-biotin-immunoperoxidase technique was used for IHC using Vectastain ABC Elite kit (Vector Laboratories) and sections were counter-stained with haematoxylin QS (Vector Laboratories) as specified by the manufacturer. Stained slides were examined under the microscope (Nikon Eclipse E400, Nikon, Instruments Inc., Melville, NY).

### Quantitative Real Time PCR

Expression level of selected genes from the microarray results was validated through quantitative real-time PCR. SuperScript- III First-Strand Synthesis System was used to prepare cDNA from amplified RNA samples and SYBR Green PCR master mix (Applied biosystems Inc.) was used to perform PCR. Reactions were run on 7900HT fast real time PCR machine using the following cycle: 95 °C for 10 min, followed by 40 cycles at 95 °C for 15 s and 60 °C for 1 min. The details of the primer sets used are given in Additional file 8: Table S7. The data obtained was analyzed with ABI RQ manager software (Carlsbad, CA) by the 2^-ΔΔCt^ method and normalized against housekeeping gene, GAPDH. Data is representative of at least 2 technical replicate for each biological replicate.

### Statistical analysis

All the results are expressed as mean Log_2_ fold expression ± SEM (N = 3 per group). Student’s t- test was used for comparison of groups. For all data, statistical significance was accepted at *p* ≤ 0.05.

## Additional files

Additional file 1: Figure S1. Venn diagram with comparison of gene expression at different time points in **a**) V3000 infected spleen, **b**) V3034 infected spleen, **c**) V3000 infected brain, and **d**) V3034 infected brain. Significantly modulated genes against each virus at different time points studied were compared in spleen and brain. The genes representing unique or common subsets are shown in the venn diagrams. The numbers in the venn diagrams include both upregulated and downregulated genes. **Figure S2.** Real-time PCR based validation. Real-time PCR analysis was performed to confirm the microarray results for randomly selected genes a) Stat1, b) Stat2, c) Zfp456, Nt5c2 and NfκB2 post V3000 infection and d) Samd9l post V3000 and V3034 infections. Expression values of all the genes were normalized with the house keeping gene, GAPDH. The results here are representative of 2 biological replicates and 2 technical replicates for each biological replicate. Blue bar: RT-PCR expression level; Red bar: Microarray expression level. Details of primer sets used are given in supplementary table-7. **Figure S3.** Network analysis of apoptotic genes modulated in response to V3000 and V3034 infections in spleen at 48 h and 72 h pi. Genes significantly modulated against V3000 and V3034 infections at 48 h and 72 h pi in spleen were used to perform in silico network analysis using the Ingenuity Pathway analysis software. Both the viruses resulted in modulation of apoptosis pathway at 48 h and 72 h pi. **Figure S4.** Network analysis of inflammatory genes modulated in response to V3000 and V3034 infections in spleen at 48 h and 72 h pi. Genes significantly modulated against V3000 and V3034 infections at 48 h and 72 h pi in spleen were used to perform in silico network analysis using the Ingenuity Pathway analysis software. Both the viruses resulted in activation of various immune cells to different degrees at 48 h and 72 h pi as shown above. (DOCX 1842 kb)

Additional file 2: Table S1. Significantly modulated genes common against V3000 and V3034 strains of VEEV in spleen. Genes that were modulated with both V3000 and V3034 infection in the spleen were identified. The list summarizes the commonly modulated genes for each time point studied. Values are expressed as average values of (log_2_) fold expression for each gene over uninfected controls ± standard error mean (SEM). * *P* ≤ 0.05. (DOCX 45 kb)

Additional file 3: Table S2. Significantly modulated genes common against V3000 and V3034 strains of VEEV in brain. Genes that were modulated with both V3000 and V3034 infection in the brain were identified. The list summarizes the commonly modulated genes for each time point studied. Values are expressed as average values of (log_2_) fold expression for each gene over uninfected controls ± standard error mean (SEM). * *P* ≤ 0.05. (DOCX 43 kb)

Additional file 4: Table S3. Significantly modulated genes in the spleen that were unique to V3000 infection. Genes that were modulated only with V3000 infection in the spleen were identified. The list summarizes the commonly modulated genes for each time point studied. Values are expressed as average values of (log_2_) fold expression for each gene over uninfected controls ± standard error mean (SEM). * *P* ≤ 0.05. (DOCX 82 kb)

Additional file 5: Table S4. Significantly modulated genes in the brain that were unique to V3000 infection. Genes that were modulated only with V3000 infection in the brain were identified. The list summarizes the commonly modulated genes for each time point studied. Values are expressed as average values of (log_2_) fold expression for each gene over uninfected controls ± standard error mean (SEM). * *P* ≤ 0.05. (DOCX 38 kb)

Additional file 6: Table S5: Significantly modulated genes in the spleen that were unique to V3034 infection. Genes that were modulated only with V3034 infection in the spleen were identified. The list summarizes the commonly modulated genes for each time point studied. Values are expressed as average values of (log_2_) fold expression for each gene over uninfected controls ± standard error mean (SEM). * *P* ≤ 0.05. (DOCX 50 kb)

Additional file 7: Table S6. Significantly modulated genes in the brain that were unique to V3034 infection. Genes that were modulated only with V3034 infection in the brain were identified. The list summarizes the commonly modulated genes for each time point studied. Values are expressed as average values of (log_2_) fold expression for each gene over uninfected controls ± standard error mean (SEM). * *P* ≤ 0.05. (DOCX 29 kb)

Additional file 8: Table S7. Primer sequences used for real-time PCR. Real-time PCR analysis was performed to confirm the microarray results for Stat1, Stat2, Zfp456, Nt5c2, NfκB2 and Samd9l post V3000 and V3034 infections at various time points. Expression values of all the genes were normalized with the house keeping gene, GAPDH. The details of primer sets used are summarized in this table. (DOCX 14 kb)

## Abbreviations

CCR5: C-C motif Chemokine Receptor5
CIT: Center for Information Technology
CNS: Central Nervous System
CXCR4: CXC chemokine receptor4
DC: Dendritic Cells
DPBS: Dulbecco’s Phosphate Buffered Saline
EMH: Extra Medullary Hematopoiesis
GAPDH: Glyceraldehyde-3-Phosphate Dehydrogenase
H&E: Hematoxylin and Eosin
H2-T23: Histocompatibility 2, T region locus 23
IL: Interleukins
IPA: Ingenuity Pathway Analysis
IRF: Interferon Regulatory Factor
NIH: National Institutes of Health
NKC: Natural Killer Cells
Pi: Post Infection
PRR: Pattern Recognition Receptors
TCR: T-cell Receptor
TLR: Toll Like Receptors
TNFR1: Tumor Necrosis Factor Receptor1
TSA-1: Thymic Shared Antigen-1
VCAM: Vascular Cell Adhesion protein
VEEV: Venezuelan Equine Encephalitis Virus
