# Supplementary material for: Differential host gene responses from infection with neurovirulent and partially-neurovirulent strains of Venezuelan equine encephalitis virus
Source: BMC Infect Dis. 2017 Apr 26;17:309. doi: 10.1186/s12879-017-2355-3 (PMC5405508; doi:10.1186/s12879-017-2355-3)
Supplement: Supplementary file 1 — Venn diagram with comparison of gene expression at different time points in a) V3000 infected spleen, b) V3034 infected spleen, c) V3000 infected brain, and d) V3034 infected brain. Significantly modulated genes against each virus at different time points studied were compared in spleen and brain. The genes representing unique or common subsets are shown in the venn diagrams. The numbers in the venn diagrams include both upregulated and downregulated genes. Figure S2. Real-time PCR based validation. Real-time PCR analysis was performed to confirm the microarray results for randomly selected genes a) Stat1, b) Stat2, c) Zfp456, Nt5c2 and NfκB2 post V3000 infection and d) Samd9l post V3000 and V3034 infections. Expression values of all the genes were normalized with the house keeping gene, GAPDH. The results here are representative of 2 biological replicates and 2 technical replicates for each biological replicate. Blue bar: RT-PCR expression level; Red bar: Microarray expression level. Details of primer sets used are given in supplementary table-7. Figure S3. Network analysis of apoptotic genes modulated in response to V3000 and V3034 infections in spleen at 48 h and 72 h pi. Genes significantly modulated against V3000 and V3034 infections at 48 h and 72 h pi in spleen were used to perform in silico network analysis using the Ingenuity Pathway analysis software. Both the viruses resulted in modulation of apoptosis pathway at 48 h and 72 h pi. Figure S4. Network analysis of inflammatory genes modulated in response to V3000 and V3034 infections in spleen at 48 h and 72 h pi. Genes significantly modulated against V3000 and V3034 infections at 48 h and 72 h pi in spleen were used to perform in silico network analysis using the Ingenuity Pathway analysis software. Both the viruses resulted in activation of various immune cells to different degrees at 48 h and 72 h pi as shown above. (DOCX 1842 kb) [file 12879_2017_2355_MOESM1_ESM.docx]

**Additional figure 1**

**Fig. S1:**

**
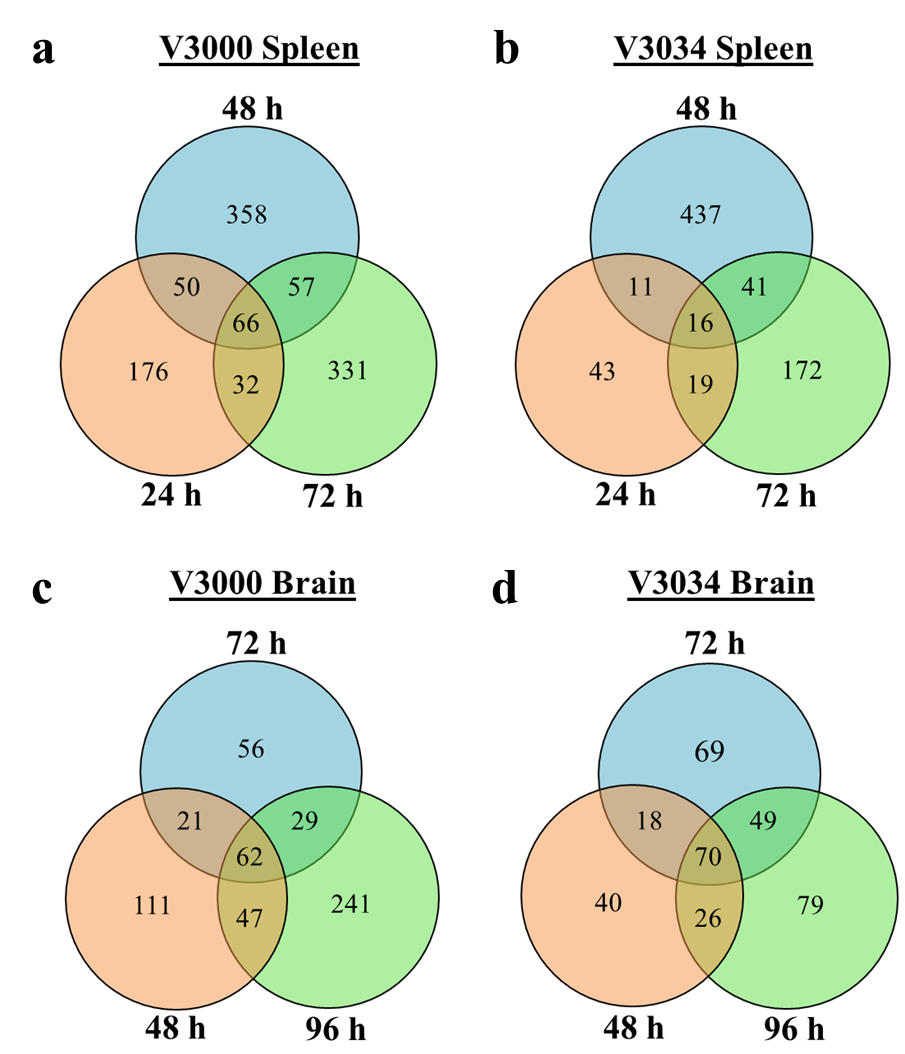
**

**Fig. S2:**


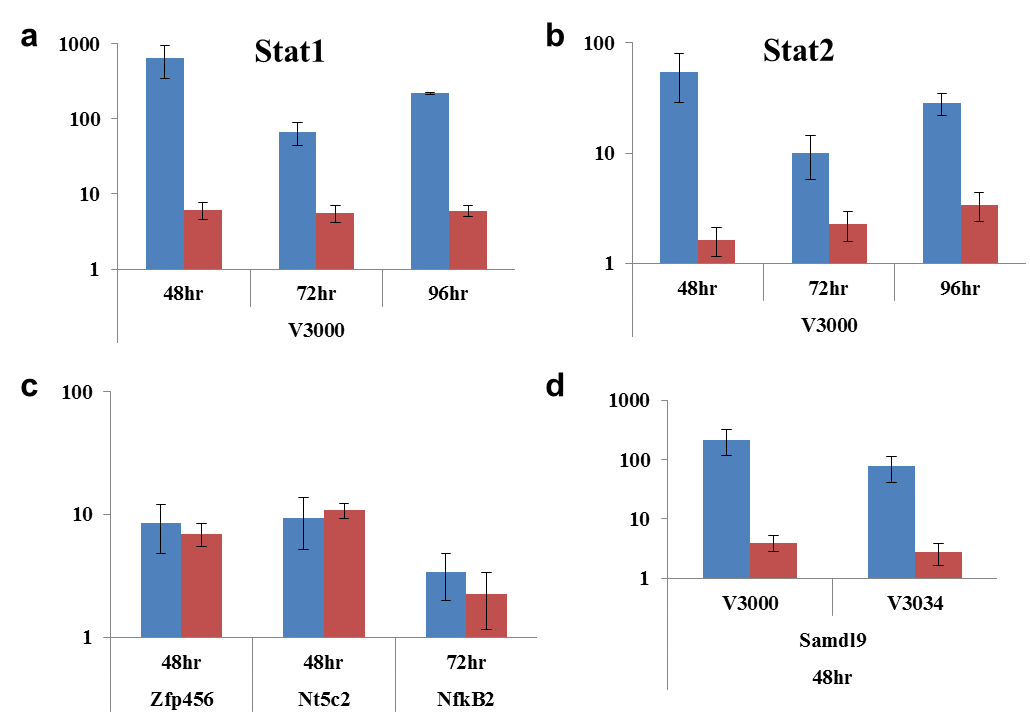


**Fig. S3:**
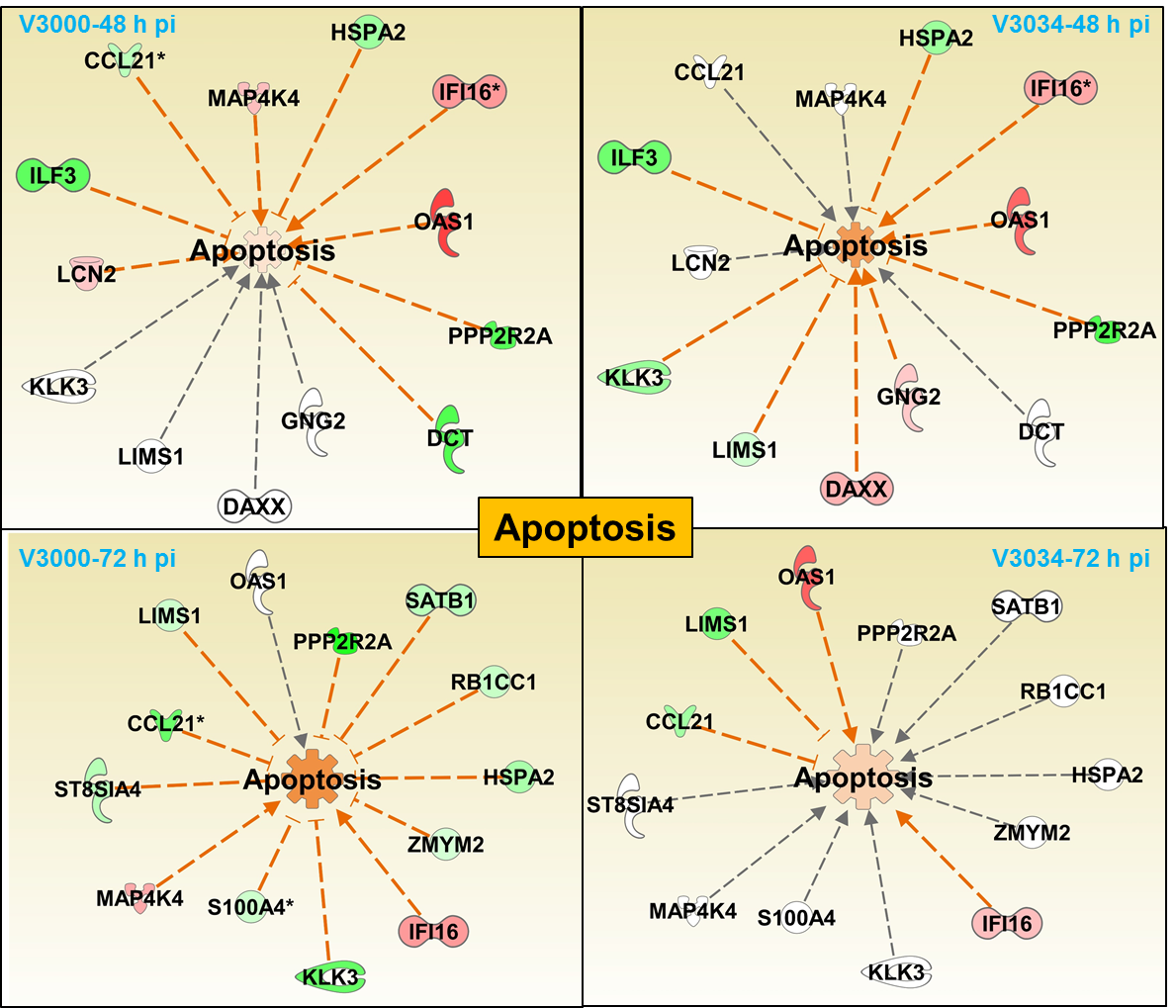


Legends: : inducer, : repressor,: leads to activation, : effect not predicted; dotted line represents indirect interaction between the molecules, : upregulated, : downregulated, : not expressed; intensity of the color is directly proportional to the level of modulation

**Fig. S4:**
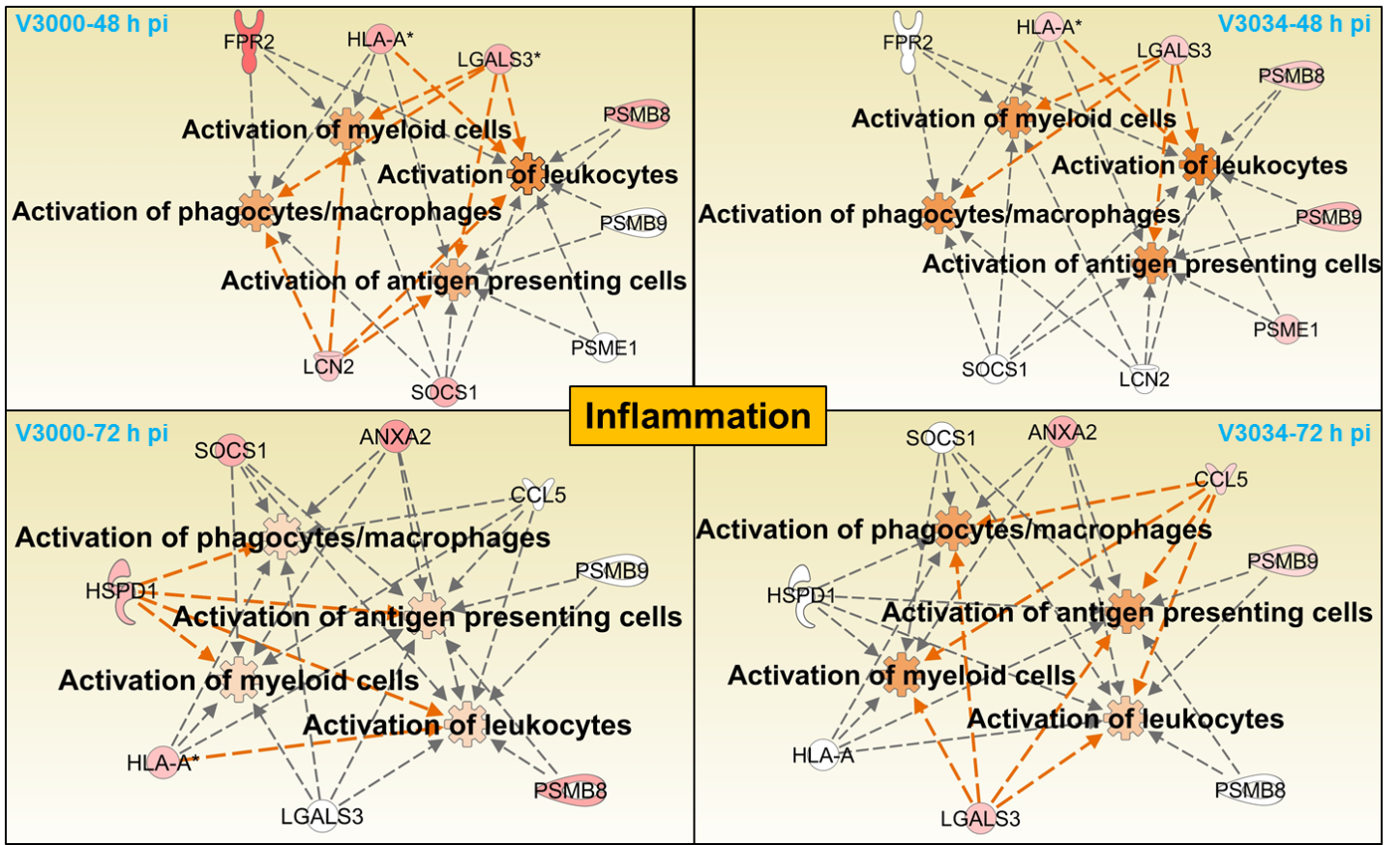


Legends: : inducer, : leads to activation, : effect not predicted; dotted line represents indirect interaction between the molecules, : upregulated, : not expressed; intensity of the color is directly proportional to the level of modulation.
