# Supplementary material for: Differential host gene responses from infection with neurovirulent and partially-neurovirulent strains of Venezuelan equine encephalitis virus
Source: BMC Infect Dis. 2017 Apr 26;17:309. doi: 10.1186/s12879-017-2355-3 (PMC5405508; doi:10.1186/s12879-017-2355-3)
Supplement: Supplementary file 2 — Significantly modulated genes common against V3000 and V3034 strains of VEEV in spleen. Genes that were modulated with both V3000 and V3034 infection in the spleen were identified. The list summarizes the commonly modulated genes for each time point studied. Values are expressed as average values of (log2) fold expression for each gene over uninfected controls ± standard error mean (SEM). * P ≤ 0.05. (DOCX 45 kb) [file 12879_2017_2355_MOESM2_ESM.docx]

**Additional file 2:Table S1: Significantly modulated genes common against V3000 and V3034 strains of VEEV in spleen**

| **UniGene** | **Gene** | **Description** | **Log_2_ Exp ± SEM** | |
| --- | --- | --- | --- | --- |
|  |  |  | **V3000** | **V3034** |
| **Genes common at 24 h pi** | | | | |
| Mm.40965 | Nt5c2 | 5'-nucleotidase, cytosolic II | **4.60 ± 0.28** | **3.99 ± 0.28** |
| Mm.8369 | Mst1 | Macrophage stimulating 1 | **3.90 ± 0.55** | **4.34 ± 0.43** |
| Mm.116687 | Zbp1 | Z-DNA binding protein 1 | **3.58 ± 0.35** | **2.98 ± 0.37** |
| Mm.141021 | Ifitm3 | Interferon induced transmembrane protein 3 | **3.38 ± 0.50** | **3.29 ± 0.50** |
| Mm.485478 | Trim30b | Tripartite motif-containing 30B | **2.80 ± 0.34** | **3.37 ± 0.65** |
| Mm.271275 | Ifi27l2a | Interferon, alpha-inducible protein 27 like 2A | **2.64 ± 0.14** | **3.15 ± 0.34** |
| Mm.34609 | Plac8 | Placenta-specific 8 | **2.57 ± 0.48** | **1.75 ± 0.39** |
| Mm.440715 | Ms4a4c | Membrane-spanning 4-domains, subfamily A, member 4C | **2.56 ± 0.27** | **1.97 ± 0.29** |
| Mm.269029 | Slc7a6os | Solute carrier family 7, member 6 opposite strand | **2.15 ± 0.08** | **2.17 ± 0.10** |
| Mm.17932 | Pnp | Purine-nucleoside phosphorylase | **1.94 ± 0.19** | **1.99 ± 0.23** |
| Mm.426537 | LOC100041885 | PREDICTED: sp110 nuclear body protein-like | **1.57 ± 0.33** | **1.71 ± 0.27** |
| Mm.277125 | Uba7 | Ubiquitin-like modifier activating enzyme 7 | **1.54 ± 0.28** | **1.57 ± 0.05** |
| Mm.21874 | Psmb3 | Proteasome (prosome, macropain) subunit, beta type 3 | **1.53 ± 0.11** | **2.01 ± 0.20** |
| Mm.311516 | Olfr124 | Olfactory receptor 124 | **1.50 ± 0.12** | **1.75 ± 0.33** |
| Mm.30049 | C1qbp | Complement component 1, q subcomponent binding protein | **1.46 ± 0.27** | **1.27 ± 0.05** |
| Mm.341719 | Wdr89 | WD repeat domain 89 | **1.43 ± 0.29** | **1.87 ± 0.07** |
| Mm.2948 | H2-Ke2 | H2-K region expressed gene 2 | **1.37 ± 0.16** | **1.73 ± 0.28** |
| Mm.288697 | Slc25a27 | Solute carrier family 25, member 27 | **1.28 ± 0.13** | **1.82 ± 0.11** |
| Mm.2639 | Ly86 | Lymphocyte antigen 86 | **1.06 ± 0.15** | **1.17 ± 0.13** |
| Mm.2326 | Mif | Macrophage migration inhibitory factor | **1.04 ± 0.15** | **1.12 ± 0.24** |
| Mm.1360 | Gadd45b | Growth arrest and DNA-damage-inducible 45 beta | **1.03 ± 0.21** | **1.09 ± 0.23** |
| Mm.1461 | Sell | Selectin, lymphocyte | **1.02 ± 0.22** | **1.19 ± 0.18** |
| Mm.110220 | Ddit3 | DNA-damage inducible transcript 3 | **1.01 ± 0.20** | **1.73 ± 0.38** |
| Mm.150401 | Stk32a | Serine/threonine kinase 32A | **-1.23 ± 0.28** | **-1.11 ± 0.05** |
| Mm.3181 | Arhgef1 | Rho guanine nucleotide exchange factor (GEF) 1 | **-1.33 ± 0.25** | **-1.19 ± 0.23** |
| Mm.377071 | Mrps33 | Mitochondrial ribosomal protein S33 | **-1.70 ± 0.18** | **-1.29 ± 0.10** |
| Mm.3951 | Thy1 | Thymus cell antigen 1, theta | **-1.96 ± 0.22** | **-2.33 ± 0.31** |
| Mm.111904 | Srpk3 | Serine/arginine-rich protein specific kinase 3 | **-1.97 ± 0.26** | **-1.09 ± 0.13** |
| Mm.170905 | Fyb | FYN binding protein | **-2.13 ± 0.20** | **-2.01 ± 0.42** |
| Mm.227117 | Slc30a10 | Solute carrier family 30, member 10 | **-2.47 ± 0.34** | **-2.10 ± 0.28** |
| Mm.3468 | Socs3 | Suppressor of cytokine signaling 3 | **-2.88 ± 0.49** | **-3.45 ± 0.56** |
| Mm.425599 | Lamb2 | Laminin, beta 2 | **-2.97 ± 0.66** | **-1.75 ± 0.23** |
| **Genes common at 48 h pi** | | | | |
| Mm.40965 | Nt5c2 | 5'-nucleotidase, cytosolic II | **4.61 ± 0.50** | **4.84 ± 0.45** |
| Mm.461583 | Zfp456 | Zinc finger protein 456 | **3.68 ± 0.48** | **3.94 ± 0.62** |
| Mm.482110 | Ly6c2 | Lymphocyte antigen 6 complex, locus C2 | **3.61 ± 0.51** | **3.2 ± 0.01** |
| Mm.389688 | Oas1g | 2'-5' oligoadenylate synthetase 1G | **3.58 ± 0.35** | **3.58 ± 0.39** |
| Mm.377095 | Ly6f | Lymphocyte antigen 6 complex, locus F | **3.46 ± 0.51** | **3.19 ± 0.19** |
| Mm.39825 | Lmx1b | LIM homeobox transcription factor 1 beta | **3.19 ± 0.34** | **3.62 ± 0.80** |
| Mm.425949 | Ly6a | Lymphocyte antigen 6 complex, locus A | **3.13 ± 0.42** | **2.92 ± 0.24** |
| Mm.271275 | Ifi27l2a | Interferon, alpha-inducible protein 27 like 2A | **3.01 ± 0.25** | **3.17 ± 0.31** |
| Mm.260325 | Bst2 | Bone marrow stromal cell antigen 2 | **2.92 ± 0.29** | **2.85 ± 0.37** |
| Mm.40655 | Fam185a | Family with sequence similarity 185, member A | **2.89 ± 0.63** | **1.74 ± 0.16** |
| Mm.4950 | Isg15 | ISG15 ubiquitin-like modifier | **2.88 ± 0.39** | **2.66 ± 0.09** |
| Mm.245154 | Tmeff2 | Transmembrane protein with EGF-like and two follistatin-like domains 2 | **2.86 ± 0.22** | **3.03 ± 0.34** |
| Mm.20079 | Calml3 | Calmodulin-like 3 | **2.82 ± 0.34** | **3.34 ± 0.57** |
| Mm.377828 | Olfr547 | Olfactory receptor 547 | **2.78 ± 0.19** | **1.87 ± 0.32** |
| Mm.116687 | Zbp1 | Z-DNA binding protein 1 | **2.77 ± 0.10** | **2.49 ± 0.28** |
| Mm.485478 | Trim30b | Tripartite motif-containing 30B | **2.68 ± 0.23** | **3.26 ± 0.65** |
| Mm.218770 | Ifi202b | Interferon activated gene 202B | **2.68 ± 0.61** | **3.61 ± 0.26** |
| Mm.7454 | Igbp1 | Immunoglobulin (CD79A) binding protein 1 | **2.68 ± 0.19** | **2.68 ± 0.24** |
| Mm.6676 | Cd5l | CD5 antigen-like | **2.65 ± 0.25** | **2.76 ± 0.24** |
| Mm.188108 | Gm1574 | Predicted gene 1574 | **2.49 ± 0.08** | **1.92 ± 0.02** |
| Mm.323595 | Tob2 | Transducer of ERBB2, 2 | **2.40 ± 0.29** | **2.58 ± 0.49** |
| Mm.24769 | Ifi47 | Interferon gamma inducible protein 47 | **2.31 ± 0.14** | **2.54 ± 0.18** |
| Mm.20440 | Htr4 | 5 hydroxytryptamine (serotonin) receptor 4 | **2.23 ± 0.16** | **2.30 ± 0.09** |
| Mm.247736 | Gm4902 | Predicted gene 4902 | **2.19 ± 0.22** | **2.31 ± 0.24** |
| Mm.358668 | Prpf40b | PRP40 pre-mRNA processing factor 40 homolog B | **2.14 ± 0.11** | **2.01 ± 0.12** |
| Mm.256414 | Slc9a2 | Solute carrier family 9 (sodium/hydrogen exchanger), member 2 | **2.13 ± 0.13** | **2.43 ± 0.12** |
| Mm.30435 | Tsc2 | Tuberous sclerosis 2 | **2.11 ± 0.14** | **1.87 ± 0.21** |
| Mm.223180 | Olfr1195 | Olfactory receptor 1195 | **2.09 ± 0.39** | **2.54 ± 0.28** |
| Mm.269029 | Slc7a6os | Solute carrier family 7, member 6 opposite strand | **2.09 ± 0.11** | **2.16 ± 0.22** |
| Mm.259916 | Chdh | Choline dehydrogenase | **2.05 ± 0.05** | **2.01 ± 0.31** |
| Mm.33443 | Zfp677 | Zinc finger protein 677 | **2.03 ± 0.19** | **2.68 ± 0.38** |
| Mm.248615 | Lgals3 | Lectin, galactose binding, soluble 3 | **2.01 ± 0.20** | **1.18 ± 0.18** |
| Mm.30466 | Trps1 | Trichorhinophalangeal syndrome I | **1.95 ± 0.37** | **1.62 ± 0.06** |
| Mm.261270 | Ifi204 | Interferon activated gene 204 | **1.93 ± 0.10** | **2.32 ± 0.32** |
| Mm.211477 | Phldb2 | Pleckstrin homology-like domain, family B, member 2 | **1.91 ± 0.40** | **2.67 ± 0.33** |
| Mm.32881 | Spnb1 | Spectrin beta 1 | **1.88 ± 0.28** | **2.05 ± 0.35** |
| Mm.278689 | Slfn2 | Schlafen 2 | **1.78 ± 0.07** | **1.72 ± 0.07** |
| Mm.24038 | Gbp2 | Guanylate binding protein 2 | **1.76 ± 0.13** | **2.65 ± 0.17** |
| Mm.110220 | Ddit3 | DNA-damage inducible transcript 3 | **1.75 ± 0.35** | **1.66 ± 0.31** |
| Mm.431282 | H2-Q9 | Histocompatibility 2, Q region locus 9 | **1.74 ± 0.26** | **1.38 ± 0.23** |
| Mm.33902 | Igtp | Interferon gamma induced GTPase | **1.73 ± 0.09** | **1.58 ± 0.28** |
| Mm.17185 | Lgmn | Legumain | **1.71 ± 0.37** | **1.59 ± 0.12** |
| Mm.482724 | Gm13102 | Predicted gene 13102 | **1.65 ± 0.28** | **1.48 ± 0.10** |
| Mm.180191 | Psmb8 | Proteasome (prosome, macropain) subunit, beta type 8 | **1.63 ± 0.24** | **1.26 ± 0.19** |
| Mm.458517 | Irgm2 | Immunity-related GTPase family M member 2 | **1.59 ± 0.08** | **1.07 ± 0.15** |
| Mm.9901 | Nucb2 | Nucleobindin 2 | **1.57 ± 0.10** | **1.51 ± 0.21** |
| Mm.28212 | Sepx1 | Selenoprotein X 1 | **1.55 ± 0.24** | **1.41 ± 0.11** |
| Mm.8739 | Sgce | Sarcoglycan, epsilon | **1.54 ± 0.20** | **1.29 ± 0.05** |
| Mm.391959 | 1500012F01Rik | RIKEN cDNA 1500012F01 gene | **1.54 ± 0.35** | **1.80 ± 0.23** |
| Mm.216549 | Ankle1 | Ankyrin repeat and LEM domain containing 1 | **1.50 ± 0.33** | **1.53 ± 0.15** |
| Mm.259021 | Fxr1 | Fragile X mental retardation gene 1, autosomal homolog | **1.48 ± 0.23** | **1.25 ± 0.04** |
| Mm.89991 | Psg18 | Pregnancy specific glycoprotein 18 | **1.46 ± 0.18** | **1.79 ± 0.12** |
| Mm.291358 | Gm5218 | PREDICTED: predicted gene 5218 | **1.44 ± 0.24** | **1.01 ± 0.10** |
| Mm.442861 | H2-D4 | Histocompatibility 2, D region locus 4 | **1.44 ± 0.29** | **1.41 ± 0.24** |
| Mm.6095 | Cstb | Cystatin B | **1.43 ± 0.19** | **1.76 ± 0.26** |
| Mm.439957 | C1qa | Complement component 1, q subcomponent, alpha polypeptide | **1.36 ± 0.18** | **1.39 ± 0.28** |
| Mm.439732 | C1qc | Complement component 1, q subcomponent, C chain | **1.35 ± 0.09** | **1.40 ± 0.15** |
| Mm.27925 | Dbndd1 | Dysbindin (dystrobrevin binding protein 1) domain containing 1 | **1.32 ± 0.24** | **1.16 ± 0.12** |
| Mm.422814 | Serpina3c | Serine (or cysteine) peptidase inhibitor, clade A, member 3C | **1.32 ± 0.16** | **1.27 ± 0.24** |
| Mm.237594 | Ufd1l | Ubiquitin fusion degradation 1 like | **1.31 ± 0.13** | **1.29 ± 0.23** |
| Mm.26135 | Cab39 | Calcium binding protein 39 | **1.31 ± 0.22** | **1.19 ± 0.21** |
| Mm.283137 | Chrna6 | Cholinergic receptor, nicotinic, alpha polypeptide 6 | **1.30 ± 0.13** | **1.64 ± 0.14** |
| Mm.236795 | Mrpl13 | Mitochondrial ribosomal protein L13 | **1.29 ± 0.28** | **1.19 ± 0.10** |
| Mm.326477 | Zfp445 | Zinc finger protein 445 | **1.26 ± 0.10** | **1.01 ± 0.22** |
| Mm.38888 | Serping1 | Serine (or cysteine) peptidase inhibitor, clade G, member 1 | **1.22 ± 0.22** | **1.00 ± 0.04** |
| Mm.479273 | Nphp3 | Nephronophthisis 3 (adolescent) | **1.21 ± 0.06** | **1.32 ± 0.04** |
| Mm.24399 | Klra15 | Killer cell lectin-like receptor, subfamily A, member 15 | **1.21 ± 0.24** | **2.23 ± 0.21** |
| Mm.440715 | Ms4a4c | Membrane-spanning 4-domains, subfamily A, member 4C | **1.21 ± 0.07** | **2.11 ± 0.36** |
| Mm.485399 | Atp6v1g1 | ATPase, H+ transporting, lysosomal V1 subunit G1 | **1.19 ± 0.16** | **1.30 ± 0.16** |
| Mm.28162 | Nup210 | Nucleoporin 210 | **1.18 ± 0.12** | **1.74 ± 0.21** |
| Mm.13944 | Rps9 | Ribosomal protein S9 | **1.14 ± 0.10** | **1.21 ± 0.21** |
| Mm.302791 | Ncln | Nicalin homolog | **1.14 ± 0.14** | **1.06 ± 0.04** |
| Mm.21874 | Psmb3 | Proteasome (prosome, macropain) subunit, beta type 3 | **1.12 ± 0.14** | **1.37 ± 0.05** |
| Mm.249555 | Col3a1 | Collagen, type III, alpha 1 | **1.07 ± 0.04** | **1.40 ± 0.26** |
| Mm.2032 | Irf9 | Interferon regulatory factor 9 | **1.06 ± 0.02** | **1.31 ± 0.23** |
| Mm.41569 | Fam176b | Family with sequence similarity 176, member B | **1.05 ± 0.20** | **1.44 ± 0.11** |
| Mm.275800 | Slc12a7 | Solute carrier family 12, member 7 | **1.03 ± 0.23** | **1.10 ± 0.08** |
| Mm.348794 | Aym1 | Activator of yeast meiotic promoters 1 | **-1.03 ± 0.19** | **-1.23 ± 0.08** |
| Mm.215173 | Ptcra | Pre T-cell antigen receptor alpha | **-1.05 ± 0.20** | **-1.28 ± 0.17** |
| Mm.119274 | Prrxl1 | Paired related homeobox protein-like 1 | **-1.09 ± 0.15** | **-1.45 ± 0.05** |
| Mm.345095 | Col20a1 | Collagen, type XX, alpha 1 | **-1.11 ± 0.09** | **-1.17 ± 0.17** |
| Mm.4716 | Htr1a | 5-hydroxytryptamine (serotonin) receptor 1A | **-1.11 ± 0.06** | **-1.38 ± 0.23** |
| Mm.441499 | 1700019L03Rik | RIKEN cDNA 1700019L03 gene | **-1.12 ± 0.07** | **-1.40 ± 0.10** |
| Mm.269088 | Anp32a | Acidic (leucine-rich) nuclear phosphoprotein 32 family, member A | **-1.12 ± 0.21** | **-1.03 ± 0.21** |
| Mm.223420 | Olfr711 | Olfactory receptor 711 | **-1.15 ± 0.06** | **-1.36 ± 0.19** |
| Mm.206238 | Med13l | Mediator complex subunit 13-like | **-1.16 ± 0.08** | **-1.00 ± 0.16** |
| Mm.371552 | Cd63 | CD63 antigen | **-1.18 ± 0.09** | **-1.76 ± 0.28** |
| Mm.439850 | Cdadc1 | Cytidine and dcmp deaminase domain containing 1 | **-1.20 ± 0.21** | **-1.21 ± 0.15** |
| Mm.391556 | Madcam1 | Mucosal vascular addressin cell adhesion molecule 1 | **-1.21 ± 0.14** | **-1.69 ± 0.04** |
| Mm.24783 | Frmpd1 | FERM and PDZ domain containing 1 | **-1.22 ± 0.18** | **-1.08 ± 0.14** |
| Mm.485067 | 4930481A15Rik | RIKEN cDNA 4930481A15 gene | **-1.22 ± 0.08** | **-1.44 ± 0.18** |
| Mm.34885 | Hspb6 | Heat shock protein, alpha-crystallin-related, B6 | **-1.23 ± 0.18** | **-1.44 ± 0.11** |
| Mm.289796 | Lins | Lines homolog | **-1.26 ± 0.23** | **-1.70 ± 0.16** |
| Mm.37426 | Cd163 | CD163 antigen | **-1.29 ± 0.13** | **-1.32 ± 0.11** |
| Mm.331191 | Sprr1a | Small proline-rich protein 1A | **-1.31 ± 0.20** | **-1.55 ± 0.20** |
| Mm.110594 | Rgl3 | Ral guanine nucleotide dissociation stimulator-like 3 | **-1.34 ± 0.30** | **-1.42 ± 0.23** |
| Mm.324601 | Olfr282 | Olfactory receptor 282 | **-1.35 ± 0.11** | **-1.49 ± 0.20** |
| Mm.262270 | Syt12 | Synaptotagmin XII | **-1.37 ± 0.10** | **-1.11 ± 0.23** |
| Mm.23782 | Glt25d2 | Glycosyltransferase 25 domain containing 2 | **-1.38 ± 0.30** | **-1.56 ± 0.31** |
| Mm.260137 | Slc2a9 | Solute carrier family 2 (facilitated glucose transporter), member 9 | **-1.38 ± 0.27** | **-1.16 ± 0.23** |
| Mm.439970 | Cox16 | COX16 cytochrome c oxidase assembly homolog | **-1.39 ± 0.25** | **-1.57 ± 0.22** |
| Mm.235891 | Eri3 | Exoribonuclease 3 | **-1.40 ± 0.26** | **-1.27 ± 0.02** |
| Mm.234152 | Asb1 | Ankyrin repeat and SOCS box-containing 1 | **-1.41 ± 0.17** | **-1.59 ± 0.34** |
| Mm.183576 | 5330437I02Rik | RIKEN cDNA 5330437I02 gene | **-1.43 ± 0.26** | **-1.74 ± 0.11** |
| Mm.29854 | Hdac6 | Histone deacetylase 6 | **-1.43 ± 0.16** | **-1.28 ± 0.07** |
| Mm.4183 | Plau | Plasminogen activator, urokinase | **-1.44 ± 0.25** | **-1.35 ± 0.29** |
| Mm.244068 | Arhgef7 | Rho guanine nucleotide exchange factor (GEF7) | **-1.45 ± 0.14** | **-2.33 ± 0.12** |
| Mm.403943 | E130304F04Rik | RIKEN cDNA E130304F04 gene | **-1.46 ± 0.33** | **-1.64 ± 0.35** |
| Mm.319117 | Ptpn12 | Protein tyrosine phosphatase, non-receptor type 12 | **-1.46 ± 0.03** | **-1.51 ± 0.19** |
| Mm.26908 | Csnk1a1 | Casein kinase 1, alpha 1 | **-1.47 ± 0.15** | **-1.63 ± 0.16** |
| Mm.393248 | Qk | Quaking | **-1.49 ± 0.29** | **-1.65 ± 0.08** |
| Mm.29497 | Iscu | Iscu iron-sulfur cluster scaffold homolog | **-1.53 ± 0.01** | **-1.32 ± 0.26** |
| Mm.1635 | Pias3 | Protein inhibitor of activated STAT 3 | **-1.53 ± 0.20** | **-1.23 ± 0.26** |
| Mm.34608 | Clybl | Citrate lyase beta like | **-1.53 ± 0.31** | **-1.59 ± 0.11** |
| Mm.331269 | Nr1h5 | Nuclear receptor subfamily 1, group H, member 5 | **-1.54 ± 0.22** | **-1.46 ± 0.21** |
| Mm.140601 | Cdc42ep3 | CDC42 effector protein (Rho GTPase binding) 3 | **-1.56 ± 0.04** | **-1.00 ± 0.22** |
| Mm.483321 | Bmp10 | Bone morphogenetic protein 10 | **-1.58 ± 0.11** | **-1.69 ± 0.32** |
| Mm.374865 | March2 | Membrane-associated ring finger (C3HC4) 2 | **-1.58 ± 0.18** | **-1.35 ± 0.21** |
| Mm.223639 | Pla2g4c | Phospholipase A2, group IVC (cytosolic, calcium-independent) | **-1.59 ± 0.17** | **-1.91 ± 0.10** |
| Mm.390829 | Mtbp | Mdm2, transformed 3T3 cell double minute p53 binding protein | **-1.61 ± 0.19** | **-1.54 ± 0.12** |
| Mm.439929 | 2810001A02Rik | PREDICTED: RIKEN cDNA 2810001A02 gene | **-1.62 ± 0.15** | **-1.70 ± 0.35** |
| Mm.2171 | Atpif1 | ATPase inhibitory factor 1 | **-1.62 ± 0.15** | **-1.22 ± 0.23** |
| Mm.272629 | Usp8 | Ubiquitin specific peptidase 8 | **-1.64 ± 0.23** | **-1.55 ± 0.15** |
| Mm.28474 | Rmnd5a | Required for meiotic nuclear division 5 homolog A | **-1.65 ± 0.28** | **-1.30 ± 0.10** |
| Mm.27435 | Slc22a17 | Solute carrier family 22 (organic cation transporter), member 17 | **-1.67 ± 0.29** | **-1.19 ± 0.25** |
| Mm.327439 | AA792892 | Expressed sequence AA792892 | **-1.70 ± 0.01** | **-1.17 ± 0.23** |
| Mm.29586 | Basp1 | Brain abundant, membrane attached signal protein 1 | **-1.71 ± 0.36** | **-1.14 ± 0.25** |
| Mm.268896 | Erich1 | Glutamate-rich 1 | **-1.72 ± 0.14** | **-1.37 ± 0.04** |
| Mm.391446 | Olfr794 | Olfactory receptor 794 | **-1.75 ± 0.15** | **-1.63 ± 0.01** |
| Mm.247775 | Hook2 | Hook homolog 2 | **-1.76 ± 0.19** | **-2.39 ± 0.02** |
| Mm.170905 | Fyb | FYN binding protein | **-1.79 ± 0.12** | **-1.56 ± 0.16** |
| Mm.16745 | Oxt | Oxytocin | **-1.82 ± 0.23** | **-1.65 ± 0.04** |
| Mm.195010 | Fat3 | FAT tumor suppressor homolog 3 | **-1.85 ± 0.36** | **-1.64 ± 0.26** |
| Mm.478808 | Zfp804b | Zinc finger protein 804B | **-1.86 ± 0.40** | **-1.85 ± 0.14** |
| Mm.27539 | Trappc10 | Trafficking protein particle complex 10 | **-1.86 ± 0.32** | **-1.92 ± 0.06** |
| Mm.1104 | Uba1 | Ubiquitin-like modifier activating enzyme 1 | **-1.89 ± 0.07** | **-2.47 ± 0.20** |
| Mm.52711 | 0610030E20Rik | RIKEN cDNA 0610030E20 gene | **-1.90 ± 0.12** | **-2.06 ± 0.22** |
| Mm.275138 | Dzip3 | DAZ interacting protein 3, zinc finger | **-1.92 ± 0.24** | **-2.00 ± 0.03** |
| Mm.287157 | Cinp | Cyclin-dependent kinase 2 interacting protein | **-1.94 ± 0.22** | **-1.77 ± 0.14** |
| Mm.295954 | Jmjd4 | Jumonji domain containing 4 | **-1.94 ± 0.05** | **-1.94 ± 0.16** |
| Mm.3468 | Socs3 | Suppressor of cytokine signaling 3 | **-1.95 ± 0.18** | **-2.72 ± 0.10** |
| Mm.25794 | Fbxl5 | F-box and leucine-rich repeat protein 5 | **-1.98 ± 0.42** | **-1.95 ± 0.23** |
| Mm.234965 | Foxp1 | Forkhead box P1 | **-1.99 ± 0.41** | **-1.28 ± 0.25** |
| Mm.213651 | Gk5 | Glycerol kinase 5 | **-1.99 ± 0.04** | **-2.39 ± 0.11** |
| Mm.285075 | Oprl1 | Opioid receptor-like 1 | **-2.01 ± 0.38** | **-1.78 ± 0.26** |
| Mm.252145 | Ckmt1 | Creatine kinase, mitochondrial 1, ubiquitous | **-2.03 ± 0.31** | **-2.25 ± 0.16** |
| Mm.138792 | Chd7 | Chromodomain helicase DNA binding protein 7 | **-2.07 ± 0.05** | **-2.01 ± 0.29** |
| Mm.217354 | Mrps6 | Mitochondrial ribosomal protein S6 | **-2.08 ± 0.25** | **-2.35 ± 0.24** |
| Mm.390986 | Dhx16 | DEAH (Asp-Glu-Ala-His) box polypeptide 16 | **-2.09 ± 0.30** | **-2.02 ± 0.26** |
| Mm.227117 | Slc30a10 | Solute carrier family 30, member 10 | **-2.11 ± 0.15** | **-1.88 ± 0.12** |
| Mm.377901 | Olfr670 | Olfactory receptor 670 | **-2.12 ± 0.32** | **-2.27 ± 0.18** |
| Mm.279485 | Rnh1 | Ribonuclease/angiogenin inhibitor 1 | **-2.17 ± 0.29** | **-2.30 ± 0.16** |
| Mm.73234 | Cep72 | Centrosomal protein 72 | **-2.18 ± 0.27** | **-2.33 ± 0.14** |
| Mm.246550 | Pou4f1 | POU domain, class 4, transcription factor 1 | **-2.21 ± 0.36** | **-2.12 ± 0.08** |
| Mm.296181 | Hspa2 | Heat shock protein 2 | **-2.22 ± 0.37** | **-2.51 ± 0.37** |
| Mm.217004 | Pigv | Phosphatidylinositol glycan anchor biosynthesis, class V | **-2.25 ± 0.18** | **-2.17 ± 0.08** |
| Mm.23596 | Cenpm | Centromere protein M | **-2.25 ± 0.25** | **-2.38 ± 0.44** |
| Mm.281805 | Mbl1 | Mannose-binding lectin (protein A) 1 | **-2.26 ± 0.31** | **-1.81 ± 0.20** |
| Mm.337820 | Tns3 | Tensin 3 | **-2.30 ± 0.45** | **-2.09 ± 0.06** |
| Mm.43358 | Pbx1 | Pre B-cell leukemia transcription factor 1 | **-2.32 ± 0.22** | **-3.11 ± 0.24** |
| Mm.103439 | Gprc5b | G protein-coupled receptor, family C, group 5, member B | **-2.34 ± 0.13** | **-2.31 ± 0.04** |
| Mm.35650 | Tspan31 | Tetraspanin 31 | **-2.37 ± 0.33** | **-3.11 ± 0.20** |
| Mm.18344 | Psmc3ip | Proteasome (prosome, macropain) 26S subunit, ATPase 3, interacting protein | **-2.37 ± 0.24** | **-2.94 ± 0.13** |
| Mm.41580 | Rab3b | RAB3B, member RAS oncogene family | **-2.40 ± 0.19** | **-2.16 ± 0.15** |
| Mm.347934 | 5430421N21Rik | RIKEN cDNA 5430421N21 gene | **-2.40 ± 0.38** | **-2.23 ± 0.06** |
| Mm.276696 | Armc3 | Armadillo repeat containing 3 | **-2.42 ± 0.36** | **-3.48 ± 0.14** |
| Mm.46480 | Tmem41a | Transmembrane protein 41a | **-2.43 ± 0.28** | **-1.66 ± 0.09** |
| Mm.207484 | Tmem91 | Transmembrane protein 91 | **-2.51 ± 0.27** | **-3.05 ± 0.06** |
| Mm.115970 | Adamts16 | A disintegrin-like and metallopeptidase (reprolysin type) with thrombospondin type 1 motif, 16 | **-2.53 ± 0.28** | **-2.69 ± 0.03** |
| Mm.194536 | Gmfg | Glia maturation factor, gamma | **-2.54 ± 0.26** | **-2.68 ± 0.27** |
| Mm.339812 | Fgf18 | Fibroblast growth factor 18 | **-2.54 ± 0.45** | **-2.66 ± 0.18** |
| Mm.443244 | Fam71b | Family with sequence similarity 71, member B | **-2.55 ± 0.26** | **-2.83 ± 0.09** |
| Mm.368330 | Il13ra2 | Interleukin 13 receptor, alpha 2 | **-2.56 ± 0.27** | **-1.81 ± 0.09** |
| Mm.246858 | Sprn | Shadow of prion protein | **-2.57 ± 0.33** | **-3.36 ± 0.20** |
| Mm.213406 | Uggt2 | UDP-glucose glycoprotein glucosyltransferase 2 | **-2.63 ± 0.17** | **-2.78 ± 0.10** |
| Mm.1685 | Plxna3 | Plexin A3 | **-2.63 ± 0.26** | **-2.13 ± 0.21** |
| Mm.440867 | Slc6a20a | Solute carrier family 6 (neurotransmitter transporter), member 20A | **-2.65 ± 0.47** | **-2.72 ± 0.39** |
| Mm.290729 | Slc43a3 | Solute carrier family 43, member 3 | **-2.73 ± 0.20** | **-2.72 ± 0.26** |
| Mm.23881 | 1600002H07Rik | RIKEN cDNA 1600002H07 gene | **-2.78 ± 0.16** | **-2.47 ± 0.20** |
| Mm.222636 | Vmn1r235 | Vomeronasal 1 receptor 235 | **-2.87 ± 0.45** | **-2.54 ± 0.13** |
| Mm.249065 | Tcp11l1 | T-complex 11 like 1 | **-2.89 ± 0.32** | **-2.58 ± 0.08** |
| Mm.483877 | Usp44 | Ubiquitin specific peptidase 44 | **-2.9 ± 0.16** | **-3.55 ± 0.21** |
| Mm.485832 | Nphp1 | Nephronophthisis 1 (juvenile) homolog | **-2.91 ± 0.17** | **-2.78 ± 0.18** |
| Mm.155877 | Ulk3 | Unc-51-like kinase 3 | **-2.95 ± 0.24** | **-4.02 ± 0.16** |
| Mm.18526 | Ehd3 | EH-domain containing 3 | **-2.99 ± 0.49** | **-3.32 ± 0.52** |
| Mm.34002 | 2410015M20Rik | RIKEN cDNA 2410015M20 gene | **-3.05 ± 0.18** | **-3.23 ± 0.20** |
| Mm.260103 | Cep164 | Centrosomal protein 164 | **-3.07 ± 0.13** | **-3.42 ± 0.63** |
| Mm.373672 | Ttn | Titin | **-3.09 ± 0.47** | **-3.29 ± 0.05** |
| Mm.265990 | D16H22S680E | DNA segment, Chr 16, human D22S680E, expressed | **-3.14 ± 0.44** | **-2.67 ± 0.41** |
| Mm.181836 | Mphosph6 | M phase phosphoprotein 6 | **-3.15 ± 0.33** | **-3.30 ± 0.38** |
| Mm.28890 | Naaa | N-acylethanolamine acid amidase | **-3.16 ± 0.44** | **-3.03 ± 0.26** |
| Mm.128627 | Dync1li1 | Dynein cytoplasmic 1 light intermediate chain 1 | **-3.21 ± 0.53** | **-2.80 ± 0.11** |
| Mm.485146 | Sipa1l3 | Signal-induced proliferation-associated 1 like 3 | **-3.22 ± 0.55** | **-4.08 ± 0.13** |
| Mm.377477 | Olfr1325 | Olfactory receptor 1325 | **-3.23 ± 0.10** | **-3.39 ± 0.05** |
| Mm.294826 | Hoxa7 | Homeobox A7 | **-3.24 ± 0.12** | **-2.75 ± 0.04** |
| Mm.334193 | Mier2 | Mesoderm induction early response 1, family member 2 | **-3.38 ± 0.37** | **-3.72 ± 0.33** |
| Mm.443529 | Arhgap20 | Rho GTPase activating protein 20 | **-3.58 ± 0.29** | **-3.58 ± 0.06** |
| Mm.440026 | Ilf3 | Interleukin enhancer binding factor 3 | **-3.65 ± 0.08** | **-3.67 ± 0.28** |
| Mm.273997 | Ppp2r2a | Protein phosphatase 2 (formerly 2A), regulatory subunit B (PR 52), alpha isoform | **-3.84 ± 0.08** | **-4.51 ± 0.15** |
| Mm.186936 | Rbfa | Ribosome binding factor A | **-4.28 ± 0.56** | **-5.78 ± 0.04** |
| Mm.131237 | Stap1 | Signal transducing adaptor family member 1 | **-4.35 ± 0.57** | **-4.78 ± 0.28** |
| Mm.89943 | Adam18 | A disintegrin and metallopeptidase domain 18 | **-4.88 ± 0.56** | **-5.71 ± 0.30** |
| Mm.377086 | Hoxa13 | Homeobox A13 | **-4.98 ± 0.44** | **-5.38 ± 0.09** |
| **Genes common at 72 h pi** | | | | |
| Mm.21567 | S100a8 | S100 calcium binding protein A8 (calgranulin A) | **3.97 ± 0.16** | **3.92 ± 0.84** |
| Mm.141021 | Ifitm3 | Interferon induced transmembrane protein 3 | **3.22 ± 0.24** | **3.91 ± 0.20** |
| Mm.39825 | Lmx1b | LIM homeobox transcription factor 1 beta | **2.79 ± 0.35** | **4.99 ± 0.11** |
| Mm.425949 | Ly6a | Lymphocyte antigen 6 complex, locus A | **2.70 ± 0.53** | **3.35 ± 0.20** |
| Mm.175661 | Ifitm1 | Interferon induced transmembrane protein 1 | **2.39 ± 0.14** | **1.67 ± 0.25** |
| Mm.439732 | C1qc | Complement component 1, q subcomponent, C chain | **2.32 ± 0.18** | **2.51 ± 0.31** |
| Mm.24769 | Ifi47 | Interferon gamma inducible protein 47 | **2.23 ± 0.16** | **2.80 ± 0.33** |
| Mm.271275 | Ifi27l2a | Interferon, alpha-inducible protein 27 like 2A | **2.20 ± 0.31** | **3.00 ± 0.18** |
| Mm.349432 | Gm5331 | PREDICTED: predicted gene 5331 | **2.12 ± 0.23** | **1.93 ± 0.27** |
| Mm.33443 | Zfp677 | Zinc finger protein 677 | **2.02 ± 0.23** | **3.50 ± 0.17** |
| Mm.379266 | Ifitm2 | Interferon induced transmembrane protein 2 | **1.99 ± 0.26** | **2.49 ± 0.27** |
| Mm.89991 | Psg18 | Pregnancy specific glycoprotein 18 | **1.95 ± 0.35** | **2.91 ± 0.15** |
| Mm.116687 | Zbp1 | Z-DNA binding protein 1 | **1.90 ± 0.29** | **2.56 ± 0.29** |
| Mm.41339 | Myo5c | Myosin VC | **1.83 ± 0.26** | **1.07 ± 0.16** |
| Mm.1114 | Gla | Galactosidase, alpha | **1.79 ± 0.24** | **2.22 ± 0.09** |
| Mm.238343 | Anxa2 | Annexin A2 | **1.77 ± 0.33** | **2.38 ± 0.20** |
| Mm.261270 | Ifi204 | Interferon activated gene 204 | **1.76 ± 0.17** | **1.96 ± 0.14** |
| Mm.358954 | Hist2h2ac | Histone cluster 2, h2ac | **1.75 ± 0.21** | **2.36 ± 0.21** |
| Mm.28212 | Sepx1 | Selenoprotein X 1 | **1.71 ± 0.25** | **2.53 ± 0.21** |
| Mm.269029 | Slc7a6os | Solute carrier family 7, member 6 opposite strand | **1.71 ± 0.25** | **2.97 ± 0.29** |
| Mm.9901 | Nucb2 | Nucleobindin 2 | **1.58 ± 0.15** | **1.86 ± 0.06** |
| Mm.26135 | Cab39 | Calcium binding protein 39 | **1.53 ± 0.19** | **1.22 ± 0.07** |
| Mm.76649 | Vcam1 | Vascular cell adhesion molecule 1 | **1.45 ± 0.07** | **1.96 ± 0.30** |
| Mm.276325 | Sod1 | Superoxide dismutase 1, soluble | **1.40 ± 0.24** | **1.58 ± 0.29** |
| Mm.299312 | Nhp2l1 | NHP2 non-histone chromosome protein 2-like 1 | **1.35 ± 0.12** | **1.73 ± 0.12** |
| Mm.426537 | LOC100041885 | PREDICTED: sp110 nuclear body protein-like | **1.23 ± 0.19** | **2.77 ± 0.11** |
| Mm.261973 | Hist1h2ac | Histone cluster 1, h2ac | **1.22 ± 0.17** | **1.79 ± 0.39** |
| Mm.1155 | Tuba4a | Tubulin, alpha 4A | **1.18 ± 0.06** | **1.96 ± 0.38** |
| Mm.297275 | Clec4a1 | C-type lectin domain family 4, member a1 | **1.15 ± 0.15** | **2.65 ± 0.44** |
| Mm.41864 | Ubap2 | Ubiquitin-associated protein 2 | **1.14 ± 0.11** | **2.55 ± 0.17** |
| Mm.231395 | Ctsd | Cathepsin D | **1.14 ± 0.18** | **1.53 ± 0.29** |
| Mm.339760 | Smcr7 | Smith-Magenis syndrome chromosome region, candidate 7 homolog | **1.13 ± 0.25** | **1.82 ± 0.22** |
| Mm.371544 | Slc25a5 | Solute carrier family 25 (mitochondrial carrier, adenine nucleotide translocator), member 5 | **1.12 ± 0.22** | **1.15 ± 0.23** |
| Mm.91920 | Socs6 | Suppressor of cytokine signaling 6 | **1.11 ± 0.14** | **1.03 ± 0.22** |
| Mm.29586 | Basp1 | Brain abundant, membrane attached signal protein 1 | **-1.11 ± 0.17** | **-1.51 ± 0.22** |
| Mm.386829 | Tsga10ip | PREDICTED: testis specific 10 interacting protein | **-1.33 ± 0.14** | **-1.69 ± 0.17** |
| Mm.3951 | Thy1 | Thymus cell antigen 1, theta | **-1.35 ± 0.14** | **-1.13 ± 0.14** |
| Mm.193212 | Hyi | Hydroxypyruvate isomerase homolog | **-1.47 ± 0.32** | **-1.40 ± 0.23** |
| Mm.359982 | 2310014H01Rik | RIKEN cDNA 2310014H01 gene | **-1.52 ± 0.31** | **-1.02 ± 0.11** |
| Mm.57734 | Lims1 | LIM and senescent cell antigen-like domains 1 | **-1.59 ± 0.29** | **-2.32 ± 0.32** |
| Mm.119274 | Prrxl1 | Paired related homeobox protein-like 1 | **-1.65 ± 0.21** | **-2.42 ± 0.30** |
| Mm.102305 | Tnrc6a | Trinucleotide repeat containing 6a | **-2.03 ± 0.32** | **-1.03 ± 0.23** |
| Mm.288567 | Beta-s | Hemoglobin subunit beta-1-like | **-2.22 ± 0.37** | **1.94 ± 0.14** |
| Mm.483321 | Bmp10 | Bone morphogenetic protein 10 | **-2.53 ± 0.52** | **-2.28 ± 0.51** |
| Mm.170905 | Fyb | FYN binding protein | **-2.69 ± 0.30** | **-2.00 ± 0.31** |
| Mm.443244 | Fam71b | Family with sequence similarity 71, member B | **-4.23 ± 0.20** | **-3.85 ± 0.52** |
| Mm.458815 | Ccl21a | Chemokine (C-C motif) ligand 21A (serine) | **-4.73 ± 0.48** | **-1.59 ± 0.21** |
| Mm.276696 | Armc3 | Armadillo repeat containing 3 | **-6.25 ± 0.37** | **-5.65 ± 0.58** |
