# Supplementary material for: Differential host gene responses from infection with neurovirulent and partially-neurovirulent strains of Venezuelan equine encephalitis virus
Source: BMC Infect Dis. 2017 Apr 26;17:309. doi: 10.1186/s12879-017-2355-3 (PMC5405508; doi:10.1186/s12879-017-2355-3)
Supplement: Supplementary file 3 — Significantly modulated genes common against V3000 and V3034 strains of VEEV in brain. Genes that were modulated with both V3000 and V3034 infection in the brain were identified. The list summarizes the commonly modulated genes for each time point studied. Values are expressed as average values of (log2) fold expression for each gene over uninfected controls ± standard error mean (SEM). * P ≤ 0.05. (DOCX 43 kb) [file 12879_2017_2355_MOESM3_ESM.docx]

**Additional file 3: Table S2: Significantly modulated genes common against V3000 and V3034 strains of VEEV in brain**

| **UniGene** | **Gene** | **Description** | **Log_2_ Exp ± SEM** | |
| --- | --- | --- | --- | --- |
|  |  |  | **V3000** | **V3034** |
| **Genes common at 48 h pi** | | | | |
| Mm.260325 | Bst2 | Bone marrow stromal cell antigen 2 | **2.63 ± 0.47** | **2.31 ± 0.41** |
| Mm.376121 | Olfr1152 | Olfactory receptor 1152 | **2.61 ± 0.44** | **1.68 ± 0.16** |
| Mm.196581 | Mapk1 | Mitogen-activated protein kinase 1 | **2.59 ± 0.39** | **2.18 ± 0.42** |
| Mm.163 | B2m | Beta-2 microglobulin | **2.26 ± 0.08** | **2.14 ± 0.15** |
| Mm.276739 | Sox10 | SRY-box containing gene 10 | **2.09 ± 0.15** | **2.04 ± 0.18** |
| Mm.439743 | H2-Q7 | Histocompatibility 2, Q region locus 7 | **2.03 ± 0.11** | **1.27 ± 0.28** |
| Mm.196013 | Samd9l | Sterile alpha motif domain containing 9-like | **2.00 ± 0.27** | **1.45 ± 0.13** |
| Mm.8369 | Mst1 | Macrophage stimulating 1 | **1.99 ± 0.02** | **2.15 ± 0.36** |
| Mm.110505 | Igsf1 | Immunoglobulin superfamily, member 1 | **1.96 ± 0.37** | **1.49 ± 0.23** |
| Mm.32881 | Spnb1 | Spectrin beta 1 | **1.94 ± 0.13** | **1.57 ± 0.16** |
| Mm.22948 | Ubtd1 | Ubiquitin domain containing 1 | **1.93 ± 0.11** | **1.37 ± 0.08** |
| Mm.269029 | Slc7a6os | Solute carrier family 7, member 6 opposite strand | **1.88 ± 0.12** | **1.65 ± 0.20** |
| Mm.13944 | Rps9 | Ribosomal protein S9 | **1.82 ± 0.10** | **1.61 ± 0.14** |
| Mm.132226 | Ehd4 | EH-domain containing 4 | **1.81 ± 0.21** | **1.55 ± 0.20** |
| Mm.272115 | Myom2 | Myomesin 2 | **1.80 ± 0.17** | **1.61 ± 0.29** |
| Mm.347647 | A530023O14Rik | RIKEN cDNA A530023O14 gene | **1.73 ± 0.06** | **1.46 ± 0.21** |
| Mm.39825 | Lmx1b | LIM homeobox transcription factor 1 beta | **1.66 ± 0.27** | **1.81 ± 0.29** |
| Mm.256414 | Slc9a2 | Solute carrier family 9 (sodium/hydrogen exchanger), member 2 | **1.64 ± 0.15** | **1.33 ± 0.18** |
| Mm.20079 | Calml3 | Calmodulin-like 3 | **1.58 ± 0.18** | **1.66 ± 0.26** |
| Mm.323595 | Tob2 | Transducer of ERBB2, 2 | **1.50 ± 0.25** | **1.81 ± 0.36** |
| Mm.4298 | En2 | Engrailed 2 | **1.40 ± 0.19** | **1.01 ± 0.11** |
| Mm.241682 | Lrrc4c | Leucine rich repeat containing 4C | **1.35 ± 0.16** | **1.23 ± 0.24** |
| Mm.188108 | Gm1574 | Predicted gene 1574 | **1.31 ± 0.15** | **1.09 ± 0.05** |
| Mm.33443 | Zfp677 | Zinc finger protein 677 | **1.31 ± 0.10** | **1.20 ± 0.02** |
| Mm.89991 | Psg18 | Pregnancy specific glycoprotein 18 | **1.27 ± 0.22** | **1.23 ± 0.23** |
| Mm.265929 | Vti1b | Vesicle transport through interaction with t-snares 1B homolog | **1.14 ± 0.12** | **1.06 ± 0.23** |
| Mm.275800 | Slc12a7 | Solute carrier family 12, member 7 | **1.10 ± 0.14** | **1.13 ± 0.14** |
| Mm.29586 | Basp1 | Brain abundant, membrane attached signal protein 1 | **-1.04 ± 0.21** | **-1.13 ± 0.18** |
| Mm.213651 | Gk5 | Glycerol kinase 5 (putative) | **-1.10 ± 0.04** | **-1.36 ± 0.17** |
| Mm.281452 | P2ry1 | Purinergic receptor P2Y, G-protein coupled 1 | **-1.15 ± 0.24** | **-1.45 ± 0.10** |
| Mm.336054 | Gm595 | Predicted gene 595 | **-1.29 ± 0.21** | **-1.16 ± 0.24** |
| Mm.227117 | Slc30a10 | Solute carrier family 30, member 10 | **-1.31 ± 0.08** | **-1.00 ± 0.17** |
| Mm.331893 | Hmgb4 | High-mobility group box 4 | **-1.34 ± 0.29** | **-1.35 ± 0.27** |
| Mm.247775 | Hook2 | Hook homolog 2 | **-1.36 ± 0.10** | **-1.45 ± 0.03** |
| Mm.43358 | Pbx1 | Pre B-cell leukemia transcription factor 1 | **-1.38 ± 0.19** | **-1.14 ± 0.17** |
| Mm.219475 | Wdr20b | WD repeat domain 20b | **-1.40 ± 0.19** | **-1.34 ± 0.06** |
| Mm.170905 | Fyb | FYN binding protein | **-1.41 ± 0.16** | **-1.14 ± 0.04** |
| Mm.30204 | Qdpr | Quinoid dihydropteridine reductase | **-1.43 ± 0.06** | **-1.51 ± 0.10** |
| Mm.181836 | Mphosph6 | M phase phosphoprotein 6 | **-1.50 ± 0.34** | **-1.78 ± 0.23** |
| Mm.139815 | Tcf7l2 | Transcription factor 7-like 2, T-cell specific, HMG-box | **-1.52 ± 0.26** | **-1.56 ± 0.29** |
| Mm.34002 | 2410015M20Rik | RIKEN cDNA 2410015M20 gene | **-1.52 ± 0.04** | **-1.56 ± 0.17** |
| Mm.271711 | Tagln2 | Transgelin 2 | **-1.52 ± 0.17** | **-1.83 ± 0.18** |
| Mm.449996 | 4921506M07Rik | RIKEN cDNA 4921506M07 gene | **-1.59 ± 0.12** | **-1.35 ± 0.22** |
| Mm.390986 | Dhx16 | DEAH (Asp-Glu-Ala-His) box polypeptide 16 | **-1.68 ± 0.08** | **-1.65 ± 0.21** |
| Mm.443244 | Fam71b | Family with sequence similarity 71, member B | **-1.72 ± 0.04** | **-1.76 ± 0.31** |
| Mm.440026 | Ilf3 | Interleukin enhancer binding factor 3 | **-1.75 ± 0.11** | **-1.54 ± 0.07** |
| Mm.85280 | Jakmip1 | Janus kinase and microtubule interacting protein 1 | **-1.78 ± 0.25** | **-2.08 ± 0.33** |
| Mm.55952 | Adam26b | A disintegrin and metallopeptidase domain 26B | **-1.80 ± 0.17** | **-1.37 ± 0.28** |
| Mm.458006 | Id4 | Inhibitor of DNA binding 4 | **-1.86 ± 0.13** | **-1.35 ± 0.14** |
| Mm.118004 | Vangl1 | Vang-like 1 | **-1.96 ± 0.17** | **-2.03 ± 0.32** |
| Mm.297199 | Samd14 | Sterile alpha motif domain containing 14 | **-1.96 ± 0.16** | **-1.66 ± 0.24** |
| Mm.18344 | Psmc3ip | Proteasome (prosome, macropain) 26S subunit, ATPase 3, interacting protein | **-2.03 ± 0.32** | **-1.69 ± 0.22** |
| Mm.296181 | Hspa2 | Heat shock protein 2 | **-2.05 ± 0.29** | **-2.05 ± 0.27** |
| Mm.252987 | Slc12a5 | Solute carrier family 12, member 5 | **-2.05 ± 0.30** | **-2.12 ± 0.41** |
| Mm.3468 | Socs3 | Suppressor of cytokine signaling 3 | **-2.11 ± 0.13** | **-1.37 ± 0.27** |
| Mm.249775 | Ccdc79 | Coiled-coil domain containing 79 | **-2.13 ± 0.18** | **-1.55 ± 0.20** |
| Mm.260103 | Cep164 | Centrosomal protein 164 | **-2.14 ± 0.22** | **-1.84 ± 0.32** |
| Mm.250732 | Lhx9 | LIM homeobox protein 9 | **-2.25 ± 0.35** | **-1.67 ± 0.12** |
| Mm.131237 | Stap1 | Signal transducing adaptor family member 1 | **-2.27 ± 0.34** | **-2.22 ± 0.29** |
| Mm.194536 | Gmfg | Glia maturation factor, gamma | **-2.29 ± 0.23** | **-2.34 ± 0.36** |
| Mm.358731 | Cnot3 | CCR4-NOT transcription complex, subunit 3 | **-2.34 ± 0.30** | **-2.38 ± 0.31** |
| Mm.186936 | 1110032A13Rik | RIKEN cDNA 1110032A13 gene | **-2.51 ± 0.53** | **-2.26 ± 0.49** |
| Mm.23049 | Filip1 | Filamin A interacting protein 1 | **-2.56 ± 0.47** | **-2.32 ± 0.43** |
| Mm.478913 | Sipa1l3 | Signal-induced proliferation-associated 1 like 3 | **-2.60 ± 0.22** | **-1.76 ± 0.38** |
| Mm.11350 | Zbtb42 | Zinc finger and BTB domain containing 42 | **-2.61 ± 0.54** | **-2.20 ± 0.38** |
| Mm.441431 | Syn2 | Synapsin II | **-2.74 ± 0.34** | **-2.31 ± 0.46** |
| Mm.2581 | Epha2 | Eph receptor A2 | **-3.37 ± 0.43** | **-2.71 ± 0.61** |
| Mm.27949 | Sh3rf1 | SH3 domain containing ring finger 1 | **-3.38 ± 0.32** | **-2.41 ± 0.55** |
| Mm.20355 | Wnt4 | Wingless-related MMTV integration site 4 | **-3.47 ± 0.73** | **-3.19 ± 0.65** |
| Mm.251227 | Klk7 | Kallikrein related-peptidase 7 (chymotryptic, stratum corneum) | **-3.70 ± 0.49** | **-2.79 ± 0.60** |
| **Genes common at 72 h pi** | | | | |
| Mm.9714 | Gdf9 | Growth differentiation factor 9 | **3.66 ± 0.61** | **3.40 ± 0.69** |
| Mm.376121 | Olfr1152 | Olfactory receptor 1152 | **2.97 ± 0.51** | **2.38 ± 0.23** |
| Mm.163 | B2m | Beta-2 microglobulin | **2.90 ± 0.26** | **2.49 ± 0.34** |
| Mm.24125 | Col4a3bp | Collagen, type IV, alpha 3 (Goodpasture antigen) binding protein | **2.65 ± 0.34** | **2.84 ± 0.49** |
| Mm.8369 | Mst1 | Macrophage stimulating 1 | **2.59 ± 0.28** | **2.41 ± 0.53** |
| Mm.341423 | Dock4 | Dedicator of cytokinesis 4 | **2.47 ± 0.14** | **2.13 ± 0.29** |
| Mm.377828 | Olfr547 | Olfactory receptor 547 | **2.47 ± 0.41** | **1.39 ± 0.10** |
| Mm.272115 | Myom2 | Myomesin 2 | **2.36 ± 0.18** | **2.27 ± 0.26** |
| Mm.110505 | Igsf1 | Immunoglobulin superfamily, member 1 | **2.27 ± 0.32** | **1.75 ± 0.09** |
| Mm.20079 | Calml3 | Calmodulin-like 3 | **2.05 ± 0.23** | **1.92 ± 0.36** |
| Mm.20440 | Htr4 | 5 hydroxytryptamine (serotonin) receptor 4 | **1.94 ± 0.19** | **1.64 ± 0.09** |
| Mm.347647 | A530023O14Rik | RIKEN cDNA A530023O14 gene | **1.93 ± 0.13** | **1.60 ± 0.12** |
| Mm.32881 | Spnb1 | Spectrin beta 1 | **1.87 ± 0.28** | **1.57 ± 0.09** |
| Mm.259105 | Odf2l | Outer dense fiber of sperm tails 2-like | **1.72 ± 0.39** | **1.56 ± 0.20** |
| Mm.290764 | Lrrc61 | Leucine rich repeat containing 61 | **1.66 ± 0.09** | **1.46 ± 0.20** |
| Mm.13944 | Rps9 | Ribosomal protein S9 | **1.64 ± 0.02** | **1.57 ± 0.25** |
| Mm.431282 | H2-Q6 | Histocompatibility 2, Q region locus 6 | **1.59 ± 0.14** | **1.15 ± 0.18** |
| Mm.7454 | Igbp1 | Immunoglobulin (CD79A) binding protein 1 | **1.50 ± 0.09** | **1.28 ± 0.02** |
| Mm.188108 | Gm1574 | Predicted gene 1574 | **1.45 ± 0.08** | **1.11 ± 0.04** |
| Mm.256414 | Slc9a2 | Solute carrier family 9 (sodium/hydrogen exchanger), member 2 | **1.43 ± 0.13** | **1.19 ± 0.04** |
| Mm.34428 | Pias4 | Protein inhibitor of activated STAT 4 | **1.40 ± 0.19** | **1.10 ± 0.10** |
| Mm.33443 | Zfp677 | Zinc finger protein 677 | **1.38 ± 0.12** | **1.32 ± 0.14** |
| Mm.241682 | Lrrc4c | Leucine rich repeat containing 4C | **1.34 ± 0.06** | **1.47 ± 0.08** |
| Mm.195803 | Nnt | Nicotinamide nucleotide transhydrogenase | **1.34 ± 0.10** | **1.01 ± 0.19** |
| Mm.323595 | Tob2 | Transducer of ERBB2, 2 | **1.26 ± 0.18** | **1.81 ± 0.27** |
| Mm.259916 | Chdh | Choline dehydrogenase | **1.22 ± 0.08** | **1.29 ± 0.17** |
| Mm.386931 | Ecm2 | Extracellular matrix protein 2, female organ and adipocyte specific | **1.13 ± 0.08** | **1.04 ± 0.15** |
| Mm.40655 | Fam185a | Family with sequence similarity 185, member A | **1.11 ± 0.13** | **3.09 ± 0.29** |
| Mm.89991 | Psg18 | Pregnancy specific glycoprotein 18 | **1.07 ± 0.14** | **1.48 ± 0.32** |
| Mm.1114 | Gla | Galactosidase, alpha | **1.04 ± 0.15** | **1.09 ± 0.19** |
| Mm.54120 | Samd4b | Sterile alpha motif domain containing 4B | **-1.02 ± 0.11** | **-1.30 ± 0.11** |
| Mm.386757 | Clcn1 | Chloride channel 1 | **-1.04 ± 0.20** | **-1.58 ± 0.20** |
| Mm.443529 | Arhgap20 | Rho GTPase activating protein 20 | **-1.08 ± 0.11** | **-1.35 ± 0.14** |
| Mm.358682 | BC051019 | CDNA sequence BC051019 | **-1.09 ± 0.12** | **-1.27 ± 0.20** |
| Mm.327439 | AA792892 | Expressed sequence AA792892 | **-1.10 ± 0.03** | **-1.32 ± 0.06** |
| Mm.130824 | Npsr1 | Neuropeptide S receptor 1 | **-1.10 ± 0.20** | **-1.28 ± 0.06** |
| Mm.244068 | Arhgef7 | Rho guanine nucleotide exchange factor (GEF7) | **-1.11 ± 0.11** | **-1.46 ± 0.23** |
| Mm.103413 | Mul1 | Mitochondrial ubiquitin ligase activator of NFKB 1 | **-1.12 ± 0.13** | **-1.51 ± 0.19** |
| Mm.217354 | Mrps6 | Mitochondrial ribosomal protein S6 | **-1.13 ± 0.23** | **-1.25 ± 0.06** |
| Mm.390829 | Mtbp | Mdm2, transformed 3T3 cell double minute p53 binding protein | **-1.20 ± 0.13** | **-1.16 ± 0.25** |
| Mm.269088 | Anp32a | Acidic (leucine-rich) nuclear phosphoprotein 32 family, member A | **-1.21 ± 0.21** | **-1.06 ± 0.15** |
| Mm.26908 | Csnk1a1 | Casein kinase 1, alpha 1 | **-1.22 ± 0.19** | **-1.21 ± 0.12** |
| Mm.52711 | 0610030E20Rik | RIKEN cDNA 0610030E20 gene | **-1.29 ± 0.12** | **-1.61 ± 0.13** |
| Mm.390986 | Dhx16 | DEAH (Asp-Glu-Ala-His) box polypeptide 16 | **-1.29 ± 0.20** | **-1.61 ± 0.18** |
| Mm.297199 | Samd14 | Sterile alpha motif domain containing 14 | **-1.32 ± 0.10** | **-1.68 ± 0.21** |
| Mm.170905 | Fyb | FYN binding protein | **-1.33 ± 0.17** | **-1.32 ± 0.09** |
| Mm.219475 | Wdr20b | WD repeat domain 20b | **-1.36 ± 0.29** | **-1.20 ± 0.25** |
| Mm.296181 | Hspa2 | Heat shock protein 2 | **-1.44 ± 0.11** | **-2.08 ± 0.40** |
| Mm.73234 | Cep72 | Centrosomal protein 72 | **-1.45 ± 0.17** | **-1.07 ± 0.07** |
| Mm.138792 | Chd7 | Chromodomain helicase DNA binding protein 7 | **-1.46 ± 0.09** | **-1.60 ± 0.33** |
| Mm.13787 | Cp | Ceruloplasmin | **-1.50 ± 0.22** | **-1.47 ± 0.25** |
| Mm.227117 | Slc30a10 | Solute carrier family 30, member 10 | **-1.55 ± 0.17** | **-1.37 ± 0.12** |
| Mm.43358 | Pbx1 | Pre B-cell leukemia transcription factor 1 | **-1.60 ± 0.22** | **-1.51 ± 0.18** |
| Mm.281452 | P2ry1 | Purinergic receptor P2Y, G-protein coupled 1 | **-1.61 ± 0.20** | **-1.64 ± 0.10** |
| Mm.249775 | Ccdc79 | Coiled-coil domain containing 79 | **-1.65 ± 0.15** | **-1.92 ± 0.23** |
| Mm.260103 | Cep164 | Centrosomal protein 164 | **-1.70 ± 0.27** | **-2.04 ± 0.32** |
| Mm.247775 | Hook2 | Hook homolog 2 | **-1.72 ± 0.23** | **-1.61 ± 0.08** |
| Mm.118004 | Vangl1 | Vang-like 1 | **-1.74 ± 0.16** | **-2.29 ± 0.45** |
| Mm.443244 | Fam71b | Family with sequence similarity 71, member B | **-1.77 ± 0.22** | **-1.85 ± 0.27** |
| Mm.3468 | Socs3 | Suppressor of cytokine signaling 3 | **-1.77 ± 0.31** | **-1.72 ± 0.29** |
| Mm.34002 | 2410015M20Rik | RIKEN cDNA 2410015M20 gene | **-1.80 ± 0.27** | **-2.07 ± 0.29** |
| Mm.358731 | Cnot3 | CCR4-NOT transcription complex, subunit 3 | **-1.91 ± 0.16** | **-2.53 ± 0.54** |
| Mm.440026 | Ilf3 | Interleukin enhancer binding factor 3 | **-1.95 ± 0.25** | **-2.31 ± 0.34** |
| Mm.147213 | B230218P12Rik | PREDICTED: RIKEN cDNA B230218P12 gene, transcript variant 2 | **-2.01 ± 0.21** | **-4.21 ± 0.07** |
| Mm.458006 | Id4 | Inhibitor of DNA binding 4 | **-2.06 ± 0.31** | **-2.05 ± 0.16** |
| Mm.373672 | Ttn | Titin | **-2.08 ± 0.26** | **-1.70 ± 0.25** |
| Mm.246858 | Sprn | Shadow of prion protein | **-2.17 ± 0.39** | **-2.30 ± 0.25** |
| Mm.276331 | Usp44 | Ubiquitin specific peptidase 44 | **-2.19 ± 0.36** | **-1.81 ± 0.31** |
| Mm.11350 | Zbtb42 | Zinc finger and BTB domain containing 42 | **-2.20 ± 0.34** | **-2.68 ± 0.59** |
| Mm.250732 | Lhx9 | LIM homeobox protein 9 | **-2.22 ± 0.28** | **-2.48 ± 0.36** |
| Mm.18344 | Psmc3ip | Proteasome (prosome, macropain) 26S subunit, ATPase 3, interacting protein | **-2.25 ± 0.38** | **-2.14 ± 0.22** |
| Mm.447819 | Klk1b1 | Kallikrein 1-related peptidase b1 | **-2.26 ± 0.36** | **-2.07 ± 0.14** |
| Mm.276696 | Armc3 | Armadillo repeat containing 3 | **-2.32 ± 0.39** | **-2.10 ± 0.30** |
| Mm.131237 | Stap1 | Signal transducing adaptor family member 1 | **-2.36 ± 0.31** | **-2.79 ± 0.60** |
| Mm.273997 | Ppp2r2a | Protein phosphatase 2 (formerly 2A), regulatory subunit B (PR 52), alpha isoform | **-2.45 ± 0.29** | **-2.58 ± 0.34** |
| Mm.258300 | Agpat4 | 1-acylglycerol-3-phosphate O-acyltransferase 4 | **-2.53 ± 0.45** | **-2.15 ± 0.28** |
| Mm.233914 | Ccdc64 | Coiled-coil domain containing 64 | **-2.71 ± 0.41** | **-2.99 ± 0.48** |
| Mm.478913 | Sipa1l3 | Signal-induced proliferation-associated 1 like 3 | **-2.92 ± 0.54** | **-2.87 ± 0.44** |
| Mm.377086 | Hoxa13 | Homeobox A13 | **-3.05 ± 0.60** | **-3.04 ± 0.49** |
| Mm.2581 | Epha2 | Eph receptor A2 | **-3.42 ± 0.71** | **-3.77 ± 0.86** |
| Mm.251227 | Klk7 | Kallikrein related-peptidase 7 (chymotryptic, stratum corneum) | **-3.47 ± 0.72** | **-3.78 ± 0.77** |
| Mm.22842 | Cd2 | CD2 antigen | **-3.91 ± 0.75** | **-3.73 ± 0.67** |
| Mm.255063 | Wrb | Tryptophan rich basic protein | **-4.39 ± 0.68** | **-4.33 ± 0.86** |
| Mm.250732 | Lhx9 | LIM homeobox protein 9 | **-2.22 ± 0.28** | **-2.48 ± 0.36** |
| **Genes common at 96 h pi** | | | | |
| Mm.163 | B2m | Beta-2 microglobulin | **3.86 ± 0.17** | **2.67 ± 0.22** |
| Mm.141021 | Ifitm3 | Interferon induced transmembrane protein 3 | **3.83 ± 0.11** | **2.08 ± 0.24** |
| Mm.40965 | Nt5c2 | 5'-nucleotidase, cytosolic II | **3.44 ± 0.67** | **4.30 ± 0.25** |
| Mm.439743 | H2-Q7 | Histocompatibility 2, Q region locus 7 | **3.30 ± 0.44** | **1.79 ± 0.23** |
| Mm.9714 | Gdf9 | Growth differentiation factor 9 | **3.04 ± 0.63** | **3.49 ± 0.09** |
| Mm.461583 | Zfp456 | Zinc finger protein 456 | **2.33 ± 0.27** | **3.47 ± 0.23** |
| Mm.8369 | Mst1 | Macrophage stimulating 1 | **2.29 ± 0.31** | **2.69 ± 0.21** |
| Mm.272115 | Myom2 | Myomesin 2 | **2.26 ± 0.17** | **2.28 ± 0.14** |
| Mm.341423 | Dock4 | Dedicator of cytokinesis 4 | **2.19 ± 0.09** | **2.16 ± 0.31** |
| Mm.44176 | Efemp1 | Epidermal growth factor-containing fibulin-like extracellular matrix protein 1 | **2.17 ± 0.16** | **2.25 ± 0.35** |
| Mm.338001 | Pld5 | Phospholipase D family, member 5 | **1.91 ± 0.06** | **1.80 ± 0.25** |
| Mm.20079 | Calml3 | Calmodulin-like 3 | **1.89 ± 0.09** | **1.87 ± 0.18** |
| Mm.39825 | Lmx1b | LIM homeobox transcription factor 1 beta | **1.82 ± 0.14** | **2.28 ± 0.22** |
| Mm.28162 | Nup210 | Nucleoporin 210 | **1.76 ± 0.20** | **1.70 ± 0.17** |
| Mm.20440 | Htr4 | 5 hydroxytryptamine (serotonin) receptor 4 | **1.66 ± 0.12** | **2.12 ± 0.18** |
| Mm.110505 | Igsf1 | Immunoglobulin superfamily, member 1 | **1.65 ± 0.14** | **2.20 ± 0.01** |
| Mm.347647 | A530023O14Rik | RIKEN cDNA A530023O14 gene | **1.62 ± 0.02** | **1.89 ± 0.16** |
| Mm.13944 | Rps9 | Ribosomal protein S9 | **1.58 ± 0.09** | **1.64 ± 0.14** |
| Mm.195803 | Nnt | Nicotinamide nucleotide transhydrogenase | **1.45 ± 0.20** | **1.38 ± 0.16** |
| Mm.259105 | Odf2l | Outer dense fiber of sperm tails 2-like | **1.42 ± 0.10** | **1.63 ± 0.21** |
| Mm.7454 | Igbp1 | Immunoglobulin (CD79A) binding protein 1 | **1.28 ± 0.12** | **1.32 ± 0.08** |
| Mm.33443 | Zfp677 | Zinc finger protein 677 | **1.25 ± 0.03** | **1.51 ± 0.05** |
| Mm.22948 | Ubtd1 | Ubiquitin domain containing 1 | **1.24 ± 0.24** | **1.69 ± 0.15** |
| Mm.377828 | Olfr547 | Olfactory receptor 547 | **1.23 ± 0.13** | **1.78 ± 0.17** |
| Mm.30466 | Trps1 | Trichorhinophalangeal syndrome I | **1.17 ± 0.06** | **1.17 ± 0.10** |
| Mm.259916 | Chdh | Choline dehydrogenase | **1.09 ± 0.05** | **1.30 ± 0.11** |
| Mm.377402 | Olfr1511 | Olfactory receptor 1511 | **1.09 ± 0.08** | **1.57 ± 0.09** |
| Mm.89991 | Psg18 | Pregnancy specific glycoprotein 18 | **1.06 ± 0.15** | **1.22 ± 0.17** |
| Mm.332844 | Cyp3a11 | Cytochrome P450, family 3, subfamily a, polypeptide 11 | **-1.02 ± 0.11** | **-1.34 ± 0.20** |
| Mm.217354 | Mrps6 | Mitochondrial ribosomal protein S6 | **-1.04 ± 0.22** | **-1.40 ± 0.23** |
| Mm.291214 | Meaf6 | MYST/Esa1-associated factor 6 | **-1.05 ± 0.08** | **-1.23 ± 0.12** |
| Mm.296610 | 2210016F16Rik | RIKEN cDNA 2210016F16 gene | **-1.06 ± 0.13** | **-1.20 ± 0.25** |
| Mm.327439 | AA792892 | Expressed sequence AA792892 | **-1.10 ± 0.12** | **-1.30 ± 0.05** |
| Mm.443529 | Arhgap20 | Rho GTPase activating protein 20 | **-1.11 ± 0.19** | **-1.36 ± 0.09** |
| Mm.52711 | 0610030E20Rik | RIKEN cDNA 0610030E20 gene | **-1.13 ± 0.07** | **-1.27 ± 0.25** |
| Mm.215641 | Nagpa | N-acetylglucosamine-1-phosphodiester alpha-N-acetylglucosaminidase | **-1.13 ± 0.08** | **-1.10 ± 0.18** |
| Mm.332967 | Ube2h | Ubiquitin-conjugating enzyme E2H | **-1.14 ± 0.15** | **-1.57 ± 0.26** |
| Mm.170905 | Fyb | FYN binding protein | **-1.15 ± 0.18** | **-1.31 ± 0.10** |
| Mm.24214 | Foxk1 | Forkhead box K1 | **-1.15 ± 0.10** | **-1.39 ± 0.07** |
| Mm.479516 | Olfr819 | Olfactory receptor 819 | **-1.16 ± 0.19** | **-1.27 ± 0.12** |
| Mm.139815 | Tcf7l2 | Transcription factor 7-like 2, T-cell specific, HMG-box | **-1.16 ± 0.13** | **-1.01 ± 0.04** |
| Mm.103413 | Mul1 | Mitochondrial ubiquitin ligase activator of NFKB 1 | **-1.16 ± 0.02** | **-1.21 ± 0.10** |
| Mm.240252 | Trim14 | Tripartite motif-containing 14 | **-1.18 ± 0.04** | **-1.19 ± 0.08** |
| Mm.219475 | Wdr20b | WD repeat domain 20b | **-1.21 ± 0.22** | **-1.31 ± 0.22** |
| Mm.390986 | Dhx16 | DEAH (Asp-Glu-Ala-His) box polypeptide 16 | **-1.23 ± 0.17** | **-1.35 ± 0.05** |
| Mm.112977 | Eepd1 | Endonuclease/exonuclease/phosphatase family domain containing 1 | **-1.23 ± 0.22** | **-1.15 ± 0.21** |
| Mm.227117 | Slc30a10 | Solute carrier family 30, member 10 | **-1.24 ± 0.04** | **-1.50 ± 0.16** |
| Mm.297199 | Samd14 | Sterile alpha motif domain containing 14 | **-1.24 ± 0.18** | **-1.42 ± 0.12** |
| Mm.23782 | Glt25d2 | Glycosyltransferase 25 domain containing 2 | **-1.25 ± 0.14** | **-1.47 ± 0.11** |
| Mm.181836 | Mphosph6 | M phase phosphoprotein 6 | **-1.25 ± 0.02** | **-1.51 ± 0.20** |
| Mm.290868 | Tom1 | Target of myb1 homolog | **-1.32 ± 0.17** | **-1.44 ± 0.03** |
| Mm.103439 | Gprc5b | G protein-coupled receptor, family C, group 5, member B | **-1.35 ± 0.20** | **-1.74 ± 0.11** |
| Mm.333851 | Vpreb3 | Pre-B lymphocyte gene 3 | **-1.35 ± 0.09** | **-1.72 ± 0.15** |
| Mm.234667 | Il20ra | Interleukin 20 receptor, alpha | **-1.35 ± 0.22** | **-1.60 ± 0.12** |
| Mm.191949 | Camk1d | Calcium/calmodulin-dependent protein kinase ID | **-1.38 ± 0.26** | **-1.48 ± 0.06** |
| Mm.43358 | Pbx1 | Pre B-cell leukemia transcription factor 1 | **-1.38 ± 0.10** | **-1.49 ± 0.30** |
| Mm.246858 | Sprn | Shadow of prion protein | **-1.43 ± 0.20** | **-2.43 ± 0.26** |
| Mm.260103 | Cep164 | Centrosomal protein 164 | **-1.43 ± 0.28** | **-1.30 ± 0.19** |
| Mm.443244 | Fam71b | Family with sequence similarity 71, member B | **-1.43 ± 0.04** | **-1.90 ± 0.15** |
| Mm.296181 | Hspa2 | Heat shock protein 2 | **-1.44 ± 0.09** | **-1.60 ± 0.05** |
| Mm.138792 | Chd7 | Chromodomain helicase DNA binding protein 7 | **-1.44 ± 0.06** | **-1.95 ± 0.26** |
| Mm.213406 | Uggt2 | UDP-glucose glycoprotein glucosyltransferase 2 | **-1.51 ± 0.10** | **-1.95 ± 0.29** |
| Mm.252987 | Slc12a5 | Solute carrier family 12, member 5 | **-1.51 ± 0.34** | **-1.50 ± 0.15** |
| Mm.358731 | Cnot3 | CCR4-NOT transcription complex, subunit 3 | **-1.52 ± 0.08** | **-2.03 ± 0.05** |
| Mm.55952 | Adam26b | A disintegrin and metallopeptidase domain 26B | **-1.54 ± 0.11** | **-1.95 ± 0.24** |
| Mm.213651 | Gk5 | Glycerol kinase 5 | **-1.55 ± 0.14** | **-1.60 ± 0.20** |
| Mm.1351 | Hoxc4 | Homeobox C4 | **-1.57 ± 0.23** | **-1.66 ± 0.35** |
| Mm.3468 | Socs3 | Suppressor of cytokine signaling 3 | **-1.59 ± 0.16** | **-1.46 ± 0.13** |
| Mm.247775 | Hook2 | Hook homolog 2 | **-1.61 ± 0.14** | **-1.54 ± 0.30** |
| Mm.276331 | Usp44 | Ubiquitin specific peptidase 44 | **-1.62 ± 0.11** | **-1.72 ± 0.04** |
| Mm.34137 | 1700055N04Rik | PREDICTED: RIKEN cDNA 1700055N04 gene, transcript variant 1 | **-1.62 ± 0.17** | **-1.34 ± 0.16** |
| Mm.373672 | Ttn | Titin | **-1.64 ± 0.08** | **-1.67 ± 0.35** |
| Mm.115970 | Adamts16 | A disintegrin-like and metallopeptidase (reprolysin type) with thrombospondin type 1 motif, 16 | **-1.65 ± 0.12** | **-1.52 ± 0.21** |
| Mm.440026 | Ilf3 | Interleukin enhancer binding factor 3 | **-1.65 ± 0.14** | **-1.81 ± 0.16** |
| Mm.249775 | Ccdc79 | Coiled-coil domain containing 79 | **-1.69 ± 0.22** | **-1.37 ± 0.30** |
| Mm.153039 | Mpdz | Multiple PDZ domain protein | **-1.70 ± 0.09** | **-2.08 ± 0.10** |
| Mm.449996 | 4921506M07Rik | RIKEN cDNA 4921506M07 gene | **-1.71 ± 0.15** | **-1.78 ± 0.33** |
| Mm.118004 | Vangl1 | Vang-like 1 | **-1.72 ± 0.08** | **-1.91 ± 0.17** |
| Mm.23596 | Cenpm | Centromere protein M | **-1.72 ± 0.16** | **-1.95 ± 0.21** |
| Mm.18344 | Psmc3ip | Proteasome (prosome, macropain) 26S subunit, ATPase 3, interacting protein | **-1.75 ± 0.11** | **-2.35 ± 0.28** |
| Mm.334775 | Cenpt | Centromere protein T | **-1.77 ± 0.05** | **-1.77 ± 0.20** |
| Mm.155877 | Ulk3 | Unc-51-like kinase 3 | **-1.78 ± 0.09** | **-2.10 ± 0.45** |
| Mm.276696 | Armc3 | Armadillo repeat containing 3 | **-1.83 ± 0.14** | **-1.89 ± 0.19** |
| Mm.34002 | 2410015M20Rik | RIKEN cDNA 2410015M20 gene | **-1.87 ± 0.23** | **-1.55 ± 0.27** |
| Mm.131098 | Golga1 | Golgi autoantigen, golgin subfamily a, 1 | **-1.88 ± 0.23** | **-2.55 ± 0.20** |
| Mm.271724 | Dtx3 | Deltex 3 homolog | **-1.88 ± 0.19** | **-2.17 ± 0.11** |
| Mm.258300 | Agpat4 | 1-acylglycerol-3-phosphate O-acyltransferase 4 | **-1.93 ± 0.27** | **-1.59 ± 0.34** |
| Mm.11350 | Zbtb42 | Zinc finger and BTB domain containing 42 | **-1.96 ± 0.24** | **-1.99 ± 0.27** |
| Mm.30204 | Qdpr | Quinoid dihydropteridine reductase | **-1.97 ± 0.23** | **-1.49 ± 0.29** |
| Mm.458006 | Id4 | Inhibitor of DNA binding 4 | **-1.98 ± 0.07** | **-1.87 ± 0.34** |
| Mm.377086 | Hoxa13 | Homeobox A13 | **-1.99 ± 0.15** | **-2.32 ± 0.22** |
| Mm.27949 | Sh3rf1 | SH3 domain containing ring finger 1 | **-2.05 ± 0.06** | **-2.36 ± 0.25** |
| Mm.30533 | Gapt | Grb2-binding adaptor, transmembrane | **-2.09 ± 0.09** | **-2.46 ± 0.23** |
| Mm.60688 | Ccdc70 | Coiled-coil domain containing 70 | **-2.12 ± 0.14** | **-2.36 ± 0.14** |
| Mm.250732 | Lhx9 | LIM homeobox protein 9 | **-2.14 ± 0.17** | **-1.87 ± 0.31** |
| Mm.28839 | Eif2b1 | Eukaryotic translation initiation factor 2B, subunit 1 (alpha) | **-2.16 ± 0.33** | **-2.12 ± 0.23** |
| Mm.475174 | Far2 | Fatty acyl coa reductase 2 | **-2.19 ± 0.43** | **-2.65 ± 0.54** |
| Mm.23049 | Filip1 | Filamin A interacting protein 1 | **-2.19 ± 0.33** | **-2.21 ± 0.30** |
| Mm.441431 | Syn2 | Synapsin II | **-2.22 ± 0.23** | **-2.32 ± 0.06** |
| Mm.273997 | Ppp2r2a | Protein phosphatase 2 (formerly 2A), regulatory subunit B (PR 52), alpha isoform | **-2.23 ± 0.05** | **-2.66 ± 0.20** |
| Mm.19987 | Dct | Dopachrome tautomerase | **-2.25 ± 0.08** | **-2.95 ± 0.11** |
| Mm.478913 | Sipa1l3 | Signal-induced proliferation-associated 1 like 3 | **-2.25 ± 0.07** | **-2.28 ± 0.25** |
| Mm.314779 | LOC637277 | PREDICTED: similar to 145 kDa nucleolar protein | **-2.28 ± 0.14** | **-2.44 ± 0.50** |
| Mm.443660 | Lrrc4 | Leucine rich repeat containing 4 | **-2.29 ± 0.12** | **-2.32 ± 0.32** |
| Mm.203965 | Cand1 | Cullin associated and neddylation disassociated 1 | **-2.30 ± 0.10** | **-2.24 ± 0.40** |
| Mm.479524 | Nnat | Neuronatin | **-2.32 ± 0.28** | **-2.25 ± 0.45** |
| Mm.248081 | Zufsp | Zinc finger with UFM1-specific peptidase domain | **-2.36 ± 0.38** | **-2.38 ± 0.50** |
| Mm.186936 | 1110032A13Rik | RIKEN cDNA 1110032A13 gene | **-2.40 ± 0.06** | **-3.01 ± 0.33** |
| Mm.233914 | Ccdc64 | Coiled-coil domain containing 64 | **-2.48 ± 0.06** | **-3.11 ± 0.15** |
| Mm.194986 | Dgkg | Diacylglycerol kinase, gamma | **-2.58 ± 0.13** | **-3.63 ± 0.09** |
| Mm.20355 | Wnt4 | Wingless-related MMTV integration site 4 | **-2.62 ± 0.27** | **-3.06 ± 0.08** |
| Mm.22842 | Cd2 | CD2 antigen | **-2.65 ± 0.16** | **-3.28 ± 0.41** |
| Mm.2581 | Epha2 | Eph receptor A2 | **-2.67 ± 0.21** | **-3.01 ± 0.18** |
| Mm.89943 | Adam18 | A disintegrin and metallopeptidase domain 18 | **-2.78 ± 0.12** | **-2.92 ± 0.45** |
| Mm.251227 | Klk7 | Kallikrein related-peptidase 7 (chymotryptic, stratum corneum) | **-2.78 ± 0.11** | **-2.92 ± 0.48** |
| Mm.26768 | Spdef | SAM pointed domain containing ets transcription factor | **-2.85 ± 0.05** | **-3.49 ± 0.33** |
| Mm.173826 | Dym | Dymeclin | **-2.96 ± 0.17** | **-3.15 ± 0.37** |
| Mm.255063 | Wrb | Tryptophan rich basic protein | **-3.07 ± 0.27** | **-3.52 ± 0.31** |
| Mm.347407 | Cebpd | CCAAT/enhancer binding protein (C/EBP), delta | **-3.48 ± 0.36** | **-4.38 ± 0.13** |
| Mm.415 | Iapp | Islet amyloid polypeptide | **-3.59 ± 0.37** | **-4.22 ± 0.42** |
| Mm.196110 | Hba-a2 | Hemoglobin alpha, adult chain 2 | **-3.74 ± 0.24** | **-2.55 ± 0.50** |
