# Supplementary material for: Differential host gene responses from infection with neurovirulent and partially-neurovirulent strains of Venezuelan equine encephalitis virus
Source: BMC Infect Dis. 2017 Apr 26;17:309. doi: 10.1186/s12879-017-2355-3 (PMC5405508; doi:10.1186/s12879-017-2355-3)
Supplement: Supplementary file 4 — Significantly modulated genes in the spleen that were unique to V3000 infection. Genes that were modulated only with V3000 infection in the spleen were identified. The list summarizes the commonly modulated genes for each time point studied. Values are expressed as average values of (log2) fold expression for each gene over uninfected controls ± standard error mean (SEM). * P ≤ 0.05. (DOCX 82 kb) [file 12879_2017_2355_MOESM4_ESM.docx]

**Additional file 4: Table S3: Significantly modulated genes in spleen those were unique to spleen in V3000 infection**

| **UniGene** | **Gene** | **Description** | **Log_2_ Exp ± SEM** |
| --- | --- | --- | --- |
| **Genes uniquely modulated at 24 h pi** | | | |
| Mm.458519 | Gbp8 | Guanylate-binding protein 8 | **2.51 ± 0.20** |
| Mm.24045 | Rsad2 | Radical S-adenosyl methionine domain containing 2 | **2.47 ± 0.40** |
| Mm.33443 | Zfp677 | Zinc finger protein 677 | **2.42 ± 0.27** |
| Mm.447 | Pyhin1 | Pyrin and HIN domain family, member 1 | **2.35 ± 0.23** |
| Mm.211477 | Phldb2 | Pleckstrin homology-like domain, family B, member 2 | **2.33 ± 0.07** |
| Mm.7454 | Igbp1 | Immunoglobulin (CD79A) binding protein 1 | **2.33 ± 0.38** |
| Mm.485634 | Fpr2 | Formyl peptide receptor 2 | **2.30 ± 0.19** |
| Mm.479935 | Pydc4 | Pyrin domain containing 4 | **2.10 ± 0.24** |
| Mm.653 | Cfb | Complement factor B | **2.07 ± 0.31** |
| Mm.482320 | H2-Q8 | Histocompatibility 2, Q region locus 8 | **2.04 ± 0.40** |
| Mm.282556 | Npc2 | Niemann Pick type C2 | **1.97 ± 0.41** |
| Mm.317779 | LOC100042025 | PREDICTED: glyceraldehyde-3-phosphate dehydrogenase-like, transcript variant 2 | **1.96 ± 0.27** |
| Mm.22213 | Glipr2 | GLI pathogenesis-related 2 | **1.94 ± 0.28** |
| Mm.1114 | Gla | Galactosidase, alpha | **1.92 ± 0.20** |
| Mm.9537 | Lcn2 | Lipocalin 2 | **1.84 ± 0.41** |
| Mm.248615 | Lgals3 | Lectin, galactose binding, soluble 3 | **1.79 ± 0.15** |
| Mm.180191 | Psmb8 | Proteasome (prosome, macropain) subunit, beta type 8 | **1.78 ± 0.27** |
| Mm.390983 | Psmb9 | Proteasome (prosome, macropain) subunit, beta type 9 | **1.73 ± 0.22** |
| Mm.195061 | H2-T9 | Histocompatibility 2, T region locus 9 | **1.72 ± 0.27** |
| Mm.215667 | Hspe1 | Heat shock protein 1 (chaperonin 10) | **1.68 ± 0.10** |
| Mm.830 | Psme1 | Proteasome (prosome, macropain) 28 subunit, alpha | **1.67 ± 0.32** |
| Mm.276669 | Rab38 | RAB38, member of RAS oncogene family | **1.67 ± 0.35** |
| Mm.9901 | Nucb2 | Nucleobindin 2 | **1.62 ± 0.15** |
| Mm.28212 | Sepx1 | Selenoprotein X 1 | **1.61 ± 0.24** |
| Mm.431282 | H2-Q9 | Histocompatibility 2, Q region locus 9 | **1.53 ± 0.27** |
| Mm.32884 | Tuba8 | Tubulin, alpha 8 | **1.52 ± 0.11** |
| Mm.250418 | Ogfr | Opioid growth factor receptor | **1.52 ± 0.08** |
| Mm.208883 | Psma5 | Proteasome (prosome, macropain) subunit, alpha type 5 | **1.52 ± 0.16** |
| Mm.33729 | Slc25a22 | Solute carrier family 25 (mitochondrial carrier, glutamate), member 22 | **1.50 ± 0.16** |
| Mm.26135 | Cab39 | Calcium binding protein 39 | **1.49 ± 0.10** |
| Mm.41339 | Myo5c | Myosin VC | **1.44 ± 0.16** |
| Mm.12553 | Rfc3 | Replication factor C (activator 1) 3 | **1.40 ± 0.23** |
| Mm.186185 | Prdx6 | Peroxiredoxin 6 | **1.36 ± 0.05** |
| Mm.442861 | H2-D4 | Histocompatibility 2, D region locus 4 | **1.36 ± 0.16** |
| Mm.26614 | Mrpl30 | Mitochondrial ribosomal protein L30 | **1.35 ± 0.19** |
| Mm.74610 | Fam46a | Family with sequence similarity 46, member A | **1.30 ± 0.15** |
| Mm.154378 | Ncl | Nucleolin | **1.27 ± 0.22** |
| Mm.290774 | Hspa8 | Heat shock protein 8 | **1.26 ± 0.29** |
| Mm.30085 | Akr1a4 | Aldo-keto reductase family 1, member A4 (aldehyde reductase) | **1.26 ± 0.27** |
| Mm.235398 | Cwc27 | CWC27 spliceosome-associated protein homolog | **1.20 ± 0.11** |
| Mm.157534 | Prpf38a | PRP38 pre-mRNA processing factor 38 | **1.20 ± 0.14** |
| Mm.238343 | Anxa2 | Annexin A2 | **1.18 ± 0.17** |
| Mm.757 | Rhoa | Ras homolog gene family, member A | **1.18 ± 0.21** |
| Mm.6856 | Pttg1 | Pituitary tumor-transforming gene 1 | **1.17 ± 0.16** |
| Mm.284248 | Ccl5 | Chemokine (C-C motif) ligand 5 | **1.17 ± 0.27** |
| Mm.172720 | Lrrc59 | Leucine rich repeat containing 59 | **1.16 ± 0.12** |
| Mm.209419 | Hspa9 | Heat shock protein 9 | **1.14 ± 0.14** |
| Mm.377926 | Mrgprb3 | MAS-related GPR, member B3 | **1.11 ± 0.18** |
| Mm.291358 | Gm5218 | PREDICTED: predicted gene 5218 | **1.11 ± 0.24** |
| Mm.283045 | Med28 | Mediator of RNA polymerase II transcription, subunit 28 homolog | **1.09 ± 0.19** |
| Mm.49665 | Nkain2 | Na+/K+ transporting ATPase interacting 2 | **1.08 ± 0.22** |
| Mm.24521 | Zfand2a | Zinc finger, AN1-type domain 2A | **1.08 ± 0.17** |
| Mm.379366 | Rsl24d1 | Ribosomal L24 domain containing 1 | **1.08 ± 0.10** |
| Mm.263177 | Pdia3 | Protein disulfide isomerase associated 3 | **1.08 ± 0.02** |
| Mm.23748 | Rnf31 | Ring finger protein 31 | **1.07 ± 0.11** |
| Mm.387173 | Chi3l3 | Chitinase 3-like 3 | **1.06 ± 0.20** |
| Mm.198414 | Stk39 | Serine/threonine kinase 39, STE20/SPS1 homolog | **1.06 ± 0.12** |
| Mm.91920 | Socs6 | Suppressor of cytokine signaling 6 | **1.06 ± 0.16** |
| Mm.34587 | Trim26 | Tripartite motif-containing 26 | **1.06 ± 0.10** |
| Mm.380129 | G3bp1 | Ras-GTPase-activating protein SH3-domain binding protein 1 | **1.06 ± 0.08** |
| Mm.443373 | Klk1b5 | Kallikrein 1-related peptidase b5 | **1.04 ± 0.11** |
| Mm.383293 | Hist2h3b | Histone cluster 2, H3b | **1.04 ± 0.20** |
| Mm.151168 | Naa20 | N(alpha)-acetyltransferase 20, NatB catalytic subunit | **1.04 ± 0.14** |
| Mm.249225 | M6pr | Mannose-6-phosphate receptor, cation dependent | **1.02 ± 0.18** |
| Mm.41737 | Gng2 | Guanine nucleotide binding protein (G protein), gamma 2 | **1.01 ± 0.09** |
| Mm.486 | Lamp2 | Lysosomal-associated membrane protein 2 | **1.01 ± 0.22** |
| Mm.37516 | Dnajb11 | Dnaj (Hsp40) homolog, subfamily B, member 11 | **1.01 ± 0.14** |
| Mm.275942 | Arpc3 | Actin related protein 2/3 complex, subunit 3 | **1.01 ± 0.05** |
| Mm.256034 | Cct3 | Chaperonin containing Tcp1, subunit 3 (gamma) | **1.00 ± 0.03** |
| Mm.1777 | Hspd1 | Heat shock protein 1 (chaperonin) | **1.00 ± 0.08** |
| Mm.28520 | Ski | Ski sarcoma viral oncogene homolog | **-1.00 ± 0.19** |
| Mm.2299 | Cd244 | CD244 natural killer cell receptor 2B4 | **-1.00 ± 0.03** |
| Mm.485067 | 4930481A15Rik | RIKEN cDNA 4930481A15 gene | **-1.00 ± 0.15** |
| Mm.29442 | ORF61 | Open reading frame 61 | **-1.01 ± 0.04** |
| Mm.38927 | Ppil4 | Peptidylprolyl isomerase (cyclophilin)-like 4 | **-1.02 ± 0.20** |
| Mm.27090 | Fastkd5 | FAST kinase domains 5 | **-1.02 ± 0.03** |
| Mm.485049 | Map4k4 | Mitogen-activated protein kinase kinase kinase kinase 4 | **-1.04 ± 0.14** |
| Mm.272430 | 1110059E24Rik | RIKEN cDNA 1110059E24 gene | **-1.04 ± 0.11** |
| Mm.158501 | 4921539E11Rik | RIKEN cDNA 4921539E11 gene | **-1.05 ± 0.02** |
| Mm.453372 | Fam36a | Family with sequence similarity 36, member A | **-1.05 ± 0.19** |
| Mm.41063 | Trim24 | Tripartite motif-containing 24 | **-1.05 ± 0.13** |
| Mm.5875 | Prss8 | Protease, serine, 8 | **-1.07 ± 0.10** |
| Mm.268475 | Ccpg1 | Cell cycle progression 1 | **-1.07 ± 0.12** |
| Mm.291824 | Mmgt1 | Membrane magnesium transporter 1 | **-1.07 ± 0.15** |
| Mm.392667 | Zfp148 | Zinc finger protein 148 | **-1.08 ± 0.03** |
| Mm.33910 | Dok3 | Docking protein 3 | **-1.08 ± 0.20** |
| Mm.474129 | C430048L16Rik | RIKEN cDNA C430048L16 gene | **-1.08 ± 0.13** |
| Mm.237095 | Strbp | Spermatid perinuclear RNA binding protein | **-1.09 ± 0.22** |
| Mm.260890 | Fam98c | Family with sequence similarity 98, member C | **-1.10 ± 0.15** |
| Mm.77017 | Rasgrp2 | RAS, guanyl releasing protein 2 | **-1.11 ± 0.16** |
| Mm.485834 | Braf | Braf transforming gene | **-1.12 ± 0.03** |
| Mm.24215 | Arrdc2 | Arrestin domain containing 2 | **-1.12 ± 0.08** |
| Mm.160020 | Cyp4f18 | Cytochrome P450, family 4, subfamily f, polypeptide 18 | **-1.17 ± 0.12** |
| Mm.311655 | Satb1 | Special AT-rich sequence binding protein 1 | **-1.17 ± 0.12** |
| Mm.157119 | Sort1 | Sortilin 1 | **-1.17 ± 0.19** |
| Mm.1 | S100a10 | S100 calcium binding protein A10 (calpactin) | **-1.17 ± 0.14** |
| Mm.250419 | Ccni | Cyclin I | **-1.17 ± 0.08** |
| Mm.267377 | Scd1 | Stearoyl-Coenzyme A desaturase 1 | **-1.17 ± 0.21** |
| Mm.226820 | Thap3 | THAP domain containing, apoptosis associated protein 3 | **-1.17 ± 0.20** |
| Mm.259312 | Sp4 | Trans-acting transcription factor 4 | **-1.19 ± 0.16** |
| Mm.485882 | Rhot2 | Ras homolog gene family, member T2 | **-1.19 ± 0.18** |
| Mm.275138 | Dzip3 | DAZ interacting protein 3, zinc finger | **-1.21 ± 0.17** |
| Mm.377902 | Tas2r102 | Taste receptor, type 2, member 102 | **-1.21 ± 0.24** |
| Mm.29476 | Cdc42se2 | CDC42 small effector 2 | **-1.22 ± 0.28** |
| Mm.3311 | Plbd1 | Phospholipase B domain containing 1 | **-1.22 ± 0.24** |
| Mm.124328 | Sp3 | Trans-acting transcription factor 3 | **-1.22 ± 0.16** |
| Mm.275265 | 2310030N02Rik | RIKEN cDNA 2310030N02 gene | **-1.23 ± 0.07** |
| Mm.340955 | Smurf2 | SMAD specific E3 ubiquitin protein ligase 2 | **-1.24 ± 0.18** |
| Mm.277599 | Stub1 | STIP1 homology and U-Box containing protein 1 | **-1.24 ± 0.23** |
| Mm.212921 | 4930432E11Rik | PREDICTED: RIKEN cDNA 4930432E11 gene | **-1.25 ± 0.12** |
| Mm.330745 | Mafb | V-maf musculoaponeurotic fibrosarcoma oncogene family, protein B | **-1.27 ± 0.12** |
| Mm.210334 | Mbtd1 | Mbt domain containing 1 | **-1.30 ± 0.26** |
| Mm.479516 | Olfr819 | Olfactory receptor 819 | **-1.32 ± 0.17** |
| Mm.485788 | Tm7sf2 | Transmembrane 7 superfamily member 2 | **-1.33 ± 0.22** |
| Mm.27186 | D230025D16Rik | RIKEN cDNA D230025D16 gene | **-1.35 ± 0.06** |
| Mm.29586 | Basp1 | Brain abundant, membrane attached signal protein 1 | **-1.37 ± 0.10** |
| Mm.458106 | Itpr3 | Inositol 1,4,5-triphosphate receptor 3 | **-1.37 ± 0.21** |
| Mm.226899 | Fam13b | Family with sequence similarity 13, member B | **-1.37 ± 0.27** |
| Mm.38199 | Btla | B and T lymphocyte associated | **-1.37 ± 0.27** |
| Mm.390371 | Hsf5 | Heat shock transcription factor family member 5 | **-1.39 ± 0.31** |
| Mm.260193 | Arrb1 | Arrestin, beta 1 | **-1.39 ± 0.12** |
| Mm.132299 | Tmem71 | Transmembrane protein 71 | **-1.40 ± 0.17** |
| Mm.478284 | Dst | Dystonin | **-1.41 ± 0.28** |
| Mm.390986 | Dhx16 | DEAH (Asp-Glu-Ala-His) box polypeptide 16 | **-1.42 ± 0.19** |
| Mm.119274 | Prrxl1 | Paired related homeobox protein-like 1 | **-1.44 ± 0.13** |
| Mm.483321 | Bmp10 | Bone morphogenetic protein 10 | **-1.45 ± 0.23** |
| Mm.439723 | Rev3l | REV3-like, catalytic subunit of DNA polymerase zeta RAD54 like | **-1.46 ± 0.18** |
| Mm.250256 | Enpp2 | Ectonucleotide pyrophosphatase/phosphodiesterase 2 | **-1.46 ± 0.27** |
| Mm.34641 | Amfr | Autocrine motility factor receptor | **-1.48 ± 0.30** |
| Mm.272368 | Crip1 | Cysteine-rich protein 1 | **-1.48 ± 0.32** |
| Mm.288671 | Acap1 | ArfGAP with coiled-coil, ankyrin repeat and PH domains 1 | **-1.48 ± 0.23** |
| Mm.121920 | Sorl1 | Sortilin-related receptor, LDLR class A repeats-containing | **-1.52 ± 0.27** |
| Mm.355614 | Pde7a | Phosphodiesterase 7A | **-1.53 ± 0.14** |
| Mm.35059 | Arhgap5 | Rho GTPase activating protein 5 | **-1.54 ± 0.22** |
| Mm.439850 | Cdadc1 | Cytidine and dCMP deaminase domain containing 1 | **-1.55 ± 0.30** |
| Mm.474531 | Rbm26 | RNA binding motif protein 26 | **-1.55 ± 0.31** |
| Mm.400802 | Cacng2 | Calcium channel, voltage-dependent, gamma subunit 2 | **-1.56 ± 0.04** |
| Mm.31417 | Zmym2 | Zinc finger, MYM-type 2 | **-1.57 ± 0.09** |
| Mm.336117 | Xlr3c | X-linked lymphocyte-regulated 3C | **-1.58 ± 0.32** |
| Mm.292739 | Polr3gl | Polymerase (RNA) III (DNA directed) polypeptide G like | **-1.60 ± 0.14** |
| Mm.223420 | Olfr711 | Olfactory receptor 711 | **-1.61 ± 0.16** |
| Mm.293811 | Rb1cc1 | RB1-inducible coiled-coil 1 | **-1.61 ± 0.35** |
| Mm.151129 | Cc2d1b | Coiled-coil and C2 domain containing 1B | **-1.62 ± 0.21** |
| Mm.29820 | Bnip3l | BCL2/adenovirus E1B interacting protein 3-like | **-1.65 ± 0.34** |
| Mm.269088 | Anp32a | Acidic (leucine-rich) nuclear phosphoprotein 32 family, member A | **-1.67 ± 0.34** |
| Mm.289796 | Lins | Lines homolog | **-1.68 ± 0.37** |
| Mm.215641 | Nagpa | N-acetylglucosamine-1-phosphodiester alpha-N-acetylglucosaminidase | **-1.69 ± 0.16** |
| Mm.213582 | Chst15 | Carbohydrate (N-acetylgalactosamine 4-sulfate 6-O) sulfotransferase 15 | **-1.69 ± 0.23** |
| Mm.345095 | Col20a1 | Collagen, type XX, alpha 1 | **-1.70 ± 0.29** |
| Mm.219475 | Wdr20b | WD repeat domain 20b | **-1.71 ± 0.16** |
| Mm.306228 | St8sia4 | ST8 alpha-N-acetyl-neuraminide alpha-2,8-sialyltransferase 4 | **-1.73 ± 0.35** |
| Mm.57734 | Lims1 | LIM and senescent cell antigen-like domains 1 | **-1.74 ± 0.32** |
| Mm.475704 | Tsr2 | TSR2, 20S rRNA accumulation, homolog | **-1.75 ± 0.33** |
| Mm.458815 | Ccl21a | Chemokine (C-C motif) ligand 21A (serine) | **-1.80 ± 0.28** |
| Mm.3925 | S100a4 | S100 calcium binding protein A4 | **-1.81 ± 0.25** |
| Mm.268896 | Erich1 | Glutamate-rich 1 | **-1.82 ± 0.24** |
| Mm.25709 | Ing1 | Inhibitor of growth family, member 1 | **-1.83 ± 0.33** |
| Mm.333026 | Tcrb-J | T-cell receptor beta, joining region | **-1.83 ± 0.21** |
| Mm.485838 | Vps37a | Vacuolar protein sorting 37A | **-1.84 ± 0.41** |
| Mm.327439 | AA792892 | Expressed sequence AA792892 | **-1.85 ± 0.23** |
| Mm.127166 | Prss34 | Protease, serine, 34 | **-1.89 ± 0.13** |
| Mm.37426 | Cd163 | CD163 antigen | **-1.90 ± 0.23** |
| Mm.443529 | Arhgap20 | Rho GTPase activating protein 20 | **-1.90 ± 0.12** |
| Mm.34608 | Clybl | Citrate lyase beta like | **-1.92 ± 0.24** |
| Mm.390829 | Mtbp | Mdm2, transformed 3T3 cell double minute p53 binding protein | **-1.94 ± 0.44** |
| Mm.296789 | Entpd7 | Ectonucleoside triphosphate diphosphohydrolase 7 | **-2.01 ± 0.03** |
| Mm.261676 | Hist1h2bc | Histone cluster 1, h2bc | **-2.02 ± 0.30** |
| Mm.336054 | Gm595 | Predicted gene 595 | **-2.03 ± 0.26** |
| Mm.31567 | Ankrd11 | Ankyrin repeat domain 11 | **-2.03 ± 0.39** |
| Mm.244068 | Arhgef7 | Rho guanine nucleotide exchange factor (GEF7) | **-2.08 ± 0.36** |
| Mm.391556 | Madcam1 | Mucosal vascular addressin cell adhesion molecule 1 | **-2.10 ± 0.46** |
| Mm.52711 | 0610030E20Rik | RIKEN cDNA 0610030E20 gene | **-2.13 ± 0.42** |
| Mm.26908 | Csnk1a1 | Casein kinase 1, alpha 1 | **-2.16 ± 0.41** |
| Mm.138792 | Chd7 | Chromodomain helicase DNA binding protein 7 | **-2.22 ± 0.11** |
| Mm.281452 | P2ry1 | Purinergic receptor P2Y, G-protein coupled 1 | **-2.25 ± 0.40** |
| Mm.252987 | Slc12a5 | Solute carrier family 12, member 5 | **-2.25 ± 0.30** |
| Mm.480575 | Sdpr | Serum deprivation response | **-2.26 ± 0.27** |
| Mm.41868 | 2810405K02Rik | RIKEN cDNA 2810405K02 gene | **-2.28 ± 0.18** |
| Mm.213406 | Uggt2 | UDP-glucose glycoprotein glucosyltransferase 2 | **-2.32 ± 0.16** |
| Mm.485040 | Trib2 | Tribbles homolog 2 | **-2.33 ± 0.40** |
| Mm.296181 | Hspa2 | Heat shock protein 2 | **-2.33 ± 0.37** |
| Mm.1239 | Gfap | Glial fibrillary acidic protein | **-2.35 ± 0.25** |
| Mm.319117 | Ptpn12 | Protein tyrosine phosphatase, non-receptor type 12 | **-2.37 ± 0.13** |
| Mm.439929 | 2810001A02Rik | PREDICTED: RIKEN cDNA 2810001A02 gene | **-2.37 ± 0.21** |
| Mm.202665 | Rnase4 | Ribonuclease, Rnase A family 4 | **-2.48 ± 0.17** |
| Mm.274926 | Emb | Embigin | **-2.62 ± 0.22** |
| Mm.482512 | 1700094D03Rik | RIKEN cDNA 1700094D03 gene | **-2.66 ± 0.06** |
| Mm.247775 | Hook2 | Hook homolog 2 | **-2.75 ± 0.50** |
| Mm.35650 | Tspan31 | Tetraspanin 31 | **-2.81 ± 0.25** |
| Mm.73234 | Cep72 | Centrosomal protein 72 | **-2.87 ± 0.30** |
| Mm.217354 | Mrps6 | Mitochondrial ribosomal protein S6 | **-2.89 ± 0.59** |
| Mm.260103 | Cep164 | Centrosomal protein 164 | **-3.08 ± 0.40** |
| Mm.273997 | Ppp2r2a | Protein phosphatase 2 (formerly 2A), regulatory subunit B (PR 52), alpha isoform | **-3.09 ± 0.40** |
| Mm.181836 | Mphosph6 | M phase phosphoprotein 6 | **-3.09 ± 0.44** |
| Mm.194536 | Gmfg | Glia maturation factor, gamma | **-3.21 ± 0.46** |
| Mm.443244 | Fam71b | Family with sequence similarity 71, member B | **-3.44 ± 0.57** |
| Mm.203965 | Cand1 | Cullin associated and neddylation disassociated 1 | **-3.78 ± 0.42** |
| Mm.18344 | Psmc3ip | Proteasome (prosome, macropain) 26S subunit, ATPase 3, interacting protein | **-3.93 ± 0.74** |
| Mm.246858 | Sprn | Shadow of prion protein | **-3.98 ± 0.47** |
| Mm.43358 | Pbx1 | Pre B-cell leukemia transcription factor 1 | **-3.99 ± 0.59** |
| Mm.34330 | Nnat | Neuronatin | **-4.29 ± 0.28** |
| Mm.271724 | Dtx3 | Deltex 3 homolog | **-4.43 ± 0.71** |
| Mm.34002 | 2410015M20Rik | RIKEN cDNA 2410015M20 gene | **-4.46 ± 0.20** |
| Mm.276696 | Armc3 | Armadillo repeat containing 3 | **-4.52 ± 0.74** |
| Mm.155877 | Ulk3 | Unc-51-like kinase 3 | **-4.68 ± 0.53** |
| Mm.440026 | Ilf3 | Interleukin enhancer binding factor 3 | **-4.72 ± 0.51** |
| Mm.483877 | Usp44 | Ubiquitin specific peptidase 44 | **-4.82 ± 0.46** |
| Mm.485146 | Sipa1l3 | Signal-induced proliferation-associated 1 like 3 | **-5.05 ± 0.78** |
| Mm.258300 | Agpat4 | 1-acylglycerol-3-phosphate O-acyltransferase 4 | **-5.26 ± 0.57** |
| Mm.19987 | Dct | Dopachrome tautomerase | **-5.41 ± 0.72** |
| Mm.131237 | Stap1 | Signal transducing adaptor family member 1 | **-5.43 ± 0.90** |
| Mm.484399 | Sycp2l | PREDICTED: synaptonemal complex protein 2-like | **-5.45 ± 0.49** |
| Mm.2581 | Epha2 | Eph receptor A2 | **-5.68 ± 0.56** |
| Mm.186936 | Rbfa | Ribosome binding factor A | **-5.75 ± 0.57** |
| Mm.441431 | Syn2 | Synapsin II | **-5.85 ± 1.11** |
| Mm.22842 | Cd2 | CD2 antigen | **-6.04 ± 0.39** |
| Mm.89943 | Adam18 | A disintegrin and metallopeptidase domain 18 | **-6.25 ± 0.48** |
| Mm.377086 | Hoxa13 | Homeobox A13 | **-6.36 ± 0.60** |
| **Genes uniquely modulated at 48 h pi** | | | |
| Mm.141021 | Ifitm3 | Interferon induced transmembrane protein 3 | **2.86 ± 0.05** |
| Mm.247453 | Abhd10 | Abhydrolase domain containing 10 | **2.78 ± 0.26** |
| Mm.485634 | Fpr2 | Formyl peptide receptor 2 | **2.75 ± 0.13** |
| Mm.41075 | Drd4 | Dopamine receptor D4 | **2.64 ± 0.19** |
| Mm.482320 | H2-Q8 | Histocompatibility 2, Q region locus 8 | **2.61 ± 0.04** |
| Mm.139695 | Epn2 | Epsin 2 | **2.49 ± 0.16** |
| Mm.439661 | Gp49a | Glycoprotein 49 A | **2.40 ± 0.13** |
| Mm.377402 | Olfr1511 | Olfactory receptor 1511 | **2.38 ± 0.19** |
| Mm.243758 | Apol9a | Apolipoprotein L 9a | **2.38 ± 0.17** |
| Mm.479935 | Pydc4 | Pyrin domain containing 4 | **2.32 ± 0.51** |
| Mm.231288 | Retnlg | Resistin like gamma | **2.31 ± 0.36** |
| Mm.426537 | LOC100041885 | PREDICTED: sp110 nuclear body protein-like | **2.28 ± 0.11** |
| Mm.484214 | LOC100503803 | PREDICTED: uncharacterized protein c2orf16-like | **2.19 ± 0.19** |
| Mm.175661 | Ifitm1 | Interferon induced transmembrane protein 1 | **2.14 ± 0.39** |
| Mm.131422 | Osm | Oncostatin M | **2.12 ± 0.34** |
| Mm.277377 | Trim30d | Tripartite motif-containing 30D | **2.04 ± 0.09** |
| Mm.2082 | Apod | Apolipoprotein D | **2.04 ± 0.23** |
| Mm.272746 | Cmtm2a | CKLF-like MARVEL transmembrane domain containing 2A | **2.03 ± 0.10** |
| Mm.431282 | H2-Q9 | Histocompatibility 2, Q region locus 9 | **1.92 ± 0.17** |
| Mm.290774 | Hspa8 | Heat shock protein 8 | **1.84 ± 0.22** |
| Mm.234441 | Lmcd1 | LIM and cysteine-rich domains 1 | **1.82 ± 0.25** |
| Mm.1114 | Gla | Galactosidase, alpha | **1.79 ± 0.08** |
| Mm.219497 | Xylb | Xylulokinase homolog | **1.77 ± 0.16** |
| Mm.293120 | Stat2 | Signal transducer and activator of transcription 2 | **1.74 ± 0.30** |
| Mm.156919 | Ctsz | Cathepsin Z | **1.70 ± 0.28** |
| Mm.439648 | H2-T23 | Histocompatibility 2, T region locus 23 | **1.61 ± 0.26** |
| Mm.249645 | Jak3 | Janus kinase 3 | **1.59 ± 0.09** |
| Mm.482288 | Ctu2 | Cytosolic thiouridylase subunit 2 homolog | **1.58 ± 0.16** |
| Mm.244406 | Parp14 | Poly (ADP-ribose) polymerase family, member 14 | **1.56 ± 0.17** |
| Mm.290390 | Ms4a6d | Membrane-spanning 4-domains, subfamily A, member 6D | **1.56 ± 0.19** |
| Mm.28231 | Rarres2 | Retinoic acid receptor responder (tazarotene induced) 2 | **1.54 ± 0.09** |
| Mm.379266 | Ifitm2 | Interferon induced transmembrane protein 2 | **1.54 ± 0.15** |
| Mm.441101 | Clec4d | C-type lectin domain family 4, member d | **1.53 ± 0.29** |
| Mm.271745 | Nrp1 | Neuropilin 1 | **1.53 ± 0.18** |
| Mm.20488 | Trafd1 | TRAF type zinc finger domain containing 1 | **1.53 ± 0.07** |
| Mm.18803 | Timm22 | Translocase of inner mitochondrial membrane 22 homolog | **1.50 ± 0.15** |
| Mm.130 | Socs1 | Suppressor of cytokine signaling 1 | **1.47 ± 0.03** |
| Mm.317779 | LOC100042025 | PREDICTED: glyceraldehyde-3-phosphate dehydrogenase-like, transcript variant 2 | **1.44 ± 0.23** |
| Mm.294020 | Aspscr1 | Alveolar soft part sarcoma chromosome region, candidate 1 | **1.40 ± 0.12** |
| Mm.971 | Plg | Plasminogen | **1.39 ± 0.25** |
| Mm.19931 | Slc10a3 | Solute carrier family 10 (sodium/bile acid cotransporter family), member 3 | **1.39 ± 0.01** |
| Mm.21642 | Spic | Spi-C transcription factor (Spi-1/PU.1 related) | **1.39 ± 0.22** |
| Mm.28385 | Tmem176b | Transmembrane protein 176B | **1.37 ± 0.01** |
| Mm.337931 | Fcrlb | Fc receptor-like B | **1.36 ± 0.15** |
| Mm.286753 | Rgs3 | Regulator of G-protein signaling 3 | **1.35 ± 0.12** |
| Mm.15819 | Cd68 | CD68 antigen | **1.33 ± 0.17** |
| Mm.107718 | H1fnt | H1 histone family, member N, testis-specific | **1.32 ± 0.14** |
| Mm.259969 | Lysmd3 | Lysm, putative peptidoglycan-binding, domain containing 3 | **1.29 ± 0.06** |
| Mm.190 | Xcl1 | Chemokine (C motif) ligand 1 | **1.28 ± 0.08** |
| Mm.377805 | Olfr679 | Olfactory receptor 679 | **1.25 ± 0.13** |
| Mm.40548 | Nmnat2 | Nicotinamide nucleotide adenylyltransferase 2 | **1.24 ± 0.17** |
| Mm.276669 | Rab38 | RAB38, member of RAS oncogene family | **1.23 ± 0.14** |
| Mm.290502 | Aida | Axin interactor, dorsalization associated | **1.23 ± 0.23** |
| Mm.2923 | Il2rg | Interleukin 2 receptor, gamma chain | **1.23 ± 0.23** |
| Mm.26614 | Mrpl30 | Mitochondrial ribosomal protein L30 | **1.22 ± 0.25** |
| Mm.29430 | Arl6ip4 | ADP-ribosylation factor-like 6 interacting protein 4 | **1.21 ± 0.15** |
| Mm.485049 | Map4k4 | Mitogen-activated protein kinase kinase kinase kinase 4 | **1.20 ± 0.10** |
| Mm.102286 | Gabra3 | Gamma-aminobutyric acid (GABA) A receptor, subunit alpha 3 | **1.20 ± 0.19** |
| Mm.2645 | Eef1a2 | Eukaryotic translation elongation factor 1 alpha 2 | **1.20 ± 0.19** |
| Mm.134863 | Agtr1b | Angiotensin II receptor, type 1b | **1.19 ± 0.18** |
| Mm.107483 | Nr2c1 | Nuclear receptor subfamily 2, group C, member 1 | **1.18 ± 0.08** |
| Mm.398371 | Rpl7a | Ribosomal protein L7A | **1.17 ± 0.19** |
| Mm.136791 | Svil | Supervillin | **1.17 ± 0.09** |
| Mm.391871 | Fbp2 | Fructose bisphosphatase 2 | **1.16 ± 0.21** |
| Mm.98 | Psmb6 | Proteasome (prosome, macropain) subunit, beta type 6 | **1.15 ± 0.09** |
| Mm.181021 | Col4a2 | Collagen, type IV, alpha 2 | **1.14 ± 0.25** |
| Mm.38055 | Esd | Esterase D/formylglutathione hydrolase | **1.13 ± 0.07** |
| Mm.485161 | Slc15a3 | Solute carrier family 15, member 3 | **1.13 ± 0.11** |
| Mm.386456 | A530032D15Rik | RIKEN cDNA A530032D15Rik gene | **1.13 ± 0.21** |
| Mm.20273 | Urm1 | Ubiquitin related modifier 1 homolog | **1.12 ± 0.12** |
| Mm.3507 | Nr4a2 | Nuclear receptor subfamily 4, group A, member 2 | **1.12 ± 0.21** |
| Mm.193040 | Mrps7 | Mitchondrial ribosomal protein S7 | **1.11 ± 0.15** |
| Mm.297768 | Arf4 | ADP-ribosylation factor 4 | **1.11 ± 0.16** |
| Mm.288960 | Mrpl47 | Mitochondrial ribosomal protein L47 | **1.10 ± 0.17** |
| Mm.280103 | Atp1a1 | ATPase, Na+/K+ transporting, alpha 1 polypeptide | **1.10 ± 0.16** |
| Mm.295031 | Ddx27 | DEAD (Asp-Glu-Ala-Asp) box polypeptide 27 | **1.10 ± 0.06** |
| Mm.439656 | Cebpb | CCAAT/enhancer binding protein (C/EBP), beta | **1.10 ± 0.08** |
| Mm.213003 | Myd88 | Myeloid differentiation primary response gene 88 | **1.10 ± 0.12** |
| Mm.63984 | Mitd1 | MIT, microtubule interacting and transport, domain containing 1 | **1.09 ± 0.19** |
| Mm.359633 | Ctsa | Cathepsin A | **1.08 ± 0.08** |
| Mm.196013 | Samd9l | Sterile alpha motif domain containing 9-like | **1.08 ± 0.18** |
| Mm.237935 | Etl4 | Enhancer trap locus 4 | **1.07 ± 0.17** |
| Mm.46029 | Gpatch4 | G patch domain containing 4 | **1.07 ± 0.01** |
| Mm.259620 | Usp42 | Ubiquitin specific peptidase 42 | **1.06 ± 0.23** |
| Mm.103810 | Tacr3 | Tachykinin receptor 3 | **1.06 ± 0.19** |
| Mm.101885 | Dpf1 | D4, zinc and double PHD fingers family 1 | **1.05 ± 0.15** |
| Mm.29490 | Ctdsp2 | CTD (carboxy-terminal domain, RNA polymerase II, polypeptide A) small phosphatase 2 | **1.04 ± 0.14** |
| Mm.9537 | Lcn2 | Lipocalin 2 | **1.04 ± 0.21** |
| Mm.377699 | Olfr620 | Olfactory receptor 620 | **1.04 ± 0.19** |
| Mm.371557 | Eif4a1 | Eukaryotic translation initiation factor 4A1 | **1.03 ± 0.14** |
| Mm.274393 | Rd3 | Retinal degeneration 3 | **1.03 ± 0.20** |
| Mm.248778 | Ndufa10 | NADH dehydrogenase (ubiquinone) 1 alpha subcomplex 10 | **1.03 ± 0.07** |
| Mm.390068 | Zfpm1 | Zinc finger protein, multitype 1 | **1.02 ± 0.07** |
| Mm.68971 | Dlg5 | Discs, large homolog 5 | **1.01 ± 0.09** |
| Mm.319719 | Rpl13 | Ribosomal protein L13 | **1.01 ± 0.04** |
| Mm.3935 | Ddx24 | DEAD (Asp-Glu-Ala-Asp) box polypeptide 24 | **1.01 ± 0.12** |
| Mm.307720 | 1700026J12Rik | PREDICTED: RIKEN cDNA 1700026J12 gene | **1.00 ± 0.14** |
| Mm.21687 | Limd2 | LIM domain containing 2 | **-1.00 ± 0.09** |
| Mm.389459 | Bglap | Bone gamma carboxyglutamate protein | **-1.01 ± 0.15** |
| Mm.244549 | Slc6a9 | Solute carrier family 6 (neurotransmitter transporter, glycine), member 9 | **-1.01 ± 0.16** |
| Mm.439653 | Nr2f1 | Nuclear receptor subfamily 2, group F, member 1 | **-1.02 ± 0.05** |
| Mm.27503 | 1810009A15Rik | RIKEN cDNA 1810009A15 gene | **-1.04 ± 0.06** |
| Mm.246625 | Kir3dl1 | Killer cell immunoglobulin-like receptor, three domains, long cytoplasmic tail, 1 | **-1.06 ± 0.15** |
| Mm.67366 | Zfp317 | Zinc finger protein 317 | **-1.07 ± 0.13** |
| Mm.373589 | Dcbld2 | Discoidin, CUB and LCCL domain containing 2 | **-1.07 ± 0.24** |
| Mm.19355 | Rpl35a | Ribosomal protein L35A | **-1.08 ± 0.22** |
| Mm.127166 | Prss34 | Protease, serine, 34 | **-1.09 ± 0.13** |
| Mm.142822 | Ewsr1 | Ewing sarcoma breakpoint region 1 | **-1.09 ± 0.15** |
| Mm.193212 | Hyi | Hydroxypyruvate isomerase homolog | **-1.09 ± 0.09** |
| Mm.645 | Stim1 | Stromal interaction molecule 1 | **-1.10 ± 0.04** |
| Mm.142095 | Carhsp1 | Calcium regulated heat stable protein 1 | **-1.11 ± 0.22** |
| Mm.6877 | Ap2a1 | Adaptor protein complex AP-2, alpha 1 subunit | **-1.12 ± 0.21** |
| Mm.425285 | Iqcd | IQ motif containing D | **-1.12 ± 0.13** |
| Mm.379345 | Sidt2 | SID1 transmembrane family, member 2 | **-1.12 ± 0.18** |
| Mm.258771 | Ptprs | Protein tyrosine phosphatase, receptor type, S | **-1.13 ± 0.13** |
| Mm.221688 | Phip | Pleckstrin homology domain interacting protein | **-1.16 ± 0.23** |
| Mm.485838 | Vps37a | Vacuolar protein sorting 37A | **-1.16 ± 0.23** |
| Mm.28528 | Rwdd1 | RWD domain containing 1 | **-1.18 ± 0.23** |
| Mm.276325 | Sod1 | Superoxide dismutase 1, soluble | **-1.18 ± 0.22** |
| Mm.351553 | Cnot2 | CCR4-NOT transcription complex, subunit 2 | **-1.18 ± 0.19** |
| Mm.246688 | Ezh2 | Enhancer of zeste homolog 2 | **-1.18 ± 0.20** |
| Mm.485040 | Trib2 | Tribbles homolog 2 | **-1.18 ± 0.07** |
| Mm.213566 | Hs6st1 | Heparan sulfate 6-O-sulfotransferase 1 | **-1.19 ± 0.24** |
| Mm.28262 | Rgs2 | Regulator of G-protein signaling 2 | **-1.20 ± 0.14** |
| Mm.29842 | Ndufv1 | NADH dehydrogenase (ubiquinone) flavoprotein 1 | **-1.22 ± 0.20** |
| Mm.441077 | BC002059 | CDNA sequence BC002059 | **-1.22 ± 0.22** |
| Mm.379181 | 1700020I14Rik | RIKEN cDNA 1700020I14 gene | **-1.23 ± 0.08** |
| Mm.271559 | Defb39 | Defensin beta 39 | **-1.28 ± 0.09** |
| Mm.205190 | Mll5 | Myeloid/lymphoid or mixed-lineage leukemia 5 | **-1.29 ± 0.08** |
| Mm.34706 | Ankrd12 | Ankyrin repeat domain 12 | **-1.29 ± 0.23** |
| Mm.234587 | Rufy2 | RUN and FYVE domain-containing 2 | **-1.30 ± 0.26** |
| Mm.3624 | Guk1 | Guanylate kinase 1 | **-1.31 ± 0.28** |
| Mm.34955 | Chd2 | Chromodomain helicase DNA binding protein 2 | **-1.32 ± 0.26** |
| Mm.256025 | Faah | Fatty acid amide hydrolase | **-1.35 ± 0.15** |
| Mm.272093 | Crlf3 | Cytokine receptor-like factor 3 | **-1.36 ± 0.11** |
| Mm.246440 | Tm9sf3 | Transmembrane 9 superfamily member 3 | **-1.36 ± 0.26** |
| Mm.390076 | Nwd1 | NACHT and WD repeat domain containing 1 | **-1.39 ± 0.26** |
| Mm.333124 | Igk | Immunoglobulin kappa chain complex | **-1.39 ± 0.08** |
| Mm.390180 | Uchl4 | Ubiquitin carboxyl-terminal esterase L4 | **-1.39 ± 0.27** |
| Mm.398119 | Oaz1 | Ornithine decarboxylase antizyme 1 | **-1.40 ± 0.19** |
| Mm.74704 | Zc3h13 | Zinc finger CCCH type containing 13 | **-1.42 ± 0.16** |
| Mm.292834 | Pard6b | Par-6 (partitioning defective 6) homolog beta | **-1.45 ± 0.33** |
| Mm.17484 | Snca | Synuclein, alpha | **-1.46 ± 0.30** |
| Mm.432511 | Raver1 | Ribonucleoprotein, PTB-binding 1 | **-1.46 ± 0.31** |
| Mm.34641 | Amfr | Autocrine motility factor receptor | **-1.46 ± 0.31** |
| Mm.4658 | Cdh3 | Cadherin 3 | **-1.48 ± 0.25** |
| Mm.458621 | Pappa2 | Pappalysin 2 | **-1.53 ± 0.22** |
| Mm.458815 | Ccl21a | Chemokine (C-C motif) ligand 21A (serine) | **-1.55 ± 0.10** |
| Mm.332844 | Cyp3a11 | Cytochrome P450, family 3, subfamily a, polypeptide 11 | **-1.56 ± 0.18** |
| Mm.456234 | Iglv1 | Immunoglobulin lambda variable 1 | **-1.58 ± 0.33** |
| Mm.407493 | Ccl21c | Chemokine (C-C motif) ligand 21C (leucine) | **-1.58 ± 0.08** |
| Mm.479516 | Olfr819 | Olfactory receptor 819 | **-1.62 ± 0.33** |
| Mm.358682 | BC051019 | CDNA sequence BC051019 | **-1.63 ± 0.11** |
| Mm.89646 | Lace1 | Lactation elevated 1 | **-1.64 ± 0.28** |
| Mm.268475 | Ccpg1 | Cell cycle progression 1 | **-1.65 ± 0.28** |
| Mm.65906 | Pbxip1 | Pre-B-cell leukemia transcription factor interacting protein 1 | **-1.69 ± 0.25** |
| Mm.383203 | Nit2 | Nitrilase family, member 2 | **-1.70 ± 0.15** |
| Mm.246119 | Dync2li1 | Dynein cytoplasmic 2 light intermediate chain 1 | **-1.74 ± 0.20** |
| Mm.331893 | Hmgb4 | High-mobility group box 4 | **-1.78 ± 0.11** |
| Mm.54120 | Samd4b | Sterile alpha motif domain containing 4B | **-1.82 ± 0.25** |
| Mm.82274 | Kalrn | Kalirin, RhoGEF kinase | **-1.85 ± 0.40** |
| Mm.439662 | Ebf1 | Early B-cell factor 1 | **-1.87 ± 0.20** |
| Mm.4662 | Irg1 | Immunoresponsive gene 1 | **-1.90 ± 0.37** |
| Mm.111326 | Cdk2 | Cyclin-dependent kinase 2 | **-1.91 ± 0.24** |
| Mm.281452 | P2ry1 | Purinergic receptor P2Y, G-protein coupled 1 | **-1.96 ± 0.06** |
| Mm.458468 | Kif14 | Kinesin family member 14 | **-2.01 ± 0.38** |
| Mm.252830 | Odf1 | Outer dense fiber of sperm tails 1 | **-2.04 ± 0.22** |
| Mm.250866 | Aldh1a1 | Aldehyde dehydrogenase family 1, subfamily A1 | **-2.04 ± 0.28** |
| Mm.246595 | Hcrtr1 | Hypocretin (orexin) receptor 1 | **-2.06 ± 0.38** |
| Mm.23670 | Fastkd3 | FAST kinase domains 3 | **-2.10 ± 0.16** |
| Mm.27366 | Gramd1a | GRAM domain containing 1A | **-2.24 ± 0.45** |
| Mm.368256 | Gm9758 | Predicted gene 9758 | **-2.24 ± 0.51** |
| Mm.107441 | Zfp26 | Zinc finger protein 26 | **-2.37 ± 0.05** |
| Mm.130787 | Btnl9 | Butyrophilin-like 9 | **-2.73 ± 0.40** |
| Mm.252987 | Slc12a5 | Solute carrier family 12, member 5 | **-2.75 ± 0.32** |
| Mm.271724 | Dtx3 | Deltex 3 homolog | **-3.01 ± 0.65** |
| Mm.196158 | Kcnk10 | Potassium channel, subfamily K, member 10 | **-3.06 ± 0.06** |
| Mm.389816 | Gm4745 | Predicted gene 4745 | **-3.11 ± 0.29** |
| Mm.358731 | Cnot3 | CCR4-NOT transcription complex, subunit 3 | **-3.14 ± 0.33** |
| Mm.11350 | Zbtb42 | Zinc finger and BTB domain containing 42 | **-3.21 ± 0.24** |
| Mm.423023 | Ahsp | Alpha hemoglobin stabilizing protein | **-3.28 ± 0.54** |
| Mm.440164 | Hbb-y | Hemoglobin Y, beta-like embryonic chain | **-3.35 ± 0.58** |
| Mm.296789 | Entpd7 | Ectonucleoside triphosphate diphosphohydrolase 7 | **-3.49 ± 0.14** |
| Mm.203965 | Cand1 | Cullin associated and neddylation disassociated 1 | **-3.53 ± 0.24** |
| Mm.60688 | Ccdc70 | Coiled-coil domain containing 70 | **-3.60 ± 0.63** |
| Mm.258300 | Agpat4 | 1-acylglycerol-3-phosphate O-acyltransferase 4 | **-3.93 ± 0.76** |
| Mm.234204 | Pak2 | P21 protein (Cdc42/Rac)-activated kinase 2 | **-3.94 ± 0.63** |
| Mm.19987 | Dct | Dopachrome tautomerase | **-3.94 ± 0.09** |
| Mm.484399 | Sycp2l | PREDICTED: synaptonemal complex protein 2-like | **-3.98 ± 0.47** |
| Mm.233914 | Ccdc64 | Coiled-coil domain containing 64 | **-3.98 ± 0.32** |
| Mm.441431 | Syn2 | Synapsin II | **-4.19 ± 0.32** |
| Mm.28839 | Eif2b1 | Eukaryotic translation initiation factor 2B, subunit 1 (alpha) | **-4.46 ± 0.51** |
| Mm.27949 | Sh3rf1 | SH3 domain containing ring finger 1 | **-5.07 ± 0.70** |
| Mm.2581 | Epha2 | Eph receptor A2 | **-5.09 ± 0.45** |
| Mm.255063 | Wrb | Tryptophan rich basic protein | **-5.36 ± 0.40** |
| **Genes uniquely modulated at 72 h pi** | | | |
| Mm.317779 | LOC100042025 | PREDICTED: glyceraldehyde-3-phosphate dehydrogenase-like, transcript variant 2 | **2.26 ± 0.20** |
| Mm.333011 | Gm2663 | Predicted gene 2663 | **2.23 ± 0.24** |
| Mm.485478 | Trim30b | Tripartite motif-containing 30B | **2.18 ± 0.32** |
| Mm.4950 | Isg15 | ISG15 ubiquitin-like modifier | **2.16 ± 0.46** |
| Mm.256414 | Slc9a2 | Solute carrier family 9 (sodium/hydrogen exchanger), member 2 | **2.13 ± 0.24** |
| Mm.15510 | Gzma | Granzyme A | **2.04 ± 0.22** |
| Mm.475107 | Rtp4 | Receptor transporter protein 4 | **1.89 ± 0.28** |
| Mm.482724 | Gm13102 | Predicted gene 13102 | **1.87 ± 0.20** |
| Mm.32912 | Psmb1 | Proteasome (prosome, macropain) subunit, beta type 1 | **1.85 ± 0.32** |
| Mm.98232 | Plek | Pleckstrin | **1.85 ± 0.24** |
| Mm.426670 | Ssr1 | Signal sequence receptor, alpha | **1.82 ± 0.29** |
| Mm.233009 | Rap1b | RAS related protein 1b | **1.78 ± 0.31** |
| Mm.7454 | Igbp1 | Immunoglobulin (CD79A) binding protein 1 | **1.78 ± 0.10** |
| Mm.215667 | Hspe1 | Heat shock protein 1 (chaperonin 10) | **1.78 ± 0.26** |
| Mm.302516 | Fsip2 | PREDICTED: fibrous sheath-interacting protein 2 | **1.77 ± 0.30** |
| Mm.323595 | Tob2 | Transducer of ERBB2, 2 | **1.76 ± 0.34** |
| Mm.236795 | Mrpl13 | Mitochondrial ribosomal protein L13 | **1.73 ± 0.34** |
| Mm.3254 | Zfp787 | Zinc finger protein 787 | **1.66 ± 0.33** |
| Mm.441542 | Gstp2 | Glutathione S-transferase, pi 2 | **1.65 ± 0.36** |
| Mm.40548 | Nmnat2 | Nicotinamide nucleotide adenylyltransferase 2 | **1.61 ± 0.04** |
| Mm.29778 | Manf | Mesencephalic astrocyte-derived neurotrophic factor | **1.57 ± 0.27** |
| Mm.398371 | Rpl7a | Ribosomal protein L7A | **1.57 ± 0.30** |
| Mm.222228 | Cks2 | CDC28 protein kinase regulatory subunit 2 | **1.56 ± 0.27** |
| Mm.288753 | Igk-V38 | Immunoglobulin kappa chain variable 38(V38) | **1.53 ± 0.21** |
| Mm.34102 | Odc1 | Ornithine decarboxylase, structural 1 | **1.52 ± 0.34** |
| Mm.180191 | Psmb8 | Proteasome (prosome, macropain) subunit, beta type 8 | **1.52 ± 0.32** |
| Mm.24038 | Gbp2 | Guanylate binding protein 2 | **1.50 ± 0.16** |
| Mm.485049 | Map4k4 | Mitogen-activated protein kinase kinase kinase kinase 4 | **1.49 ± 0.17** |
| Mm.439648 | H2-T23 | Histocompatibility 2, T region locus 23 | **1.49 ± 0.23** |
| Mm.318846 | Camta1 | Calmodulin binding transcription activator 1 | **1.49 ± 0.26** |
| Mm.335338 | 4922501C03Rik | RIKEN cDNA 4922501C03 gene | **1.47 ± 0.15** |
| Mm.258204 | Ybx1 | Y box protein 1 | **1.47 ± 0.29** |
| Mm.130 | Socs1 | Suppressor of cytokine signaling 1 | **1.46 ± 0.20** |
| Mm.441366 | Gm5662 | Predicted gene 5662 | **1.46 ± 0.29** |
| Mm.390374 | C1qtnf2 | C1q and tumor necrosis factor related protein 2 | **1.45 ± 0.31** |
| Mm.57243 | Hrh2 | Histamine receptor H2 | **1.45 ± 0.28** |
| Mm.36501 | Mtap1a | Microtubule-associated protein 1 A | **1.44 ± 0.19** |
| Mm.235287 | Ranbp1 | RAN binding protein 1 | **1.44 ± 0.12** |
| Mm.917 | Inpp1 | Inositol polyphosphate-1-phosphatase | **1.42 ± 0.29** |
| Mm.1360 | Gadd45b | Growth arrest and DNA-damage-inducible 45 beta | **1.40 ± 0.10** |
| Mm.192991 | Mt1 | Metallothionein 1 | **1.38 ± 0.28** |
| Mm.126106 | Fancc | Fanconi anemia, complementation group C | **1.37 ± 0.19** |
| Mm.442861 | H2-D4 | Histocompatibility 2, D region locus 4 | **1.37 ± 0.27** |
| Mm.332901 | Ppp1r9a | Protein phosphatase 1, regulatory (inhibitor) subunit 9A | **1.37 ± 0.29** |
| Mm.4454 | Elk3 | ELK3, member of ETS oncogene family | **1.36 ± 0.15** |
| Mm.322186 | Rasl2-9-ps | RAS-like, family 2, locus 9, pseudogene | **1.35 ± 0.24** |
| Mm.29430 | Arl6ip4 | ADP-ribosylation factor-like 6 interacting protein 4 | **1.35 ± 0.14** |
| Mm.341608 | Rffl | Ring finger and FYVE like domain containing protein | **1.33 ± 0.25** |
| Mm.196827 | Krtap16-1 | Keratin associated protein 16-1 | **1.33 ± 0.24** |
| Mm.77325 | Nkpd1 | Ntpase, KAP family P-loop domain containing 1 | **1.32 ± 0.28** |
| Mm.4298 | En2 | Engrailed 2 | **1.30 ± 0.14** |
| Mm.291358 | Gm5218 | PREDICTED: predicted gene 5218 | **1.30 ± 0.23** |
| Mm.221314 | Hist1h1b | Histone cluster 1, H1b | **1.28 ± 0.10** |
| Mm.7491 | Nmi | N-myc (and STAT) interactor | **1.28 ± 0.22** |
| Mm.448624 | Ccnb3 | Cyclin B3 | **1.28 ± 0.19** |
| Mm.200611 | Spna1 | Spectrin alpha 1 | **1.27 ± 0.25** |
| Mm.377926 | Mrgprb3 | MAS-related GPR, member B3 | **1.26 ± 0.05** |
| Mm.386757 | Clcn1 | Chloride channel 1 | **1.26 ± 0.26** |
| Mm.8003 | Mc5r | Melanocortin 5 receptor | **1.26 ± 0.28** |
| Mm.87628 | 2510012J08Rik | RIKEN cDNA 2510012J08 gene | **1.26 ± 0.17** |
| Mm.440339 | Trpm3 | Transient receptor potential cation channel, subfamily M, member 3 | **1.25 ± 0.20** |
| Mm.1777 | Hspd1 | Heat shock protein 1 (chaperonin) | **1.23 ± 0.15** |
| Mm.377407 | Olfr1394 | Olfactory receptor 1394 | **1.23 ± 0.24** |
| Mm.479523 | Gm3448 | Predicted gene 3448 | **1.23 ± 0.27** |
| Mm.45377 | 1700010M22Rik | RIKEN cDNA 1700010M22 gene | **1.22 ± 0.25** |
| Mm.726 | Bsg | Basigin | **1.22 ± 0.17** |
| Mm.292056 | Acsl5 | Acyl-coA synthetase long-chain family member 5 | **1.22 ± 0.11** |
| Mm.358632 | Rpl30 | Ribosomal protein L30 | **1.21 ± 0.19** |
| Mm.472798 | 2010015L04Rik | RIKEN cDNA 2010015L04 gene | **1.21 ± 0.21** |
| Mm.831 | Ssr4 | Signal sequence receptor, delta | **1.20 ± 0.22** |
| Mm.180182 | Cox5b | Cytochrome c oxidase, subunit Vb | **1.19 ± 0.25** |
| Mm.170276 | Dusp4 | Dual specificity phosphatase 4 | **1.18 ± 0.26** |
| Mm.24108 | Mrps21 | Mitochondrial ribosomal protein S21 | **1.17 ± 0.21** |
| Mm.482288 | Ctu2 | Cytosolic thiouridylase subunit 2 homolog | **1.16 ± 0.10** |
| Mm.259969 | Lysmd3 | Lysm, putative peptidoglycan-binding, domain containing 3 | **1.16 ± 0.12** |
| Mm.93796 | Mcf2 | Mcf.2 transforming sequence | **1.15 ± 0.17** |
| Mm.24848 | Deb1 | Differentially expressed in B16F10 1 | **1.15 ± 0.10** |
| Mm.223330 | Olfr807 | Olfactory receptor 807 | **1.14 ± 0.26** |
| Mm.208883 | Psma5 | Proteasome (prosome, macropain) subunit, alpha type 5 | **1.13 ± 0.04** |
| Mm.359511 | Gm16390 | Predicted gene 16390 | **1.13 ± 0.14** |
| Mm.297440 | Ran | RAN, member RAS oncogene family | **1.13 ± 0.11** |
| Mm.121485 | Sfxn5 | Sideroflexin 5 | **1.13 ± 0.23** |
| Mm.263177 | Pdia3 | Protein disulfide isomerase associated 3 | **1.13 ± 0.14** |
| Mm.26540 | Zfp943 | Zinc finger protein 943 | **1.12 ± 0.08** |
| Mm.439686 | Svs6 | Seminal vesicle secretory protein 6 | **1.11 ± 0.16** |
| Mm.391871 | Fbp2 | Fructose bisphosphatase 2 | **1.11 ± 0.11** |
| Mm.389956 | Olfr309 | Olfactory receptor 309 | **1.11 ± 0.17** |
| Mm.86671 | Rimbp3 | RIMS binding protein 3 | **1.10 ± 0.15** |
| Mm.246967 | Alms1 | Alstrom syndrome 1 homolog | **1.10 ± 0.01** |
| Mm.115804 | Olfr877 | Olfactory receptor 877 | **1.10 ± 0.25** |
| Mm.10229 | Srsf10 | Serine/arginine-rich splicing factor 10 | **1.10 ± 0.18** |
| Mm.390868 | 1810013L24Rik | RIKEN cDNA 1810013L24 gene | **1.10 ± 0.19** |
| Mm.99576 | Ovch2 | Ovochymase 2 | **1.09 ± 0.08** |
| Mm.340818 | Atp6v0a1 | ATPase, H+ transporting, lysosomal V0 subunit A1 | **1.09 ± 0.17** |
| Mm.245739 | Trub2 | Trub pseudouridine (psi) synthase homolog 2 | **1.09 ± 0.19** |
| Mm.158903 | Hlf | Hepatic leukemia factor | **1.09 ± 0.19** |
| Mm.43081 | Mapk8ip3 | Mitogen-activated protein kinase 8 interacting protein 3 | **1.09 ± 0.20** |
| Mm.902 | 1100001G20Rik | RIKEN cDNA 1100001G20 gene | **1.09 ± 0.17** |
| Mm.265834 | Slc8a1 | Solute carrier family 8 (sodium/calcium exchanger), member 1 | **1.09 ± 0.09** |
| Mm.339542 | Obscn | Obscurin, cytoskeletal calmodulin and titin-interacting RhoGEF | **1.09 ± 0.21** |
| Mm.249005 | Ltn1 | Listerin E3 ubiquitin protein ligase 1 | **1.09 ± 0.04** |
| Mm.2050 | Rpl15 | Ribosomal protein L15 | **1.07 ± 0.23** |
| Mm.228797 | Mvp | Major vault protein | **1.07 ± 0.14** |
| Mm.25585 | Mrps18c | Mitochondrial ribosomal protein S18C | **1.07 ± 0.23** |
| Mm.273098 | Agrn | Agrin | **1.07 ± 0.18** |
| Mm.476028 | Mdm4 | Transformed mouse 3T3 cell double minute 4 | **1.07 ± 0.23** |
| Mm.212763 | Chmp5 | Chromatin modifying protein 5 | **1.07 ± 0.21** |
| Mm.277792 | Col1a2 | Collagen, type I, alpha 2 | **1.07 ± 0.20** |
| Mm.291442 | Sparc | Secreted acidic cysteine rich glycoprotein | **1.07 ± 0.21** |
| Mm.107718 | H1fnt | H1 histone family, member N, testis-specific | **1.07 ± 0.23** |
| Mm.21353 | Dnajc11 | Dnaj (Hsp40) homolog, subfamily C, member 11 | **1.06 ± 0.18** |
| Mm.267478 | Acsl6 | Acyl-coA synthetase long-chain family member 6 | **1.05 ± 0.20** |
| Mm.272969 | Spc25 | SPC25, NDC80 kinetochore complex component, homolog | **1.05 ± 0.19** |
| Mm.332735 | Gtf2ird1 | General transcription factor II I repeat domain-containing 1 | **1.05 ± 0.22** |
| Mm.32588 | Dusp18 | Dual specificity phosphatase 18 | **1.05 ± 0.19** |
| Mm.88795 | H2-Q10 | Histocompatibility 2, Q region locus 10 | **1.04 ± 0.05** |
| Mm.2266 | Apoh | Apolipoprotein H | **1.04 ± 0.12** |
| Mm.379375 | Hnrnpa3 | Heterogeneous nuclear ribonucleoprotein A3 | **1.04 ± 0.20** |
| Mm.284491 | 3200002M19Rik | RIKEN cDNA 3200002M19 gene | **1.03 ± 0.12** |
| Mm.371583 | Slpi | Secretory leukocyte peptidase inhibitor | **1.02 ± 0.21** |
| Mm.223188 | Olfr414 | Olfactory receptor 414 | **1.01 ± 0.14** |
| Mm.284719 | Dcdc2a | Doublecortin domain containing 2a | **1.01 ± 0.14** |
| Mm.335249 | Ppib | Peptidylprolyl isomerase B | **1.01 ± 0.20** |
| Mm.350894 | Rhox2g | Reproductive homeobox 2G | **1.01 ± 0.16** |
| Mm.1403 | Myo7a | Myosin VIIA | **1.01 ± 0.13** |
| Mm.274784 | Hnrnph3 | Heterogeneous nuclear ribonucleoprotein H3 | **1.01 ± 0.17** |
| Mm.377828 | Olfr547 | Olfactory receptor 547 | **1.01 ± 0.11** |
| Mm.485945 | 2610203C22Rik | RIKEN cDNA 2610203C22 gene | **1.00 ± 0.06** |
| Mm.29722 | Usmg5 | Upregulated during skeletal muscle growth 5 | **1.00 ± 0.12** |
| Mm.261679 | Rps26 | Ribosomal protein S26 | **-1.00 ± 0.18** |
| Mm.256422 | Sf1 | Splicing factor 1 | **-1.00 ± 0.17** |
| Mm.42146 | Tlr6 | Toll-like receptor 6 | **-1.00 ± 0.06** |
| Mm.252145 | Ckmt1 | Creatine kinase, mitochondrial 1, ubiquitous | **-1.01 ± 0.05** |
| Mm.260084 | Eif4a2 | Eukaryotic translation initiation factor 4A2 | **-1.01 ± 0.18** |
| Mm.330057 | Bmyc | Brain expressed myelocytomatosis oncogene | **-1.01 ± 0.17** |
| Mm.334648 | Cd97 | CD97 antigen | **-1.02 ± 0.13** |
| Mm.335096 | Pvrl1 | Poliovirus receptor-related 1 | **-1.02 ± 0.03** |
| Mm.27900 | 1810013D10Rik | RIKEN cDNA 1810013D10 gene | **-1.03 ± 0.19** |
| Mm.17958 | Slc50a1 | Solute carrier family 50 (sugar transporter), member 1 | **-1.05 ± 0.22** |
| Mm.138321 | Gpatch2 | G patch domain containing 2 | **-1.05 ± 0.08** |
| Mm.398119 | Oaz1 | Ornithine decarboxylase antizyme 1 | **-1.05 ± 0.15** |
| Mm.52297 | Fnbp1 | Formin binding protein 1 | **-1.05 ± 0.18** |
| Mm.234003 | Lsp1 | Lymphocyte specific 1 | **-1.05 ± 0.21** |
| Mm.17009 | Ralbp1 | Rala binding protein 1 | **-1.06 ± 0.02** |
| Mm.245938 | Cwc15 | CWC15 homolog | **-1.07 ± 0.12** |
| Mm.322870 | Vps33b | Vacuolar protein sorting 33B | **-1.08 ± 0.24** |
| Mm.377875 | Hist1h4f | Histone cluster 1, H4f | **-1.08 ± 0.11** |
| Mm.207496 | Prkcb | Protein kinase C, beta | **-1.08 ± 0.20** |
| Mm.236211 | Thrap3 | Thyroid hormone receptor associated protein 3 | **-1.09 ± 0.24** |
| Mm.340955 | Smurf2 | SMAD specific E3 ubiquitin protein ligase 2 | **-1.09 ± 0.06** |
| Mm.272278 | Mfap4 | Microfibrillar-associated protein 4 | **-1.12 ± 0.08** |
| Mm.100450 | Sptlc3 | Serine palmitoyltransferase, long chain base subunit 3 | **-1.12 ± 0.22** |
| Mm.289109 | Ccdc107 | Coiled-coil domain containing 107 | **-1.13 ± 0.22** |
| Mm.312593 | Serpina1e | Serine (or cysteine) peptidase inhibitor, clade A, member 1E | **-1.13 ± 0.03** |
| Mm.302938 | Ddx3y | DEAD (Asp-Glu-Ala-Asp) box polypeptide 3, Y-linked | **-1.14 ± 0.22** |
| Mm.379094 | Rpl36 | Ribosomal protein L36 | **-1.14 ± 0.12** |
| Mm.439877 | Grcc10 | Gene rich cluster, C10 gene | **-1.15 ± 0.21** |
| Mm.296240 | 2310037I24Rik | RIKEN cDNA 2310037I24 gene | **-1.15 ± 0.14** |
| Mm.482352 | Cstf2t | Cleavage stimulation factor, 3' pre-RNA subunit 2, tau | **-1.16 ± 0.10** |
| Mm.32741 | Itga10 | Integrin, alpha 10 | **-1.17 ± 0.22** |
| Mm.259846 | Zfp711 | Zinc finger protein 711 | **-1.17 ± 0.17** |
| Mm.399829 | Rps3a | Ribosomal protein S3A | **-1.17 ± 0.08** |
| Mm.325816 | Csnk1g1 | Casein kinase 1, gamma 1 | **-1.18 ± 0.19** |
| Mm.143813 | Midn | Midnolin | **-1.20 ± 0.25** |
| Mm.41890 | Use1 | Unconventional SNARE in the ER 1 homolog | **-1.20 ± 0.11** |
| Mm.393405 | Cotl1 | Coactosin-like 1 (Dictyostelium) | **-1.20 ± 0.22** |
| Mm.390756 | Stk24 | Serine/threonine kinase 24 (STE20 homolog, yeast) | **-1.20 ± 0.23** |
| Mm.76400 | Zfp579 | Zinc finger protein 579 | **-1.22 ± 0.23** |
| Mm.291062 | Dkc1 | Dyskeratosis congenita 1, dyskerin homolog | **-1.22 ± 0.26** |
| Mm.348794 | Aym1 | Activator of yeast meiotic promoters 1 | **-1.22 ± 0.25** |
| Mm.246625 | Kir3dl1 | Killer cell immunoglobulin-like receptor, three domains, long cytoplasmic tail, 1 | **-1.22 ± 0.14** |
| Mm.180032 | 4930473A06Rik | RIKEN cDNA 4930473A06 gene | **-1.23 ± 0.28** |
| Mm.141054 | Ankrd40 | Ankyrin repeat domain 40 | **-1.24 ± 0.22** |
| Mm.260943 | Eif5b | Eukaryotic translation initiation factor 5B | **-1.25 ± 0.28** |
| Mm.22126 | Fabp1 | Fatty acid binding protein 1, liver | **-1.25 ± 0.23** |
| Mm.151129 | Cc2d1b | Coiled-coil and C2 domain containing 1B | **-1.26 ± 0.23** |
| Mm.202715 | Sun2 | Sad1 and UNC84 domain containing 2 | **-1.26 ± 0.03** |
| Mm.9684 | Vasp | Vasodilator-stimulated phosphoprotein | **-1.26 ± 0.25** |
| Mm.321047 | Gimap6 | GTPase, IMAP family member 6 | **-1.28 ± 0.23** |
| Mm.223395 | Olfr392 | Olfactory receptor 392 | **-1.29 ± 0.10** |
| Mm.31417 | Zmym2 | Zinc finger, MYM-type 2 | **-1.31 ± 0.13** |
| Mm.377071 | Mrps33 | Mitochondrial ribosomal protein S33 | **-1.32 ± 0.28** |
| Mm.29442 | ORF61 | Open reading frame 61 | **-1.32 ± 0.28** |
| Mm.379181 | 1700020I14Rik | RIKEN cDNA 1700020I14 gene | **-1.33 ± 0.08** |
| Mm.27503 | 1810009A15Rik | RIKEN cDNA 1810009A15 gene | **-1.33 ± 0.29** |
| Mm.331640 | Rod1 | ROD1 regulator of differentiation 1 | **-1.34 ± 0.28** |
| Mm.28796 | Fam108b | Family with sequence similarity 108, member B | **-1.34 ± 0.30** |
| Mm.292530 | Dazap2 | DAZ associated protein 2 | **-1.34 ± 0.29** |
| Mm.13787 | Cp | Ceruloplasmin | **-1.35 ± 0.17** |
| Mm.2180 | Hsp90ab1 | Heat shock protein 90 alpha (cytosolic), class B member 1 | **-1.36 ± 0.26** |
| Mm.21687 | Limd2 | LIM domain containing 2 | **-1.37 ± 0.20** |
| Mm.305108 | Retsat | Retinol saturase (all trans retinol 13,14 reductase) | **-1.38 ± 0.26** |
| Mm.240066 | Psme4 | Proteasome (prosome, macropain) activator subunit 4 | **-1.39 ± 0.27** |
| Mm.272368 | Crip1 | Cysteine-rich protein 1 | **-1.39 ± 0.13** |
| Mm.24001 | Mef2c | Myocyte enhancer factor 2C | **-1.39 ± 0.32** |
| Mm.326349 | Iglv1 | Immunoglobulin lambda variable 1 | **-1.40 ± 0.18** |
| Mm.29497 | Iscu | Iscu iron-sulfur cluster scaffold homolog | **-1.40 ± 0.13** |
| Mm.90067 | P2ry13 | Purinergic receptor P2Y, G-protein coupled 13 | **-1.40 ± 0.15** |
| Mm.440610 | 4930599N23Rik | PREDICTED: RIKEN cDNA 4930599N23 gene | **-1.41 ± 0.28** |
| Mm.27681 | Adam17 | A disintegrin and metallopeptidase domain 17 | **-1.41 ± 0.32** |
| Mm.422695 | Mup1 | Major urinary protein 1 | **-1.41 ± 0.23** |
| Mm.33360 | Mylk | Myosin, light polypeptide kinase | **-1.42 ± 0.32** |
| Mm.439850 | Cdadc1 | Cytidine and dCMP deaminase domain containing 1 | **-1.42 ± 0.24** |
| Mm.272093 | Crlf3 | Cytokine receptor-like factor 3 | **-1.45 ± 0.31** |
| Mm.485838 | Vps37a | Vacuolar protein sorting 37A | **-1.45 ± 0.20** |
| Mm.158501 | 4921539E11Rik | RIKEN cDNA 4921539E11 gene | **-1.47 ± 0.22** |
| Mm.223420 | Olfr711 | Olfactory receptor 711 | **-1.48 ± 0.11** |
| Mm.133401 | Arid1b | AT rich interactive domain 1B (SWI-like) | **-1.49 ± 0.19** |
| Mm.6710 | Rock1 | Rho-associated coiled-coil containing protein kinase 1 | **-1.49 ± 0.12** |
| Mm.142822 | Ewsr1 | Ewing sarcoma breakpoint region 1 | **-1.50 ± 0.21** |
| Mm.121920 | Sorl1 | Sortilin-related receptor, LDLR class A repeats-containing | **-1.50 ± 0.11** |
| Mm.390201 | Pgm3 | Phosphoglucomutase 3 | **-1.52 ± 0.10** |
| Mm.426094 | Rom1 | Rod outer segment membrane protein 1 | **-1.53 ± 0.31** |
| Mm.5163 | Rit2 | Ras-like without CAAX 2 | **-1.54 ± 0.22** |
| Mm.333026 | Tcrb-J | T-cell receptor beta, joining region | **-1.55 ± 0.09** |
| Mm.203866 | Ahnak | AHNAK nucleoprotein (desmoyokin) | **-1.60 ± 0.30** |
| Mm.275810 | Rps10 | Ribosomal protein S10 | **-1.62 ± 0.33** |
| Mm.319117 | Ptpn12 | Protein tyrosine phosphatase, non-receptor type 12 | **-1.64 ± 0.15** |
| Mm.71913 | Pla2g2d | Phospholipase A2, group IID | **-1.65 ± 0.11** |
| Mm.269088 | Anp32a | Acidic (leucine-rich) nuclear phosphoprotein 32 family, member A | **-1.68 ± 0.31** |
| Mm.368330 | Il13ra2 | Interleukin 13 receptor, alpha 2 | **-1.69 ± 0.34** |
| Mm.293811 | Rb1cc1 | RB1-inducible coiled-coil 1 | **-1.75 ± 0.39** |
| Mm.479516 | Olfr819 | Olfactory receptor 819 | **-1.75 ± 0.31** |
| Mm.439737 | Cd74 | CD74 antigen | **-1.75 ± 0.19** |
| Mm.288179 | Lig1 | Ligase I, DNA, ATP-dependent | **-1.76 ± 0.34** |
| Mm.482140 | Vasn | Vasorin | **-1.76 ± 0.19** |
| Mm.2987 | Cd79b | CD79B antigen | **-1.78 ± 0.29** |
| Mm.280029 | Hes6 | Hairy and enhancer of split 6 | **-1.79 ± 0.40** |
| Mm.4527 | Cd3d | CD3 antigen, delta polypeptide | **-1.80 ± 0.14** |
| Mm.240473 | Klf13 | Kruppel-like factor 13 | **-1.80 ± 0.38** |
| Mm.34641 | Amfr | Autocrine motility factor receptor | **-1.85 ± 0.28** |
| Mm.45436 | Lyz2 | Lysozyme 2 | **-1.88 ± 0.36** |
| Mm.336117 | Xlr3c | X-linked lymphocyte-regulated 3C | **-1.89 ± 0.40** |
| Mm.35369 | Spink5 | Serine peptidase inhibitor, Kazal type 5 | **-1.91 ± 0.26** |
| Mm.443331 | 1810034E14Rik | PREDICTED: RIKEN cDNA 1810034E14 gene | **-1.92 ± 0.33** |
| Mm.19355 | Rpl35a | Ribosomal protein L35A | **-1.93 ± 0.20** |
| Mm.485468 | Baz1a | Bromodomain adjacent to zinc finger domain 1A | **-2.01 ± 0.42** |
| Mm.485040 | Trib2 | Tribbles homolog 2 | **-2.03 ± 0.30** |
| Mm.289796 | Lins | Lines homolog | **-2.03 ± 0.25** |
| Mm.29685 | Dnajc8 | Dnaj (Hsp40) homolog, subfamily C, member 8 | **-2.03 ± 0.35** |
| Mm.327439 | AA792892 | Expressed sequence AA792892 | **-2.11 ± 0.28** |
| Mm.458468 | Kif14 | Kinesin family member 14 | **-2.14 ± 0.42** |
| Mm.643 | Rps15 | Ribosomal protein S15 | **-2.14 ± 0.17** |
| Mm.311655 | Satb1 | Special AT-rich sequence binding protein 1 | **-2.16 ± 0.46** |
| Mm.101836 | 9630033F20Rik | RIKEN cDNA 9630033F20 gene | **-2.20 ± 0.49** |
| Mm.470126 | Hbb-b1 | Hemoglobin, beta adult major chain | **-2.20 ± 0.48** |
| Mm.111904 | Srpk3 | Serine/arginine-rich protein specific kinase 3 | **-2.23 ± 0.28** |
| Mm.482922 | 1700055N04Rik | PREDICTED: RIKEN cDNA 1700055N04 gene, transcript variant 1 | **-2.23 ± 0.24** |
| Mm.390829 | Mtbp | Mdm2, transformed 3T3 cell double minute p53 binding protein | **-2.23 ± 0.49** |
| Mm.306228 | St8sia4 | ST8 alpha-N-acetyl-neuraminide alpha-2,8-sialyltransferase 4 | **-2.27 ± 0.40** |
| Mm.297199 | Samd14 | Sterile alpha motif domain containing 14 | **-2.27 ± 0.16** |
| Mm.3925 | S100a4 | S100 calcium binding protein A4 | **-2.28 ± 0.38** |
| Mm.250508 | Ppcdc | Phosphopantothenoylcysteine decarboxylase | **-2.31 ± 0.35** |
| Mm.139815 | Tcf7l2 | Transcription factor 7-like 2, T-cell specific, HMG-box | **-2.32 ± 0.44** |
| Mm.235960 | Ank3 | Ankyrin 3, epithelial | **-2.33 ± 0.50** |
| Mm.485882 | Rhot2 | Ras homolog gene family, member T2 | **-2.42 ± 0.43** |
| Mm.390986 | Dhx16 | DEAH (Asp-Glu-Ala-His) box polypeptide 16 | **-2.44 ± 0.41** |
| Mm.439653 | Nr2f1 | Nuclear receptor subfamily 2, group F, member 1 | **-2.50 ± 0.55** |
| Mm.54120 | Samd4b | Sterile alpha motif domain containing 4B | **-2.50 ± 0.38** |
| Mm.289795 | Ubap1 | Ubiquitin-associated protein 1 | **-2.51 ± 0.51** |
| Mm.247218 | Mpi | Mannose phosphate isomerase | **-2.51 ± 0.52** |
| Mm.477813 | Fam13c | Family with sequence similarity 13, member C | **-2.52 ± 0.58** |
| Mm.35628 | Fut8 | Fucosyltransferase 8 | **-2.57 ± 0.52** |
| Mm.20377 | Fam167b | Family with sequence similarity 167, member B | **-2.63 ± 0.44** |
| Mm.296181 | Hspa2 | Heat shock protein 2 | **-2.66 ± 0.28** |
| Mm.268896 | Erich1 | Glutamate-rich 1 | **-2.70 ± 0.52** |
| Mm.205190 | Mll5 | Myeloid/lymphoid or mixed-lineage leukemia 5 | **-2.71 ± 0.41** |
| Mm.271559 | Defb39 | Defensin beta 39 | **-2.72 ± 0.48** |
| Mm.439970 | Cox16 | COX16 cytochrome c oxidase assembly homolog | **-2.72 ± 0.44** |
| Mm.271711 | Tagln2 | Transgelin 2 | **-2.73 ± 0.56** |
| Mm.4528 | Lag3 | Lymphocyte-activation gene 3 | **-2.88 ± 0.65** |
| Mm.484620 | Gm340 | PREDICTED: predicted gene 340 | **-2.93 ± 0.35** |
| Mm.391264 | Gm6812 | Predicted gene 6812 | **-2.94 ± 0.42** |
| Mm.373589 | Dcbld2 | Discoidin, CUB and LCCL domain containing 2 | **-3.01 ± 0.62** |
| Mm.227117 | Slc30a10 | Solute carrier family 30, member 10 | **-3.09 ± 0.38** |
| Mm.207907 | 4932438A13Rik | RIKEN cDNA 4932438A13 gene | **-3.10 ± 0.66** |
| Mm.386769 | Nrap | Nebulin-related anchoring protein | **-3.15 ± 0.29** |
| Mm.26908 | Csnk1a1 | Casein kinase 1, alpha 1 | **-3.17 ± 0.44** |
| Mm.484338 | Olfr31 | Olfactory receptor 31 | **-3.17 ± 0.50** |
| Mm.217354 | Mrps6 | Mitochondrial ribosomal protein S6 | **-3.18 ± 0.17** |
| Mm.2766 | Pvalb | Parvalbumin | **-3.21 ± 0.73** |
| Mm.3468 | Socs3 | Suppressor of cytokine signaling 3 | **-3.25 ± 0.09** |
| Mm.259170 | Ubxn2a | UBX domain protein 2A | **-3.26 ± 0.68** |
| Mm.281452 | P2ry1 | Purinergic receptor P2Y, G-protein coupled 1 | **-3.26 ± 0.47** |
| Mm.234965 | Foxp1 | Forkhead box P1 | **-3.27 ± 0.34** |
| Mm.52711 | 0610030E20Rik | RIKEN cDNA 0610030E20 gene | **-3.30 ± 0.46** |
| Mm.35650 | Tspan31 | Tetraspanin 31 | **-3.32 ± 0.18** |
| Mm.194536 | Gmfg | Glia maturation factor, gamma | **-3.47 ± 0.34** |
| Mm.213651 | Gk5 | Glycerol kinase 5 (putative) | **-3.59 ± 0.37** |
| Mm.252987 | Slc12a5 | Solute carrier family 12, member 5 | **-3.67 ± 0.20** |
| Mm.368256 | Gm9758 | Predicted gene 9758 | **-3.83 ± 0.38** |
| Mm.138792 | Chd7 | Chromodomain helicase DNA binding protein 7 | **-3.90 ± 0.47** |
| Mm.247775 | Hook2 | Hook homolog 2 | **-4.18 ± 0.35** |
| Mm.358731 | Cnot3 | CCR4-NOT transcription complex, subunit 3 | **-4.40 ± 0.42** |
| Mm.181836 | Mphosph6 | M phase phosphoprotein 6 | **-4.52 ± 0.42** |
| Mm.173813 | Notch4 | Notch gene homolog 4 | **-4.60 ± 0.93** |
| Mm.196110 | Hba-a2 | Hemoglobin alpha, adult chain 2 | **-4.69 ± 0.65** |
| Mm.246858 | Sprn | Shadow of prion protein | **-4.74 ± 0.61** |
| Mm.447819 | Klk1b1 | Kallikrein 1-related peptidase b1 | **-4.86 ± 0.58** |
| Mm.407493 | Ccl21c | Chemokine (C-C motif) ligand 21C (leucine) | **-4.96 ± 0.43** |
| Mm.34002 | 2410015M20Rik | RIKEN cDNA 2410015M20 gene | **-5.42 ± 0.84** |
| Mm.271724 | Dtx3 | Deltex 3 homolog | **-5.67 ± 0.32** |
| Mm.43358 | Pbx1 | Pre B-cell leukemia transcription factor 1 | **-6.02 ± 0.32** |
| Mm.273997 | Ppp2r2a | Protein phosphatase 2 (formerly 2A), regulatory subunit B (PR 52), alpha isoform | **-7.07 ± 0.83** |
| Mm.186936 | Rbfa | Ribosome binding factor A | **-7.49 ± 0.13** |
