# Supplementary material for: Differential host gene responses from infection with neurovirulent and partially-neurovirulent strains of Venezuelan equine encephalitis virus
Source: BMC Infect Dis. 2017 Apr 26;17:309. doi: 10.1186/s12879-017-2355-3 (PMC5405508; doi:10.1186/s12879-017-2355-3)
Supplement: Supplementary file 5 — Significantly modulated genes in the brain that were unique to V3000 infection. Genes that were modulated only with V3000 infection in the brain were identified. The list summarizes the commonly modulated genes for each time point studied. Values are expressed as average values of (log2) fold expression for each gene over uninfected controls ± standard error mean (SEM). * P ≤ 0.05. (DOCX 38 kb) [file 12879_2017_2355_MOESM5_ESM.docx]

**Additional file 5: Table S4: Significantly modulated genes in brain that were unique to V3000 infection**

| **UniGene** | **Gene** | **Description** | **Log_2_ Exp ± SEM** |
| --- | --- | --- | --- |
| **Genes uniquely modulated at 48 h pi** | | | |
| Mm.7454 | Igbp1 | Immunoglobulin (CD79A) binding protein 1 | **1.39 ± 0.18** |
| Mm.27742 | Tmem106b | Transmembrane protein 106B | **1.36 ± 0.11** |
| Mm.195803 | Nnt | Nicotinamide nucleotide transhydrogenase | **1.34 ± 0.06** |
| Mm.311516 | Olfr124 | Olfactory receptor 124 | **1.32 ± 0.08** |
| Mm.159684 | Tmpo | Thymopoietin | **1.32 ± 0.05** |
| Mm.26636 | Zfp870 | Zinc finger protein 870 | **1.30 ± 0.07** |
| Mm.116687 | Zbp1 | Z-DNA binding protein 1 | **1.28 ± 0.20** |
| Mm.18803 | Timm22 | Translocase of inner mitochondrial membrane 22 homolog | **1.26 ± 0.22** |
| Mm.439648 | H2-T23 | Histocompatibility 2, T region locus 23 | **1.21 ± 0.19** |
| Mm.46029 | Gpatch4 | G patch domain containing 4 | **1.18 ± 0.20** |
| Mm.260137 | Slc2a9 | Solute carrier family 2 (facilitated glucose transporter), member 9 | **1.16 ± 0.09** |
| Mm.30144 | Ctla2b | Cytotoxic T lymphocyte-associated protein 2 beta | **1.15 ± 0.15** |
| Mm.27925 | Dbndd1 | Dysbindin (dystrobrevin binding protein 1) domain containing 1 | **1.15 ± 0.20** |
| Mm.19080 | Gtpbp1 | GTP binding protein 1 | **1.13 ± 0.23** |
| Mm.266871 | Cbl | Casitas B-lineage lymphoma | **1.12 ± 0.15** |
| Mm.160370 | Slc26a5 | Solute carrier family 26, member 5 | **1.11 ± 0.14** |
| Mm.21353 | Dnajc11 | Dnaj (Hsp40) homolog, subfamily C, member 11 | **1.10 ± 0.08** |
| Mm.386931 | Ecm2 | Extracellular matrix protein 2, female organ and adipocyte specific | **1.10 ± 0.16** |
| Mm.30010 | Arpc1b | Actin related protein 2/3 complex, subunit 1B | **1.09 ± 0.20** |
| Mm.220224 | Gria2 | Glutamate receptor, ionotropic, AMPA2 (alpha 2) | **1.09 ± 0.08** |
| Mm.113942 | 2300009A05Rik | RIKEN cDNA 2300009A05 gene | **1.08 ± 0.13** |
| Mm.90450 | Myo1a | Myosin IA | **1.07 ± 0.16** |
| Mm.209491 | Fnbp1l | Formin binding protein 1-like | **1.05 ± 0.19** |
| Mm.283283 | Tagln | Transgelin | **1.04 ± 0.10** |
| Mm.377863 | Olfr623 | Olfactory receptor 623 | **1.01 ± 0.13** |
| Mm.29357 | Isyna1 | Myo-inositol 1-phosphate synthase A1 | **-1.01 ± 0.14** |
| Mm.378964 | Maz | MYC-associated zinc finger protein (purine-binding transcription factor) | **-1.02 ± 0.10** |
| Mm.260039 | Mettl14 | Methyltransferase like 14 | **-1.02 ± 0.22** |
| Mm.435492 | 1110006G14Rik | PREDICTED: RIKEN cDNA 1110006G14 gene | **-1.02 ± 0.17** |
| Mm.4662 | Irg1 | Immunoresponsive gene 1 | **-1.03 ± 0.22** |
| Mm.274466 | Parp8 | Poly (ADP-ribose) polymerase family, member 8 | **-1.04 ± 0.13** |
| Mm.479529 | D230025D16Rik | RIKEN cDNA D230025D16 gene | **-1.04 ± 0.23** |
| Mm.193212 | Hyi | Hydroxypyruvate isomerase homolog | **-1.05 ± 0.14** |
| Mm.234769 | Dpp3 | Dipeptidylpeptidase 3 | **-1.06 ± 0.22** |
| Mm.293605 | Trp53inp2 | Transformation related protein 53 inducible nuclear protein 2 | **-1.06 ± 0.14** |
| Mm.292729 | Traf6 | TNF receptor-associated factor 6 | **-1.07 ± 0.15** |
| Mm.34201 | Psors1c2 | Psoriasis susceptibility 1 candidate 2 | **-1.08 ± 0.05** |
| Mm.98 | Psmb6 | Proteasome (prosome, macropain) subunit, beta type 6 | **-1.08 ± 0.23** |
| Mm.240252 | Trim14 | Tripartite motif-containing 14 | **-1.08 ± 0.04** |
| Mm.250256 | Enpp2 | Ectonucleotide pyrophosphatase/phosphodiesterase 2 | **-1.09 ± 0.06** |
| Mm.250425 | Fam192a | Family with sequence similarity 192, member A | **-1.09 ± 0.08** |
| Mm.3644 | Fabp7 | Fatty acid binding protein 7, brain | **-1.09 ± 0.04** |
| Mm.38816 | Wdr36 | WD repeat domain 36 | **-1.13 ± 0.17** |
| Mm.285685 | Cd164l2 | CD164 sialomucin-like 2 | **-1.13 ± 0.22** |
| Mm.478815 | Mrps24 | Mitochondrial ribosomal protein S24 | **-1.13 ± 0.16** |
| Mm.26908 | Csnk1a1 | Casein kinase 1, alpha 1 | **-1.16 ± 0.05** |
| Mm.475704 | Tsr2 | TSR2, 20S rRNA accumulation, homolog | **-1.16 ± 0.15** |
| Mm.330408 | Phf21a | PHD finger protein 21A | **-1.17 ± 0.16** |
| Mm.80484 | Usp40 | Ubiquitin specific peptidase 40 | **-1.17 ± 0.26** |
| Mm.297584 | Gcnt4 | Glucosaminyl (N-acetyl) transferase 4, core 2 (beta-1,6-N-acetylglucosaminyltransferase) | **-1.19 ± 0.23** |
| Mm.223420 | Olfr711 | Olfactory receptor 711 | **-1.20 ± 0.12** |
| Mm.185890 | Zdhhc22 | Zinc finger, DHHC-type containing 22 | **-1.21 ± 0.15** |
| Mm.290868 | Tom1 | Target of myb1 homolog | **-1.21 ± 0.09** |
| Mm.2364 | Prtn3 | Proteinase 3 | **-1.23 ± 0.14** |
| Mm.1514 | Lpl | Lipoprotein lipase | **-1.23 ± 0.12** |
| Mm.41540 | Atxn7l1 | Ataxin 7-like 1 | **-1.24 ± 0.06** |
| Mm.41449 | Rdm1 | RAD52 motif 1 | **-1.26 ± 0.26** |
| Mm.250428 | Cypt2 | Cysteine-rich perinuclear theca 2 | **-1.26 ± 0.05** |
| Mm.347413 | Dbil5 | Diazepam binding inhibitor-like 5 | **-1.26 ± 0.01** |
| Mm.470939 | A530064D06Rik | RIKEN cDNA A530064D06 gene | **-1.27 ± 0.23** |
| Mm.25939 | 4930579J09Rik | RIKEN cDNA 4930579J09 gene | **-1.28 ± 0.01** |
| Mm.234667 | Il20ra | Interleukin 20 receptor, alpha | **-1.29 ± 0.04** |
| Mm.296838 | 5730409E04Rik | RIKEN cDNA 5730409E04Rik gene | **-1.29 ± 0.04** |
| Mm.210875 | Dlc1 | Deleted in liver cancer 1 | **-1.30 ± 0.12** |
| Mm.391556 | Madcam1 | Mucosal vascular addressin cell adhesion molecule 1 | **-1.31 ± 0.03** |
| Mm.276769 | Xrcc6bp1 | XRCC6 binding protein 1 | **-1.34 ± 0.27** |
| Mm.202665 | Rnase4 | Ribonuclease, rnase A family 4 | **-1.35 ± 0.14** |
| Mm.292690 | Zfp30 | Zinc finger protein 30 | **-1.36 ± 0.23** |
| Mm.27853 | Exosc5 | Exosome component 5 | **-1.39 ± 0.18** |
| Mm.122538 | Usp31 | Ubiquitin specific peptidase 31 | **-1.40 ± 0.19** |
| Mm.29945 | Edf1 | Endothelial differentiation-related factor 1 | **-1.45 ± 0.16** |
| Mm.259886 | Lmbrd2 | LMBR1 domain containing 2 | **-1.45 ± 0.08** |
| Mm.13787 | Cp | Ceruloplasmin | **-1.46 ± 0.14** |
| Mm.138792 | Chd7 | Chromodomain helicase DNA binding protein 7 | **-1.48 ± 0.20** |
| Mm.112977 | Eepd1 | Endonuclease/exonuclease/phosphatase family domain containing 1 | **-1.49 ± 0.18** |
| Mm.73234 | Cep72 | Centrosomal protein 72 | **-1.50 ± 0.17** |
| Mm.439970 | Cox16 | COX16 cytochrome c oxidase assembly homolog | **-1.52 ± 0.11** |
| Mm.35650 | Tspan31 | Tetraspanin 31 | **-1.54 ± 0.09** |
| Mm.153039 | Mpdz | Multiple PDZ domain protein | **-1.56 ± 0.09** |
| Mm.274926 | Emb | Embigin | **-1.60 ± 0.08** |
| Mm.103413 | Mul1 | Mitochondrial ubiquitin ligase activator of NFKB 1 | **-1.69 ± 0.11** |
| Mm.217354 | Mrps6 | Mitochondrial ribosomal protein S6 | **-1.79 ± 0.29** |
| Mm.458215 | Shf | Src homology 2 domain containing F | **-1.80 ± 0.03** |
| Mm.196158 | Kcnk10 | Potassium channel, subfamily K, member 10 | **-1.84 ± 0.02** |
| Mm.34137 | 1700055N04Rik | PREDICTED: RIKEN cDNA 1700055N04 gene, transcript variant 1 | **-1.87 ± 0.28** |
| Mm.155877 | Ulk3 | Unc-51-like kinase 3 | **-1.88 ± 0.34** |
| Mm.276331 | Usp44 | Ubiquitin specific peptidase 44 | **-1.97 ± 0.16** |
| Mm.137 | Ccl6 | Chemokine (C-C motif) ligand 6 | **-1.97 ± 0.07** |
| Mm.479524 | Nnat | Neuronatin | **-2.05 ± 0.14** |
| Mm.368256 | Gm9758 | Predicted gene 9758 | **-2.06 ± 0.17** |
| Mm.115970 | Adamts16 | A disintegrin-like and metallopeptidase (reprolysin type) with thrombospondin type 1 motif, 16 | **-2.18 ± 0.23** |
| Mm.203965 | Cand1 | Cullin associated and neddylation disassociated 1 | **-2.19 ± 0.40** |
| Mm.248081 | Zufsp | Zinc finger with UFM1-specific peptidase domain | **-2.23 ± 0.36** |
| Mm.196110 | Hba-a2 | Hemoglobin alpha, adult chain 2 | **-2.38 ± 0.32** |
| Mm.273997 | Ppp2r2a | Protein phosphatase 2 (formerly 2A), regulatory subunit B | **-2.51 ± 0.22** |
| Mm.32074 | Kcnn1 | Potassium intermediate/small conductance calcium-activated channel, subfamily N, member 1 | **-2.52 ± 0.13** |
| Mm.276696 | Armc3 | Armadillo repeat containing 3 | **-2.55 ± 0.31** |
| Mm.447819 | Klk1b1 | Kallikrein 1-related peptidase b1 | **-2.64 ± 0.07** |
| Mm.314779 | LOC637277 | PREDICTED: similar to 145 kDa nucleolar protein | **-2.70 ± 0.18** |
| Mm.472701 | Meis3 | Meis homeobox 3 | **-2.83 ± 0.48** |
| Mm.233914 | Ccdc64 | Coiled-coil domain containing 64 | **-2.88 ± 0.17** |
| Mm.60688 | Ccdc70 | Coiled-coil domain containing 70 | **-2.93 ± 0.35** |
| Mm.288567 | Hbb-b1 | Hemoglobin, beta adult major chain | **-3.07 ± 0.43** |
| Mm.194986 | Dgkg | Diacylglycerol kinase, gamma | **-3.08 ± 0.41** |
| Mm.22842 | Cd2 | CD2 antigen | **-3.19 ± 0.73** |
| Mm.475174 | Far2 | Fatty acyl coa reductase 2 | **-3.21 ± 0.40** |
| Mm.12834 | Lfng | LFNG O-fucosylpeptide 3-beta-N-acetylglucosaminyltransferase | **-3.34 ± 0.76** |
| Mm.89943 | Adam18 | A disintegrin and metallopeptidase domain 18 | **-3.44 ± 0.63** |
| Mm.26768 | Spdef | SAM pointed domain containing ets transcription factor | **-3.46 ± 0.59** |
| Mm.30533 | Gapt | Grb2-binding adaptor, transmembrane | **-3.50 ± 0.47** |
| Mm.255063 | Wrb | Tryptophan rich basic protein | **-4.08 ± 0.89** |
| Mm.415 | Iapp | Islet amyloid polypeptide | **-4.64 ± 0.77** |
| **Genes uniquely modulated at 72 h pi** | | | |
| Mm.102312 | Cyp2g1 | Cytochrome P450, family 2, subfamily g, polypeptide 1 | **1.42 ± 0.06** |
| Mm.788 | Ly6e | Lymphocyte antigen 6 complex, locus E | **1.28 ± 0.05** |
| Mm.243758 | Apol9a | Apolipoprotein L 9a | **1.22 ± 0.24** |
| Mm.439648 | H2-T23 | Histocompatibility 2, T region locus 23 | **1.10 ± 0.19** |
| Mm.311516 | Olfr124 | Olfactory receptor 124 | **1.09 ± 0.18** |
| Mm.41529 | Igdcc3 | Immunoglobulin superfamily, DCC subclass, member 3 | **1.04 ± 0.13** |
| Mm.41339 | Myo5c | Myosin VC | **1.01 ± 0.22** |
| Mm.7800 | Itpr2 | Inositol 1,4,5-triphosphate receptor 2 | **-1.00 ± 0.20** |
| Mm.439850 | Cdadc1 | Cytidine and dCMP deaminase domain containing 1 | **-1.00 ± 0.12** |
| Mm.215641 | Nagpa | N-acetylglucosamine-1-phosphodiester alpha-N-acetylglucosaminidase | **-1.01 ± 0.14** |
| Mm.331893 | Hmgb4 | High-mobility group box 4 | **-1.02 ± 0.15** |
| Mm.29945 | Edf1 | Endothelial differentiation-related factor 1 | **-1.02 ± 0.18** |
| Mm.29628 | B3gnt1 | UDP-GlcNAc:betagal beta-1,3-N-acetylglucosaminyltransferase 1 | **-1.02 ± 0.14** |
| Mm.90067 | P2ry13 | Purinergic receptor P2Y, G-protein coupled 13 | **-1.05 ± 0.13** |
| Mm.389058 | Cdk6 | Cyclin-dependent kinase 6 | **-1.08 ± 0.24** |
| Mm.131530 | Camk2a | Calcium/calmodulin-dependent protein kinase II alpha | **-1.11 ± 0.06** |
| Mm.478120 | Cadps2 | Ca2+-dependent activator protein for secretion 2 | **-1.13 ± 0.20** |
| Mm.181836 | Mphosph6 | M phase phosphoprotein 6 | **-1.16 ± 0.10** |
| Mm.332967 | Ube2h | Ubiquitin-conjugating enzyme E2H | **-1.17 ± 0.24** |
| Mm.307239 | Jmjd5 | Jumonji domain containing 5 | **-1.17 ± 0.20** |
| Mm.271711 | Tagln2 | Transgelin 2 | **-1.18 ± 0.23** |
| Mm.289796 | Lins2 | Lines homolog 2 | **-1.20 ± 0.14** |
| Mm.49942 | Tmem177 | Transmembrane protein 177 | **-1.24 ± 0.16** |
| Mm.234314 | Fam131b | Family with sequence similarity 131, member B | **-1.25 ± 0.22** |
| Mm.223420 | Olfr711 | Olfactory receptor 711 | **-1.26 ± 0.11** |
| Mm.256718 | Gne | Glucosamine | **-1.27 ± 0.19** |
| Mm.221403 | Pdgfra | Platelet derived growth factor receptor, alpha polypeptide | **-1.28 ± 0.12** |
| Mm.260039 | Mettl14 | Methyltransferase like 14 | **-1.37 ± 0.15** |
| Mm.286285 | Phf17 | PHD finger protein 17 | **-1.37 ± 0.16** |
| Mm.122538 | Usp31 | Ubiquitin specific peptidase 31 | **-1.38 ± 0.29** |
| Mm.387073 | Unc79 | Unc-79 homolog | **-1.39 ± 0.24** |
| Mm.41449 | Rdm1 | RAD52 motif 1 | **-1.39 ± 0.28** |
| Mm.35650 | Tspan31 | Tetraspanin 31 | **-1.39 ± 0.01** |
| Mm.35806 | Crct1 | Cysteine-rich C-terminal 1 | **-1.39 ± 0.18** |
| Mm.214178 | Gm597 | Predicted gene 597 | **-1.52 ± 0.28** |
| Mm.478856 | Dnaja4 | Dnaj (Hsp40) homolog, subfamily A, member 4 | **-1.54 ± 0.17** |
| Mm.25939 | 4930579J09Rik | RIKEN cDNA 4930579J09 gene | **-1.61 ± 0.24** |
| Mm.190616 | Vgll1 | Vestigial like 1 homolog | **-1.66 ± 0.21** |
| Mm.55847 | Spag17 | Sperm associated antigen 17 | **-1.67 ± 0.11** |
| Mm.41540 | Atxn7l1 | Ataxin 7-like 1 | **-1.71 ± 0.17** |
| Mm.4558 | Yes1 | Yamaguchi sarcoma viral (v-yes) oncogene homolog 1 | **-1.72 ± 0.29** |
| Mm.259464 | Pick1 | Protein interacting with C kinase 1 | **-1.75 ± 0.25** |
| Mm.259886 | Lmbrd2 | LMBR1 domain containing 2 | **-1.79 ± 0.11** |
| Mm.23049 | Filip1 | Filamin A interacting protein 1 | **-2.27 ± 0.48** |
| Mm.271724 | Dtx3 | Deltex 3 homolog | **-2.38 ± 0.35** |
| Mm.60688 | Ccdc70 | Coiled-coil domain containing 70 | **-2.47 ± 0.32** |
| Mm.441431 | Syn2 | Synapsin II | **-2.55 ± 0.51** |
| Mm.155877 | Ulk3 | Unc-51-like kinase 3 | **-2.55 ± 0.52** |
| Mm.28839 | Eif2b1 | Eukaryotic translation initiation factor 2B, subunit 1 (alpha) | **-2.56 ± 0.48** |
| **Genes uniquely modulated at 96 h pi** | | | |
| Mm.137570 | Bpil3 | Bactericidal/permeability-increasing protein-like 3 | **2.18 ± 0.13** |
| Mm.788 | Ly6e | Lymphocyte antigen 6 complex, locus E | **2.12 ± 0.32** |
| Mm.377095 | Ly6f | Lymphocyte antigen 6 complex, locus F | **2.09 ± 0.32** |
| Mm.271839 | Cmpk2 | Cytidine monophosphate (UMP-CMP) kinase 2, mitochondrial | **2.03 ± 0.33** |
| Mm.76649 | Vcam1 | Vascular cell adhesion molecule 1 | **2.01 ± 0.37** |
| Mm.376121 | Olfr1152 | Olfactory receptor 1152 | **2.00 ± 0.43** |
| Mm.132226 | Ehd4 | EH-domain containing 4 | **2.00 ± 0.13** |
| Mm.103748 | Extl3 | Exostoses (multiple)-like 3 | **1.96 ± 0.13** |
| Mm.390983 | Psmb9 | Proteasome (prosome, macropain) subunit, beta type 9 | **1.88 ± 0.37** |
| Mm.247453 | Abhd10 | Abhydrolase domain containing 10 | **1.87 ± 0.03** |
| Mm.245154 | Tmeff2 | Transmembrane protein with EGF-like and two follistatin-like domains 2 | **1.83 ± 0.15** |
| Mm.293120 | Stat2 | Signal transducer and activator of transcription 2 | **1.76 ± 0.37** |
| Mm.1583 | Ly6c1 | Lymphocyte antigen 6 complex, locus C1 | **1.73 ± 0.32** |
| Mm.234441 | Lmcd1 | LIM and cysteine-rich domains 1 | **1.71 ± 0.07** |
| Mm.290764 | Lrrc61 | Leucine rich repeat containing 61 | **1.67 ± 0.09** |
| Mm.290669 | Ndfip2 | Nedd4 family interacting protein 2 | **1.62 ± 0.15** |
| Mm.223180 | Olfr1195 | Olfactory receptor 1195 | **1.61 ± 0.28** |
| Mm.442861 | H2-D4 | Histocompatibility 2, D region locus 4 | **1.61 ± 0.31** |
| Mm.243758 | Apol9a | Apolipoprotein L 9a | **1.57 ± 0.33** |
| Mm.41385 | Isoc2b | Isochorismatase domain containing 2b | **1.57 ± 0.04** |
| Mm.131422 | Osm | Oncostatin M | **1.47 ± 0.22** |
| Mm.42150 | Rasgrp1 | RAS guanyl releasing protein 1 | **1.43 ± 0.03** |
| Mm.2082 | Apod | Apolipoprotein D | **1.38 ± 0.23** |
| Mm.247736 | Gm4902 | Predicted gene 4902 | **1.36 ± 0.18** |
| Mm.30435 | Tsc2 | Tuberous sclerosis 2 | **1.34 ± 0.29** |
| Mm.241682 | Lrrc4c | Leucine rich repeat containing 4C | **1.33 ± 0.08** |
| Mm.90450 | Myo1a | Myosin IA | **1.30 ± 0.06** |
| Mm.211477 | Phldb2 | Pleckstrin homology-like domain, family B, member 2 | **1.28 ± 0.15** |
| Mm.302715 | EG245575 | PREDICTED: predicted gene, EG245575 | **1.27 ± 0.25** |
| Mm.235538 | Rnase1 | Ribonuclease, Rnase A family, 1 (pancreatic) | **1.24 ± 0.11** |
| Mm.282556 | Npc2 | Niemann Pick type C2 | **1.23 ± 0.18** |
| Mm.357108 | Tmem178 | Transmembrane protein 178 | **1.23 ± 0.15** |
| Mm.358668 | Prpf40b | PRP40 pre-mRNA processing factor 40 homolog B | **1.20 ± 0.09** |
| Mm.148800 | Saa1 | Serum amyloid A 1 | **1.19 ± 0.15** |
| Mm.245851 | Cd200 | CD200 antigen | **1.16 ± 0.16** |
| Mm.302516 | LOC433440 | PREDICTED: similar to fibrous sheath-interacting protein 2 | **1.16 ± 0.12** |
| Mm.275555 | Cnn3 | Calponin 3, acidic | **1.14 ± 0.11** |
| Mm.479273 | Nphp3 | Nephronophthisis 3 | **1.14 ± 0.07** |
| Mm.192991 | Mt1 | Metallothionein 1 | **1.12 ± 0.06** |
| Mm.246513 | Fos | FBJ osteosarcoma oncogene | **1.12 ± 0.13** |
| Mm.170515 | Nfkbia | Nuclear factor of kappa light polypeptide gene enhancer in B-cells inhibitor, alpha | **1.08 ± 0.16** |
| Mm.386931 | Ecm2 | Extracellular matrix protein 2, female organ and adipocyte specific | **1.07 ± 0.07** |
| Mm.1114 | Gla | Galactosidase, alpha | **1.06 ± 0.08** |
| Mm.237935 | Etl4 | Enhancer trap locus 4 | **1.04 ± 0.06** |
| Mm.302287 | Lrrfip2 | Leucine rich repeat (in FLII) interacting protein 2 | **1.03 ± 0.02** |
| Mm.9901 | Nucb2 | Nucleobindin 2 | **1.03 ± 0.17** |
| Mm.181959 | Egr1 | Early growth response 1 | **1.03 ± 0.05** |
| Mm.391427 | 4933416C03Rik | RIKEN cDNA 4933416C03 gene | **1.01 ± 0.14** |
| Mm.332425 | Corin | Corin | **1.00 ± 0.18** |
| Mm.196006 | Mlh1 | Mutl homolog 1 | **-1.00 ± 0.12** |
| Mm.23977 | Ccdc125 | Coiled-coil domain containing 125 | **-1.05 ± 0.11** |
| Mm.136223 | Nrbf2 | Nuclear receptor binding factor 2 | **-1.06 ± 0.23** |
| Mm.234234 | Kdm4a | Lysine (K)-specific demethylase 4A | **-1.10 ± 0.16** |
| Mm.6898 | Naip1 | NLR family, apoptosis inhibitory protein 1 | **-1.15 ± 0.09** |
| Mm.235007 | Ttll1 | Tubulin tyrosine ligase-like 1 | **-1.16 ± 0.06** |
| Mm.39088 | Sox1 | SRY-box containing gene 1 | **-1.16 ± 0.18** |
| Mm.73234 | Cep72 | Centrosomal protein 72 | **-1.19 ± 0.15** |
| Mm.391424 | Ppfia2 | Protein tyrosine phosphatase, receptor type, f polypeptide (PTPRF), interacting protein (liprin), alpha 2 | **-1.19 ± 0.07** |
| Mm.321990 | Lrp6 | Low density lipoprotein receptor-related protein 6 | **-1.20 ± 0.12** |
| Mm.20271 | Fbn2 | Fibrillin 2 | **-1.24 ± 0.11** |
| Mm.72646 | 1700017N19Rik | RIKEN cDNA 1700017N19 gene | **-1.24 ± 0.14** |
| Mm.443526 | Veph1 | Ventricular zone expressed PH domain homolog 1 | **-1.28 ± 0.23** |
| Mm.29628 | B3gnt1 | UDP-glcnac:betagal beta-1,3-N-acetylglucosaminyltransferase 1 | **-1.30 ± 0.18** |
| Mm.130824 | Npsr1 | Neuropeptide S receptor 1 | **-1.34 ± 0.13** |
| Mm.272078 | Als2 | Amyotrophic lateral sclerosis 2 (juvenile) homolog | **-1.39 ± 0.05** |
| Mm.37817 | Cldn23 | Claudin 23 | **-1.46 ± 0.14** |
| Mm.28132 | 5730590G19Rik | RIKEN cDNA 5730590G19 gene | **-1.47 ± 0.14** |
| Mm.458215 | Shf | Src homology 2 domain containing F | **-1.48 ± 0.11** |
| Mm.374850 | Dclre1b | DNA cross-link repair 1B, PSO2 homolog | **-1.50 ± 0.06** |
| Mm.281452 | P2ry1 | Purinergic receptor P2Y, G-protein coupled 1 | **-1.52 ± 0.21** |
| Mm.46561 | Lect1 | Leukocyte cell derived chemotaxin 1 | **-1.53 ± 0.12** |
| Mm.441986 | Fam189a1 | Family with sequence similarity 189, member A1 | **-1.55 ± 0.15** |
| Mm.44763 | 1300018J18Rik | RIKEN cDNA 1300018J18 gene | **-1.61 ± 0.02** |
| Mm.107441 | Zfp26 | Zinc finger protein 26 | **-1.69 ± 0.13** |
| Mm.475593 | Gm4846 | Predicted gene 4846 | **-1.78 ± 0.02** |
| Mm.85280 | Jakmip1 | Janus kinase and microtubule interacting protein 1 | **-1.80 ± 0.10** |
| Mm.32074 | Kcnn1 | Potassium intermediate/small conductance calcium-activated channel, subfamily N, member 1 | **-1.81 ± 0.19** |
| Mm.137 | Ccl6 | Chemokine (C-C motif) ligand 6 | **-1.96 ± 0.12** |
| Mm.33864 | 4930578I06Rik | RIKEN cDNA 4930578I06 gene | **-2.02 ± 0.10** |
| Mm.23788 | Nek8 | NIMA (never in mitosis gene a)-related expressed kinase 8 | **-2.13 ± 0.01** |
| Mm.290822 | Erbb2 | V-erb-b2 erythroblastic leukemia viral oncogene homolog 2, neuro/glioblastoma derived oncogene homolog | **-2.21 ± 0.35** |
| Mm.447819 | Klk1b1 | Kallikrein 1-related peptidase b1 | **-2.36 ± 0.07** |
| Mm.234204 | Pak2 | P21 protein (Cdc42/Rac)-activated kinase 2 | **-2.49 ± 0.44** |
| Mm.147213 | B230218P12Rik | PREDICTED: RIKEN cDNA B230218P12 gene, transcript variant 2 | **-2.80 ± 0.49** |
| Mm.472701 | Meis3 | Meis homeobox | **-2.80 ± 0.17** |
| Mm.12834 | Lfng | LFNG O-fucosylpeptide 3-beta-N-acetylglucosaminyltransferase | **-3.18 ± 0.10** |
| Mm.207203 | Stxbp4 | Syntaxin binding protein 4 | **-3.32 ± 0.30** |
| Mm.288567 | Hbb-b1 | Hemoglobin, beta adult major chain | **-4.16 ± 0.20** |
