# Supplementary material for: Differential host gene responses from infection with neurovirulent and partially-neurovirulent strains of Venezuelan equine encephalitis virus
Source: BMC Infect Dis. 2017 Apr 26;17:309. doi: 10.1186/s12879-017-2355-3 (PMC5405508; doi:10.1186/s12879-017-2355-3)
Supplement: Supplementary file 6 — Significantly modulated genes in the spleen that were unique to V3034 infection. Genes that were modulated only with V3034 infection in the spleen were identified. The list summarizes the commonly modulated genes for each time point studied. Values are expressed as average values of (log2) fold expression for each gene over uninfected controls ± standard error mean (SEM). * P ≤ 0.05. (DOCX 50 kb) [file 12879_2017_2355_MOESM6_ESM.docx]

**Additional file 6: Table S5: Significantly modulated genes in spleen that were unique to V3034 infection.**

| **UniGene** | **Gene** | **Description** | **Log_2_ Exp ± SEM** |
| --- | --- | --- | --- |
| **Genes uniquely modulated at 24 h pi** | | | |
| Mm.485161 | Slc15a3 | Solute carrier family 15, member 3 | **1.85 ± 0.37** |
| Mm.190461 | Amica1 | Adhesion molecule, interacts with CXADR antigen 1 | **1.71 ± 0.31** |
| Mm.466882 | H2-K1 | Histocompatibility 2, K1, K region | **1.63 ± 0.08** |
| Mm.299312 | Nhp2l1 | NHP2 non-histone chromosome protein 2-like 1 | **1.58 ± 0.07** |
| Mm.21482 | Clns1a | Chloride channel, nucleotide-sensitive, 1A | **1.55 ± 0.24** |
| Mm.391959 | 1500012F01Rik | RIKEN cDNA 1500012F01 gene | **1.54 ± 0.24** |
| Mm.341848 | Fcf1 | FCF1 small subunit (SSU) processome component homolog | **1.51 ± 0.21** |
| Mm.436873 | Gm10229 | Predicted gene 10229 | **1.50 ± 0.13** |
| Mm.250004 | Gars | Glycyl-tRNA synthetase | **1.46 ± 0.28** |
| Mm.27545 | Prmt1 | Protein arginine N-methyltransferase 1 | **1.39 ± 0.17** |
| Mm.290906 | Sp100 | Nuclear antigen Sp100 | **1.34 ± 0.26** |
| Mm.275349 | Gemin5 | Gem (nuclear organelle) associated protein 5 | **1.23 ± 0.01** |
| Mm.163 | B2m | Beta-2 microglobulin | **1.16 ± 0.19** |
| Mm.439702 | Nme1 | Non-metastatic cells 1, protein (NM23A) expressed in | **1.07 ± 0.23** |
| Mm.28536 | Dpy30 | Dpy-30 homolog | **1.06 ± 0.20** |
| Mm.140 | Ppp1r14b | Protein phosphatase 1, regulatory (inhibitor) subunit 14B | **1.01 ± 0.19** |
| Mm.19379 | Lst1 | Leukocyte specific transcript 1 | **1.01 ± 0.21** |
| Mm.160080 | 5330426L24Rik | RIKEN cDNA 5330426L24 gene | **1.01 ± 0.23** |
| Mm.235981 | 2010001E11Rik | RIKEN cDNA 2010001E11 gene | **-1.02 ± 0.12** |
| Mm.33360 | Mylk | Myosin, light polypeptide kinase | **-1.02 ± 0.18** |
| Mm.193212 | Hyi | Hydroxypyruvate isomerase homolog | **-1.02 ± 0.18** |
| Mm.222632 | Il27 | Interleukin 27 | **-1.03 ± 0.10** |
| Mm.379006 | Rps29 | Ribosomal protein S29 | **-1.04 ± 0.21** |
| Mm.302938 | Ddx3y | DEAD (Asp-Glu-Ala-Asp) box polypeptide 3, Y-linked | **-1.05 ± 0.19** |
| Mm.180013 | Aes | Amino-terminal enhancer of split | **-1.07 ± 0.14** |
| Mm.271711 | Tagln2 | Transgelin 2 | **-1.10 ± 0.11** |
| Mm.247218 | Mpi | Mannose phosphate isomerase | **-1.14 ± 0.18** |
| Mm.202715 | Sun2 | Sad1 and UNC84 domain containing 2 | **-1.16 ± 0.17** |
| Mm.368330 | Il13ra2 | Interleukin 13 receptor, alpha 2 | **-1.22 ± 0.24** |
| Mm.271230 | Srrm3 | Serine/arginine repetitive matrix 3 | **-1.26 ± 0.20** |
| Mm.250425 | Fam192a | Family with sequence similarity 192, member A | **-1.27 ± 0.14** |
| Mm.27366 | Gramd1a | GRAM domain containing 1A | **-1.57 ± 0.32** |
| Mm.205190 | Mll5 | Myeloid/lymphoid or mixed-lineage leukemia 5 | **-1.61 ± 0.08** |
| Mm.373589 | Dcbld2 | Discoidin, CUB and LCCL domain containing 2 | **-1.81 ± 0.08** |
| Mm.29945 | Edf1 | Endothelial differentiation-related factor 1 | **-1.86 ± 0.27** |
| Mm.23596 | Cenpm | Centromere protein M | **-3.79 ± 0.87** |
| **Genes uniquely modulated at 48 h pi** | | | |
| Mm.271809 | Daxx | Fas death domain-associated protein | **1.80 ± 0.26** |
| Mm.386456 | A530032D15Rik | RIKEN cDNA A530032D15Rik gene | **1.79 ± 0.02** |
| Mm.252255 | Psma2 | Proteasome (prosome, macropain) subunit, alpha type 2 | **1.78 ± 0.02** |
| Mm.34428 | Pias4 | Protein inhibitor of activated STAT 4 | **1.76 ± 0.28** |
| Mm.261140 | Iigp1 | Interferon inducible GTPase 1 | **1.74 ± 0.06** |
| Mm.194450 | Smchd1 | SMC hinge domain containing 1 | **1.72 ± 0.31** |
| Mm.280559 | Tmem106a | Transmembrane protein 106A | **1.70 ± 0.26** |
| Mm.282556 | Npc2 | Niemann Pick type C2 | **1.69 ± 0.03** |
| Mm.390983 | Psmb9 | Proteasome (prosome, macropain) subunit, beta type 9 | **1.69 ± 0.33** |
| Mm.65389 | Tsga14 | Testis specific gene A14 | **1.68 ± 0.26** |
| Mm.265929 | Vti1b | Vesicle transport through interaction with t-snares 1B homolog | **1.65 ± 0.14** |
| Mm.105218 | Irf1 | Interferon regulatory factor 1 | **1.63 ± 0.29** |
| Mm.33957 | Ms4a4b | Membrane-spanning 4-domains, subfamily A, member 4B | **1.62 ± 0.17** |
| Mm.2699 | AF251705 | CDNA sequence AF251705 | **1.58 ± 0.32** |
| Mm.300317 | Csgalnact2 | Chondroitin sulfate N-acetylgalactosaminyltransferase 2 | **1.58 ± 0.05** |
| Mm.195803 | Nnt | Nicotinamide nucleotide transhydrogenase | **1.58 ± 0.21** |
| Mm.291131 | Xaf1 | XIAP associated factor 1 | **1.55 ± 0.17** |
| Mm.332425 | Corin | Corin | **1.51 ± 0.04** |
| Mm.270511 | Tcerg1 | Transcription elongation regulator 1 (CA150) | **1.49 ± 0.16** |
| Mm.34479 | Fam26f | Family with sequence similarity 26, member F | **1.49 ± 0.16** |
| Mm.475713 | Ano8 | Anoctamin 8 | **1.48 ± 0.20** |
| Mm.25684 | Clec4a3 | C-type lectin domain family 4, member a3 | **1.44 ± 0.18** |
| Mm.190461 | Amica1 | Adhesion molecule, interacts with CXADR antigen 1 | **1.43 ± 0.25** |
| Mm.270065 | Gas5 | Growth arrest specific 5 | **1.43 ± 0.12** |
| Mm.208883 | Psma5 | Proteasome (prosome, macropain) subunit, alpha type 5 | **1.41 ± 0.14** |
| Mm.32012 | Ncapg | Non-SMC condensin I complex, subunit G | **1.40 ± 0.16** |
| Mm.204634 | Rad51ap1 | RAD51 associated protein 1 | **1.38 ± 0.11** |
| Mm.141936 | Igfbp2 | Insulin-like growth factor binding protein 2 | **1.37 ± 0.01** |
| Mm.290669 | Ndfip2 | Nedd4 family interacting protein 2 | **1.37 ± 0.28** |
| Mm.24521 | Zfand2a | Zinc finger, AN1-type domain 2A | **1.34 ± 0.25** |
| Mm.352311 | Dtnbp1 | Dystrobrevin binding protein 1 | **1.34 ± 0.28** |
| Mm.389251 | Psmb7 | Proteasome (prosome, macropain) subunit, beta type 7 | **1.34 ± 0.25** |
| Mm.29590 | Idh3b | Isocitrate dehydrogenase 3 (NAD+) beta | **1.34 ± 0.29** |
| Mm.653 | Cfb | Complement factor B | **1.33 ± 0.08** |
| Mm.436639 | Ms4a6b | Membrane-spanning 4-domains, subfamily A, member 6B | **1.32 ± 0.16** |
| Mm.26680 | Snap47 | Synaptosomal-associated protein, 47 | **1.31 ± 0.24** |
| Mm.4735 | Was | Wiskott-Aldrich syndrome homolog | **1.31 ± 0.29** |
| Mm.290912 | BC013712 | CDNA sequence BC013712 | **1.30 ± 0.26** |
| Mm.290320 | Ccl2 | Chemokine (C-C motif) ligand 2 | **1.29 ± 0.14** |
| Mm.16716 | Lamp1 | Lysosomal-associated membrane protein 1 | **1.29 ± 0.09** |
| Mm.253627 | Snx31 | Sorting nexin 31 | **1.28 ± 0.04** |
| Mm.8217 | Klrk1 | Killer cell lectin-like receptor subfamily K, member 1 | **1.27 ± 0.11** |
| Mm.57223 | Hells | Helicase, lymphoid specific | **1.26 ± 0.18** |
| Mm.273379 | Snx5 | Sorting nexin 5 | **1.26 ± 0.07** |
| Mm.355701 | 1810035L17Rik | RIKEN cDNA 1810035L17 gene | **1.26 ± 0.19** |
| Mm.163 | B2m | Beta-2 microglobulin | **1.26 ± 0.12** |
| Mm.41737 | Gng2 | Guanine nucleotide binding protein (G protein), gamma 2 | **1.25 ± 0.13** |
| Mm.41339 | Myo5c | Myosin VC | **1.25 ± 0.08** |
| Mm.11889 | Nlrp9a | NLR family, pyrin domain containing 9A | **1.25 ± 0.02** |
| Mm.354760 | Pam16 | Presequence translocase-asssociated motor 16 homolog | **1.24 ± 0.09** |
| Mm.74610 | Fam46a | Family with sequence similarity 46, member A | **1.24 ± 0.27** |
| Mm.294753 | Litaf | LPS-induced TN factor | **1.21 ± 0.06** |
| Mm.85280 | Jakmip1 | Janus kinase and microtubule interacting protein 1 | **1.21 ± 0.17** |
| Mm.831 | Ssr4 | Signal sequence receptor, delta | **1.20 ± 0.12** |
| Mm.391106 | Pde6a | Phosphodiesterase 6A, cgmp-specific, rod, alpha | **1.20 ± 0.13** |
| Mm.830 | Psme1 | Proteasome (prosome, macropain) 28 subunit, alpha | **1.19 ± 0.07** |
| Mm.21353 | Dnajc11 | Dnaj (Hsp40) homolog, subfamily C, member 11 | **1.19 ± 0.03** |
| Mm.330160 | Hspa5 | Heat shock protein 5 | **1.18 ± 0.14** |
| Mm.272569 | Serpinb9 | Serine (or cysteine) peptidase inhibitor, clade B, member 9 | **1.18 ± 0.05** |
| Mm.388573 | Ttll3 | Tubulin tyrosine ligase-like family, member 3 | **1.17 ± 0.18** |
| Mm.136648 | Tpd52l2 | Tumor protein D52-like 2 | **1.17 ± 0.17** |
| Mm.78341 | 4933402N03Rik | RIKEN cDNA 4933402N03 gene | **1.16 ± 0.26** |
| Mm.336045 | Utp14a | UTP14, U3 small nucleolar ribonucleoprotein, homolog A | **1.15 ± 0.18** |
| Mm.154378 | Ncl | Nucleolin (Ncl), mrna. | **1.15 ± 0.22** |
| Mm.288697 | Slc25a27 | Solute carrier family 25, member 27 | **1.15 ± 0.13** |
| Mm.74465 | Atp8b5 | ATPase, class I, type 8B, member 5 | **1.15 ± 0.17** |
| Mm.380129 | G3bp1 | Ras-GTPase-activating protein SH3-domain binding protein 1 | **1.15 ± 0.11** |
| Mm.289992 | Eif3e | Eukaryotic translation initiation factor 3, subunit E | **1.13 ± 0.13** |
| Mm.425502 | Trim12c | Tripartite motif-containing 12C | **1.13 ± 0.17** |
| Mm.333406 | Ccnd2 | Cyclin D2 | **1.13 ± 0.09** |
| Mm.259293 | 2700094K13Rik | RIKEN cDNA 2700094K13 gene | **1.12 ± 0.05** |
| Mm.239514 | Traf1 | TNF receptor-associated factor 1 | **1.12 ± 0.08** |
| Mm.330948 | Egfem1 | EGF-like and EMI domain containing 1 | **1.12 ± 0.24** |
| Mm.2180 | Hsp90ab1 | Heat shock protein 90 alpha (cytosolic), class B member 1 | **1.12 ± 0.20** |
| Mm.248445 | Trim25 | Tripartite motif-containing 25 | **1.12 ± 0.14** |
| Mm.248046 | Dnajc15 | Dnaj (Hsp40) homolog, subfamily C, member 15 | **1.12 ± 0.15** |
| Mm.30270 | Psma4 | Proteasome (prosome, macropain) subunit, alpha type 4 | **1.11 ± 0.16** |
| Mm.477808 | H2-Bl | Histocompatibility 2, blastocyst | **1.11 ± 0.25** |
| Mm.188516 | Smtn | Smoothelin | **1.11 ± 0.13** |
| Mm.2299 | Cd244 | CD244 natural killer cell receptor 2B4 | **1.10 ± 0.17** |
| Mm.248163 | Mrpl52 | Mitochondrial ribosomal protein L52 | **1.10 ± 0.08** |
| Mm.300397 | Whrn | Whirlin | **1.10 ± 0.03** |
| Mm.296902 | Tapbpl | TAP binding protein-like | **1.10 ± 0.11** |
| Mm.152627 | Cox7a2 | Cytochrome c oxidase, subunit VIIa 2 | **1.10 ± 0.20** |
| Mm.21938 | Rps20 | Ribosomal protein S20 | **1.09 ± 0.17** |
| Mm.2639 | Ly86 | Lymphocyte antigen 86 | **1.09 ± 0.02** |
| Mm.301148 | Taf9 | TAF9 RNA polymerase II, TATA box binding protein (TBP)-associated factor | **1.09 ± 0.11** |
| Mm.26212 | Med21 | Mediator complex subunit 21 | **1.08 ± 0.19** |
| Mm.486022 | 9330175E14Rik | RIKEN cDNA 9330175E14 gene | **1.08 ± 0.21** |
| Mm.144143 | Camsap1l1 | Calmodulin regulated spectrin-associated protein 1-like 1 | **1.07 ± 0.08** |
| Mm.283045 | Med28 | Mediator of RNA polymerase II transcription, subunit 28 homolog | **1.07 ± 0.07** |
| Mm.371609 | Pdcd5 | Programmed cell death 5 | **1.07 ± 0.07** |
| Mm.38010 | Atic | 5-aminoimidazole-4-carboxamide ribonucleotide formyltransferase/IMP cyclohydrolase | **1.07 ± 0.11** |
| Mm.198414 | Stk39 | Serine/threonine kinase 39, STE20/SPS1 homolog | **1.06 ± 0.01** |
| Mm.267692 | Glrx3 | Glutaredoxin 3 | **1.06 ± 0.16** |
| Mm.87820 | Sprr2d | Small proline-rich protein 2D | **1.05 ± 0.15** |
| Mm.209491 | Fnbp1l | Formin binding protein 1-like | **1.05 ± 0.04** |
| Mm.67938 | Cstf2 | Cleavage stimulation factor, 3' pre-RNA subunit 2 | **1.05 ± 0.24** |
| Mm.30043 | Spcs2 | Signal peptidase complex subunit 2 homolog | **1.04 ± 0.08** |
| Mm.435590 | Nfyc | Nuclear transcription factor-Y gamma | **1.04 ± 0.09** |
| Mm.273403 | Cox5a | Cytochrome c oxidase, subunit Va | **1.04 ± 0.05** |
| Mm.3333 | Apobec1 | Apolipoprotein B mRNA editing enzyme, catalytic polypeptide 1 | **1.03 ± 0.22** |
| Mm.19379 | Lst1 | Leukocyte specific transcript 1 | **1.03 ± 0.19** |
| Mm.258155 | Dock5 | Dedicator of cytokinesis 5 | **1.03 ± 0.07** |
| Mm.334789 | Nip7 | Nuclear import 7 homolog | **1.03 ± 0.16** |
| Mm.27210 | G6pdx | Glucose-6-phosphate dehydrogenase X-linked | **1.02 ± 0.22** |
| Mm.28536 | Dpy30 | Dpy-30 homolog | **1.02 ± 0.15** |
| Mm.28528 | Rwdd1 | RWD domain containing 1 | **1.02 ± 0.13** |
| Mm.475640 | Cpsf3l | Cleavage and polyadenylation specific factor 3-like | **1.01 ± 0.11** |
| Mm.259045 | Actr2 | ARP2 actin-related protein 2 homolog | **1.01 ± 0.21** |
| Mm.18472 | Psmc6 | Proteasome (prosome, macropain) 26S subunit, ATPase, 6 | **1.01 ± 0.14** |
| Mm.1410 | Il18 | Interleukin 18 | **1.00 ± 0.18** |
| Mm.223310 | Olfr1213 | Olfactory receptor 1213 | **1.00 ± 0.17** |
| Mm.213114 | Zkscan1 | Zinc finger with KRAB and SCAN domains 1 | **1.00 ± 0.16** |
| Mm.156365 | Tmem215 | Transmembrane protein 215 | **1.00 ± 0.14** |
| Mm.10116 | Cxcl13 | Chemokine (C-X-C motif) ligand 13 | **1.00 ± 0.08** |
| Mm.136347 | 4930529M08Rik | RIKEN cDNA 4930529M08 gene | **-1.00 ± 0.11** |
| Mm.320384 | Serp2 | Stress-associated endoplasmic reticulum protein family member 2 | **-1.02 ± 0.20** |
| Mm.7884 | Isca1 | Iron-sulfur cluster assembly 1 homolog | **-1.02 ± 0.13** |
| Mm.316249 | Mctp1 | Multiple C2 domains, transmembrane 1 | **-1.03 ± 0.22** |
| Mm.56097 | Fam40b | Family with sequence similarity 40, member B | **-1.04 ± 0.16** |
| Mm.41420 | Tbc1d10b | TBC1 domain family, member 10b | **-1.04 ± 0.17** |
| Mm.269699 | X99384 | CDNA sequence X99384 | **-1.05 ± 0.07** |
| Mm.258923 | Nucb1 | Nucleobindin 1 | **-1.06 ± 0.19** |
| Mm.121485 | Sfxn5 | Sideroflexin 5 | **-1.06 ± 0.23** |
| Mm.28209 | Perp | PERP, TP53 apoptosis effector | **-1.08 ± 0.07** |
| Mm.22270 | Tspan8 | Tetraspanin 8 | **-1.08 ± 0.12** |
| Mm.212921 | 4930432E11Rik | PREDICTED: RIKEN cDNA 4930432E11 gene | **-1.09 ± 0.24** |
| Mm.18802 | 1300017J02Rik | RIKEN cDNA 1300017J02 gene | **-1.12 ± 0.18** |
| Mm.296240 | 2310037I24Rik | RIKEN cDNA 2310037I24 gene | **-1.12 ± 0.17** |
| Mm.282122 | Ubfd1 | Ubiquitin family domain containing 1 | **-1.13 ± 0.17** |
| Mm.386829 | Tsga10ip | PREDICTED: testis specific 10 interacting protein | **-1.13 ± 0.06** |
| Mm.377875 | Hist1h4f | Histone cluster 1, H4f | **-1.13 ± 0.05** |
| Mm.311110 | Park2 | Parkinson disease (autosomal recessive, juvenile) 2, parkin | **-1.13 ± 0.24** |
| Mm.214953 | Mettl22 | Methyltransferase like 22 | **-1.14 ± 0.10** |
| Mm.57734 | Lims1 | LIM and senescent cell antigen-like domains 1 | **-1.16 ± 0.08** |
| Mm.12897 | Gp9 | Glycoprotein 9 (platelet) | **-1.16 ± 0.17** |
| Mm.302724 | Alas2 | Aminolevulinic acid synthase 2, erythroid | **-1.19 ± 0.19** |
| Mm.334306 | Olfr523 | Olfactory receptor 523 | **-1.20 ± 0.17** |
| Mm.20805 | Phf23 | PHD finger protein 23 | **-1.24 ± 0.13** |
| Mm.28888 | Irx2 | Iroquois related homeobox 2 | **-1.25 ± 0.27** |
| Mm.29900 | Tsfm | Ts translation elongation factor, mitochondrial | **-1.26 ± 0.10** |
| Mm.25814 | 2310046O06Rik | RIKEN cDNA 2310046O06 gene | **-1.27 ± 0.18** |
| Mm.63490 | Ces1e | Carboxylesterase 1E | **-1.27 ± 0.26** |
| Mm.212446 | Chst2 | Carbohydrate sulfotransferase 2 | **-1.27 ± 0.28** |
| Mm.158337 | Ctsll3 | Cathepsin L-like 3 | **-1.28 ± 0.14** |
| Mm.259767 | Mypn | Myopalladin | **-1.29 ± 0.24** |
| Mm.485856 | Zfp87 | Zinc finger protein 87 | **-1.30 ± 0.12** |
| Mm.256422 | Sf1 | Splicing factor 1 | **-1.35 ± 0.09** |
| Mm.439737 | Cd74 | CD74 antigen | **-1.37 ± 0.12** |
| Mm.277373 | Camk1 | Calcium/calmodulin-dependent protein kinase I | **-1.38 ± 0.10** |
| Mm.202665 | Rnase4 | Ribonuclease, rnase A family 4 | **-1.39 ± 0.12** |
| Mm.217385 | Ccdc111 | Coiled-coil domain containing 111 | **-1.42 ± 0.17** |
| Mm.26690 | Dolk | Dolichol kinase | **-1.43 ± 0.24** |
| Mm.277148 | Slc5a12 | Solute carrier family 5 (sodium/glucose cotransporter), member 12 | **-1.44 ± 0.18** |
| Mm.170023 | Rnf181 | Ring finger protein 181 | **-1.47 ± 0.13** |
| Mm.288741 | Scap | SREBF chaperone | **-1.47 ± 0.09** |
| Mm.332225 | Speer4f | Spermatogenesis associated glutamate (E)-rich protein 4f | **-1.48 ± 0.17** |
| Mm.1640 | Cdr2 | Cerebellar degeneration-related 2 | **-1.53 ± 0.30** |
| Mm.121859 | Klrg2 | Killer cell lectin-like receptor subfamily G, member 2 | **-1.53 ± 0.33** |
| Mm.472596 | Olfr1297 | Olfactory receptor 1297 | **-1.54 ± 0.11** |
| Mm.276466 | Hsd17b2 | Hydroxysteroid (17-beta) dehydrogenase 2 | **-1.61 ± 0.25** |
| Mm.4475 | Btk | Bruton agammaglobulinemia tyrosine kinase | **-1.63 ± 0.11** |
| Mm.17484 | Snca | Synuclein, alpha | **-1.67 ± 0.34** |
| Mm.251908 | Akr1e1 | Aldo-keto reductase family 1, member E1 | **-1.67 ± 0.18** |
| Mm.29914 | Cbfa2t2 | Core-binding factor, runt domain, alpha subunit 2, translocated to, 2 | **-1.73 ± 0.13** |
| Mm.181278 | Vps24 | Vacuolar protein sorting 24 | **-1.76 ± 0.25** |
| Mm.273303 | Spdyb | Speedy homolog B | **-1.77 ± 0.05** |
| Mm.3943 | Thpo | Thrombopoietin | **-1.79 ± 0.06** |
| Mm.5903 | Gpaa1 | GPI anchor attachment protein 1 | **-1.83 ± 0.26** |
| Mm.389971 | Olfr669 | Olfactory receptor 669 | **-1.83 ± 0.06** |
| Mm.7838 | Ccndbp1 | Cyclin D-type binding-protein 1 | **-1.89 ± 0.33** |
| Mm.240252 | Trim14 | Tripartite motif-containing 14 | **-2.01 ± 0.14** |
| Mm.277465 | Hspa12b | Heat shock protein 12B | **-2.01 ± 0.14** |
| Mm.470126 | Hbb-b2 | Hemoglobin, beta adult minor chain | **-2.08 ± 0.21** |
| Mm.196006 | Mlh1 | Mutl homolog 1 | **-2.08 ± 0.08** |
| Mm.440055 | Krt80 | Keratin 80 | **-2.10 ± 0.24** |
| Mm.44257 | Spats1 | Spermatogenesis associated, serine-rich 1 | **-2.10 ± 0.15** |
| Mm.29945 | Edf1 | Endothelial differentiation-related factor 1 | **-2.15 ± 0.23** |
| Mm.159541 | 1700020C07Rik | RIKEN cDNA 1700020C07 gene | **-2.29 ± 0.20** |
| Mm.423664 | Gm2058 | Predicted gene 2058 | **-2.31 ± 0.10** |
| Mm.46431 | 2310002J15Rik | RIKEN cDNA 2310002J15 gene | **-2.42 ± 0.19** |
| Mm.25237 | Slc5a1 | Solute carrier family 5 (sodium/glucose cotransporter), member 1 | **-2.57 ± 0.17** |
| Mm.447819 | Klk1b1 | Kallikrein 1-related peptidase b1 | **-2.59 ± 0.37** |
| Mm.377902 | Tas2r102 | Taste receptor, type 2, member 102 | **-2.91 ± 0.18** |
| Mm.260690 | Lrrc47 | Leucine rich repeat containing 47 | **-3.13 ± 0.21** |
| Mm.28438 | Txndc9 | Thioredoxin domain containing 9 | **-4.13 ± 0.20** |
| Mm.207203 | Stxbp4 | Syntaxin binding protein 4 | **-4.69 ± 0.63** |
| **Genes uniquely modulated at 72 h pi** | | | |
| Mm.250564 | Hist1h2ao | Histone cluster 1, h2ao | **3.02 ± 0.38** |
| Mm.33902 | Igtp | Interferon gamma induced GTPase | **2.78 ± 0.15** |
| Mm.377095 | Ly6f | Lymphocyte antigen 6 complex, locus F | **2.68 ± 0.18** |
| Mm.289747 | Cdc20 | Cell division cycle 20 homolog | **2.64 ± 0.44** |
| Mm.284114 | 1110034G24Rik | RIKEN cDNA 1110034G24 gene | **2.62 ± 0.39** |
| Mm.377877 | Hist1h2an | Histone cluster 1, h2an | **2.35 ± 0.20** |
| Mm.257590 | Ncapd2 | Non-SMC condensin I complex, subunit D2 | **2.29 ± 0.41** |
| Mm.2171 | Atpif1 | ATPase inhibitory factor 1 | **2.21 ± 0.34** |
| Mm.22673 | Fcer1g | Fc receptor, IgE, high affinity I, gamma polypeptide | **2.13 ± 0.44** |
| Mm.422826 | Hist1h2ap | Histone cluster 1, h2ap | **2.10 ± 0.30** |
| Mm.390553 | Hist1h2ad | Histone cluster 1, h2ad | **2.10 ± 0.11** |
| Mm.38055 | Esd | Esterase D/formylglutathione hydrolase | **2.09 ± 0.26** |
| Mm.260325 | Bst2 | Bone marrow stromal cell antigen 2 | **2.08 ± 0.09** |
| Mm.688 | Bag1 | BCL2-associated athanogene 1 | **2.07 ± 0.26** |
| Mm.19379 | Lst1 | Leukocyte specific transcript 1 | **2.02 ± 0.22** |
| Mm.30012 | Hdlbp | High density lipoprotein (HDL) binding protein | **1.94 ± 0.17** |
| Mm.485399 | Atp6v1g1 | ATPase, H+ transporting, lysosomal V1 subunit G1 | **1.93 ± 0.35** |
| Mm.20273 | Urm1 | Ubiquitin related modifier 1 homolog | **1.86 ± 0.07** |
| Mm.133851 | Mrpl12 | Mitochondrial ribosomal protein L12 | **1.86 ± 0.33** |
| Mm.21874 | Psmb3 | Proteasome (prosome, macropain) subunit, beta type 3 | **1.82 ± 0.31** |
| Mm.102540 | Chtf18 | CTF18, chromosome transmission fidelity factor 18 homolog | **1.81 ± 0.24** |
| Mm.273403 | Cox5a | Cytochrome c oxidase, subunit Va | **1.81 ± 0.16** |
| Mm.5220 | Hist2h2aa2 | Histone cluster 2, h2aa2 | **1.80 ± 0.10** |
| Mm.347009 | Prdx2 | Peroxiredoxin 2 | **1.80 ± 0.24** |
| Mm.330390 | Olfr140 | Olfactory receptor 140 | **1.79 ± 0.20** |
| Mm.227704 | Vdac3 | Voltage-dependent anion channel 3 | **1.77 ± 0.25** |
| Mm.248615 | Lgals3 | Lectin, galactose binding, soluble 3 | **1.77 ± 0.13** |
| Mm.250418 | Ogfr | Opioid growth factor receptor | **1.76 ± 0.32** |
| Mm.250004 | Gars | Glycyl-tRNA synthetase | **1.75 ± 0.31** |
| Mm.43831 | Lgals1 | Lectin, galactose binding, soluble 1 | **1.75 ± 0.38** |
| Mm.28349 | Ndufv3 | NADH dehydrogenase (ubiquinone) flavoprotein 3 | **1.73 ± 0.10** |
| Mm.290563 | Cenpa | Centromere protein A | **1.73 ± 0.24** |
| Mm.380129 | G3bp1 | Ras-GTPase-activating protein SH3-domain binding protein 1 | **1.72 ± 0.15** |
| Mm.132208 | Ipo11 | Importin 11 | **1.72 ± 0.32** |
| Mm.266811 | Tada3 | Transcriptional adaptor 3 | **1.71 ± 0.10** |
| Mm.2570 | C1qb | Complement component 1, q subcomponent, beta polypeptide | **1.68 ± 0.11** |
| Mm.29182 | Taldo1 | Transaldolase 1 | **1.67 ± 0.32** |
| Mm.377878 | Hist1h2af | Histone cluster 1, h2af | **1.67 ± 0.31** |
| Mm.260193 | Arrb1 | Arrestin, beta 1 | **1.66 ± 0.36** |
| Mm.110220 | Ddit3 | DNA-damage inducible transcript 3 | **1.66 ± 0.27** |
| Mm.288960 | Mrpl47 | Mitochondrial ribosomal protein L47 | **1.66 ± 0.25** |
| Mm.485161 | Slc15a3 | Solute carrier family 15, member 3 | **1.65 ± 0.35** |
| Mm.260539 | Psmd11 | Proteasome (prosome, macropain) 26S subunit, non-ATPase, 11 | **1.65 ± 0.18** |
| Mm.237594 | Ufd1l | Ubiquitin fusion degradation 1 like | **1.64 ± 0.30** |
| Mm.227260 | Tubb2c | Tubulin, beta 2C | **1.62 ± 0.33** |
| Mm.24632 | Gtf2f1 | General transcription factor IIF, polypeptide 1 | **1.59 ± 0.23** |
| Mm.27499 | Tmem147 | Transmembrane protein 147 | **1.59 ± 0.29** |
| Mm.285366 | Slc7a14 | Solute carrier family 7 (cationic amino acid transporter, y+ system), member 14 | **1.57 ± 0.30** |
| Mm.341848 | Fcf1 | FCF1 small subunit (SSU) processome component homolog | **1.56 ± 0.26** |
| Mm.69 | Nudc | Nuclear distribution gene C homolog | **1.56 ± 0.25** |
| Mm.473730 | Mrpl48 | Mitochondrial ribosomal protein L48 | **1.54 ± 0.18** |
| Mm.290876 | Sod2 | Superoxide dismutase 2, mitochondrial | **1.54 ± 0.16** |
| Mm.27114 | Ypel3 | Yippee-like 3 | **1.53 ± 0.06** |
| Mm.289329 | Metap2 | Methionine aminopeptidase 2 | **1.52 ± 0.28** |
| Mm.157105 | Psmc1 | Protease (prosome, macropain) 26S subunit, ATPase 1 | **1.52 ± 0.14** |
| Mm.275332 | Atg7 | Autophagy-related 7 | **1.52 ± 0.10** |
| Mm.25125 | Prpsap1 | Phosphoribosyl pyrophosphate synthetase-associated protein 1 | **1.51 ± 0.28** |
| Mm.258530 | Sae1 | SUMO1 activating enzyme subunit 1 | **1.49 ± 0.18** |
| Mm.440715 | Ms4a4c | Membrane-spanning 4-domains, subfamily A, member 4C | **1.48 ± 0.28** |
| Mm.341186 | Hsp90aa1 | Heat shock protein 90, alpha (cytosolic), class A member 1 | **1.48 ± 0.15** |
| Mm.30049 | C1qbp | Complement component 1, q subcomponent binding protein | **1.46 ± 0.28** |
| Mm.927 | Bub3 | Budding uninhibited by benzimidazoles 3 homolog | **1.46 ± 0.30** |
| Mm.439702 | Nme1 | Non-metastatic cells 1, protein (NM23A) expressed in | **1.46 ± 0.16** |
| Mm.243234 | Psmd2 | Proteasome (prosome, macropain) 26S subunit, non-ATPase, 2 | **1.46 ± 0.19** |
| Mm.35389 | Cycs | Cytochrome c, somatic | **1.46 ± 0.14** |
| Mm.277125 | Uba7 | Ubiquitin-like modifier activating enzyme 7 | **1.45 ± 0.22** |
| Mm.4742 | Pa2g4 | Proliferation-associated 2G4 | **1.41 ± 0.20** |
| Mm.279782 | Prdx5 | Peroxiredoxin 5 | **1.41 ± 0.15** |
| Mm.41974 | Nkx2-5 | NK2 transcription factor related, locus 5 | **1.40 ± 0.16** |
| Mm.248478 | Samhd1 | SAM domain and HD domain, 1 | **1.40 ± 0.25** |
| Mm.380115 | Polr2l | Polymerase (RNA) II (DNA directed) polypeptide L | **1.39 ± 0.08** |
| Mm.269130 | Rxfp4 | Relaxin family peptide receptor 4 | **1.39 ± 0.09** |
| Mm.400 | Cox6b1 | Cytochrome c oxidase, subunit VIb polypeptide 1 | **1.39 ± 0.20** |
| Mm.18472 | Psmc6 | Proteasome (prosome, macropain) 26S subunit, ATPase, 6 | **1.38 ± 0.23** |
| Mm.289929 | Smu1 | Smu-1 suppressor of mec-8 and unc-52 homolog | **1.38 ± 0.11** |
| Mm.17851 | Ndufa3 | NADH dehydrogenase (ubiquinone) 1 alpha subcomplex, 3 | **1.37 ± 0.17** |
| Mm.277916 | Ptpn1 | Protein tyrosine phosphatase, non-receptor type 1 | **1.37 ± 0.21** |
| Mm.140 | Ppp1r14b | Protein phosphatase 1, regulatory (inhibitor) subunit 14B | **1.37 ± 0.28** |
| Mm.30008 | Mrpl42 | Mitochondrial ribosomal protein L42 | **1.36 ± 0.10** |
| Mm.29135 | Snrpd2 | Small nuclear ribonucleoprotein D2 | **1.35 ± 0.07** |
| Mm.284248 | Ccl5 | Chemokine (C-C motif) ligand 5 | **1.35 ± 0.19** |
| Mm.2326 | Mif | Macrophage migration inhibitory factor | **1.34 ± 0.03** |
| Mm.466670 | Rnf126 | Ring finger protein 126 | **1.33 ± 0.26** |
| Mm.14825 | Idh3g | Isocitrate dehydrogenase 3 (NAD+), gamma | **1.31 ± 0.10** |
| Mm.9953 | Cmc1 | COX assembly mitochondrial protein homolog | **1.31 ± 0.15** |
| Mm.177991 | Krtcap2 | Keratinocyte associated protein 2 | **1.31 ± 0.20** |
| Mm.331051 | Gmps | Guanine monophosphate synthetase | **1.31 ± 0.11** |
| Mm.21482 | Clns1a | Chloride channel, nucleotide-sensitive, 1A | **1.30 ± 0.06** |
| Mm.163 | B2m | Beta-2 microglobulin | **1.30 ± 0.20** |
| Mm.247428 | AF067061 | CDNA sequence AF067061 | **1.29 ± 0.22** |
| Mm.13944 | Rps9 | Ribosomal protein S9 | **1.29 ± 0.20** |
| Mm.136093 | Atp5k | ATP synthase, H+ transporting, mitochondrial F1F0 complex, subunit e | **1.28 ± 0.14** |
| Mm.34779 | Uxt | Ubiquitously expressed transcript | **1.28 ± 0.26** |
| Mm.322294 | Ndufb9 | NADH dehydrogenase (ubiquinone) 1 beta subcomplex, 9 | **1.28 ± 0.29** |
| Mm.165735 | Lsm2 | LSM2 homolog, U6 small nuclear RNA associated | **1.27 ± 0.04** |
| Mm.480556 | Pgam1 | Phosphoglycerate mutase 1 | **1.27 ± 0.15** |
| Mm.24108 | Mrps21 | Mitochondrial ribosomal protein S21 | **1.27 ± 0.24** |
| Mm.4595 | Fbl | Fibrillarin | **1.27 ± 0.12** |
| Mm.3624 | Guk1 | Guanylate kinase 1 | **1.25 ± 0.17** |
| Mm.30113 | Ndufs3 | NADH dehydrogenase (ubiquinone) Fe-S protein 3 | **1.24 ± 0.18** |
| Mm.195628 | Mrps15 | Mitochondrial ribosomal protein S15 | **1.23 ± 0.25** |
| Mm.391959 | 1500012F01Rik | RIKEN cDNA 1500012F01 gene | **1.22 ± 0.03** |
| Mm.21932 | 2700060E02Rik | RIKEN cDNA 2700060E02 gene | **1.22 ± 0.04** |
| Mm.334332 | Muc5ac | Mucin 5, subtypes A and C, tracheobronchial/gastric | **1.21 ± 0.16** |
| Mm.262345 | Pcolce | Procollagen C-endopeptidase enhancer protein | **1.21 ± 0.24** |
| Mm.3979 | Ubl4 | Ubiquitin-like 4 | **1.19 ± 0.25** |
| Mm.276041 | 3110003A17Rik | RIKEN cDNA 3110003A17 gene | **1.18 ± 0.17** |
| Mm.22776 | Eif3c | Eukaryotic translation initiation factor 3, subunit C | **1.17 ± 0.09** |
| Mm.377477 | Olfr1325 | Olfactory receptor 1325 | **1.17 ± 0.18** |
| Mm.371688 | Btf3 | Basic transcription factor 3 | **1.17 ± 0.20** |
| Mm.377799 | Olfr705 | Olfactory receptor 705 | **1.17 ± 0.15** |
| Mm.30181 | Pdzk1ip1 | PDZK1 interacting protein 1 | **1.17 ± 0.22** |
| Mm.390983 | Psmb9 | Proteasome (prosome, macropain) subunit, beta type 9 | **1.16 ± 0.26** |
| Mm.249932 | A230050P20Rik | RIKEN cDNA A230050P20 gene | **1.16 ± 0.13** |
| Mm.7281 | Col5a1 | Collagen, type V, alpha 1 | **1.16 ± 0.13** |
| Mm.262151 | Fam54a | Family with sequence similarity 54, member A | **1.16 ± 0.19** |
| Mm.28541 | Pgp | Phosphoglycolate phosphatase | **1.15 ± 0.12** |
| Mm.210447 | Ehbp1l1 | EH domain binding protein 1-like 1 | **1.13 ± 0.22** |
| Mm.2930 | Ppan | Peter pan homolog | **1.13 ± 0.14** |
| Mm.715 | Hck | Hemopoietic cell kinase | **1.10 ± 0.13** |
| Mm.28890 | Naaa | N-acylethanolamine acid amidase | **1.09 ± 0.07** |
| Mm.41868 | 2810405K02Rik | RIKEN cDNA 2810405K02 gene | **1.08 ± 0.21** |
| Mm.276293 | Mrpl19 | Mitochondrial ribosomal protein L19 | **1.08 ± 0.08** |
| Mm.256035 | Hax1 | HCLS1 associated X-1 | **1.08 ± 0.24** |
| Mm.427321 | Hnrnpc | Heterogeneous nuclear ribonucleoprotein C | **1.08 ± 0.19** |
| Mm.384762 | Hjurp | Holliday junction recognition protein | **1.07 ± 0.18** |
| Mm.98 | Psmb6 | Proteasome (prosome, macropain) subunit, beta type 6 | **1.07 ± 0.19** |
| Mm.28922 | Farsb | Phenylalanyl-tRNA synthetase, beta subunit | **1.07 ± 0.08** |
| Mm.1685 | Plxna3 | Plexin A3 | **1.06 ± 0.15** |
| Mm.297297 | Eddm3b | Epididymal protein 3B | **1.06 ± 0.18** |
| Mm.335611 | Gsdmc2 | Gasdermin C2 | **1.06 ± 0.24** |
| Mm.26614 | Mrpl30 | Mitochondrial ribosomal protein L30 | **1.05 ± 0.22** |
| Mm.200231 | Hscb | Hscb iron-sulfur cluster co-chaperone homolog | **1.05 ± 0.13** |
| Mm.1104 | Uba1 | Ubiquitin-like modifier activating enzyme 1 | **1.05 ± 0.09** |
| Mm.288697 | Slc25a27 | Solute carrier family 25, member 27 | **1.04 ± 0.16** |
| Mm.280013 | Psmd1 | Proteasome (prosome, macropain) 26S subunit, non-ATPase, 1 | **1.03 ± 0.18** |
| Mm.23896 | Slc25a46 | Solute carrier family 25, member 46 | **1.03 ± 0.17** |
| Mm.178818 | Tcf25 | Transcription factor 25 (basic helix-loop-helix) | **1.02 ± 0.09** |
| Mm.475640 | Cpsf3l | Cleavage and polyadenylation specific factor 3-like | **1.02 ± 0.19** |
| Mm.235814 | Rdh9 | Retinol dehydrogenase 9 | **1.02 ± 0.14** |
| Mm.258927 | Eef1d | Eukaryotic translation elongation factor 1 delta (guanine nucleotide exchange protein) | **1.01 ± 0.11** |
| Mm.28173 | Mars | Methionine-tRNA synthetase | **1.01 ± 0.16** |
| Mm.267692 | Glrx3 | Glutaredoxin 3 | **1.01 ± 0.21** |
| Mm.42944 | Skp1a | S-phase kinase-associated protein 1A | **1.00 ± 0.19** |
| Mm.271486 | Olfr1163 | Olfactory receptor 1163 | **1.00 ± 0.20** |
| Mm.196173 | Gm12715 | PREDICTED: predicted gene 12715 | **-1.01 ± 0.03** |
| Mm.285300 | A930018M24Rik | RIKEN cDNA A930018M24 gene | **-1.04 ± 0.23** |
| Mm.444029 | Plxna4 | Plexin A4 | **-1.05 ± 0.16** |
| Mm.387073 | Unc79 | Unc-79 homolog | **-1.18 ± 0.14** |
| Mm.27886 | Ndufa12 | NADH dehydrogenase (ubiquinone) 1 alpha subcomplex, 12 | **-1.22 ± 0.14** |
| Mm.2390 | Cited1 | Cbp/p300-interacting transactivator with Glu/Asp-rich carboxy-terminal domain 1 | **-1.24 ± 0.21** |
| Mm.255398 | Nup153 | Nucleoporin 153 | **-1.37 ± 0.26** |
| Mm.391473 | Emx1 | Empty spiracles homolog 1 | **-1.44 ± 0.16** |
| Mm.4610 | Upp1 | Uridine phosphorylase 1 | **-1.46 ± 0.09** |
| Mm.275320 | Eln | Elastin | **-1.48 ± 0.07** |
| Mm.152987 | Xpo7 | Exportin 7 | **-1.84 ± 0.05** |
| Mm.110594 | Rgl3 | Ral guanine nucleotide dissociation stimulator-like 3 | **-1.85 ± 0.08** |
| Mm.485787 | Rpl5 | Ribosomal protein L5 | **-1.87 ± 0.21** |
| Mm.29945 | Edf1 | Endothelial differentiation-related factor 1 | **-2.06 ± 0.45** |
| Mm.358682 | BC051019 | CDNA sequence BC051019 | **-2.08 ± 0.26** |
| Mm.23596 | Cenpm | Centromere protein M | **-2.57 ± 0.58** |
| Mm.212921 | 4930432E11Rik | PREDICTED: RIKEN cDNA 4930432E11 gene | **-2.65 ± 0.34** |
