# Supplementary material for: Differential host gene responses from infection with neurovirulent and partially-neurovirulent strains of Venezuelan equine encephalitis virus
Source: BMC Infect Dis. 2017 Apr 26;17:309. doi: 10.1186/s12879-017-2355-3 (PMC5405508; doi:10.1186/s12879-017-2355-3)
Supplement: Supplementary file 7 — Significantly modulated genes in the brain that were unique to V3034 infection. Genes that were modulated only with V3034 infection in the brain were identified. The list summarizes the commonly modulated genes for each time point studied. Values are expressed as average values of (log2) fold expression for each gene over uninfected controls ± standard error mean (SEM). * P ≤ 0.05. (DOCX 29 kb) [file 12879_2017_2355_MOESM7_ESM.docx]

**Additional file 7: Table S6: Significantly modulated genes in brain those were unique to V3034 infection**

| **UniGene** | **Gene** | **Description** | **Log_2_ Exp ± SEM** |
| --- | --- | --- | --- |
| **Genes uniquely modulated at 48 h pi** | | | |
| Mm.425949 | Ly6a | Lymphocyte antigen 6 complex, locus A | **1.40 ± 0.27** |
| Mm.259105 | Odf2l | Outer dense fiber of sperm tails 2-like | **1.32 ± 0.20** |
| Mm.272746 | Cmtm2a | CKLF-like MARVEL transmembrane domain containing 2A | **1.30 ± 0.17** |
| Mm.377828 | Olfr547 | Olfactory receptor 547 | **1.26 ± 0.09** |
| Mm.30466 | Trps1 | Trichorhinophalangeal syndrome I | **1.21 ± 0.04** |
| Mm.281079 | Wdr12 | WD repeat domain 12 | **1.18 ± 0.14** |
| Mm.389688 | Oas1g | 2'-5' oligoadenylate synthetase 1G | **1.18 ± 0.27** |
| Mm.270511 | Tcerg1 | Transcription elongation regulator 1 (CA150) | **1.12 ± 0.12** |
| Mm.377402 | Olfr1511 | Olfactory receptor 1511 | **1.10 ± 0.15** |
| Mm.431282 | H2-Q6 | Histocompatibility 2, Q region locus 6 | **1.08 ± 0.08** |
| Mm.234441 | Lmcd1 | LIM and cysteine-rich domains 1 | **1.07 ± 0.12** |
| Mm.357108 | Tmem178 | Transmembrane protein 178 | **1.06 ± 0.15** |
| Mm.34428 | Pias4 | Protein inhibitor of activated STAT 4 | **1.04 ± 0.10** |
| Mm.259916 | Chdh | Choline dehydrogenase | **1.02 ± 0.22** |
| Mm.1114 | Gla | Galactosidase, alpha | **1.00 ± 0.20** |
| Mm.302516 | Fsip2 | PREDICTED: fibrous sheath-interacting protein 2 | **1.00 ± 0.16** |
| Mm.423664 | Gm2058 | Predicted gene 2058 | **-1.01 ± 0.05** |
| Mm.57734 | Lims1 | LIM and senescent cell antigen-like domains 1 | **-1.02 ± 0.11** |
| Mm.37426 | Cd163 | CD163 antigen | **-1.02 ± 0.12** |
| Mm.485067 | 4930481A15Rik | RIKEN cDNA 4930481A15 gene | **-1.03 ± 0.23** |
| Mm.209774 | Ccdc91 | Coiled-coil domain containing 91 | **-1.04 ± 0.24** |
| Mm.54120 | Samd4b | Sterile alpha motif domain containing 4B | **-1.05 ± 0.17** |
| Mm.2556 | Azi1 | 5-azacytidine induced gene 1 | **-1.07 ± 0.17** |
| Mm.387073 | Unc79 | Unc-79 homolog | **-1.10 ± 0.08** |
| Mm.327442 | Ldb1 | LIM domain binding 1 | **-1.11 ± 0.09** |
| Mm.332844 | Cyp3a11 | Cytochrome P450, family 3, subfamily a, polypeptide 11 | **-1.12 ± 0.12** |
| Mm.291214 | Meaf6 | MYST/Esa1-associated factor 6 | **-1.13 ± 0.22** |
| Mm.25138 | Tmigd1 | Transmembrane and immunoglobulin domain containing 1 | **-1.14 ± 0.09** |
| Mm.244068 | Arhgef7 | Rho guanine nucleotide exchange factor (GEF7) | **-1.19 ± 0.05** |
| Mm.191949 | Camk1d | Calcium/calmodulin-dependent protein kinase ID | **-1.23 ± 0.14** |
| Mm.289796 | Lins | Lines homolog | **-1.26 ± 0.09** |
| Mm.374850 | Dclre1b | DNA cross-link repair 1B, PSO2 homolog | **-1.26 ± 0.19** |
| Mm.373672 | Ttn | Titin | **-1.26 ± 0.10** |
| Mm.483321 | Bmp10 | Bone morphogenetic protein 10 | **-1.28 ± 0.29** |
| Mm.246625 | Kir3dl1 | Killer cell immunoglobulin-like receptor, three domains, long cytoplasmic tail, 1 | **-1.34 ± 0.29** |
| Mm.443529 | Arhgap20 | Rho GTPase activating protein 20 | **-1.37 ± 0.23** |
| Mm.439929 | 2810001A02Rik | PREDICTED: RIKEN cDNA 2810001A02 gene | **-1.42 ± 0.23** |
| Mm.23596 | Cenpm | Centromere protein M | **-1.48 ± 0.32** |
| Mm.333851 | Vpreb3 | Pre-B lymphocyte gene 3 | **-1.55 ± 0.19** |
| Mm.44763 | 1300018J18Rik | RIKEN cDNA 1300018J18 gene | **-1.62 ± 0.14** |
| Mm.258300 | Agpat4 | 1-acylglycerol-3-phosphate O-acyltransferase 4 | **-1.66 ± 0.34** |
| Mm.377086 | Hoxa13 | Homeobox A13 | **-1.72 ± 0.24** |
| Mm.271724 | Dtx3 | Deltex 3 homolog | **-1.84 ± 0.24** |
| Mm.443660 | Lrrc4 | Leucine rich repeat containing 4 | **-2.19 ± 0.50** |
| Mm.28839 | Eif2b1 | Eukaryotic translation initiation factor 2B, subunit 1 (alpha) | **-2.32 ± 0.49** |
| **Genes uniquely modulated at 72 h pi** | | | |
| Mm.28162 | Nup210 | Nucleoporin 210 | **1.90 ± 0.09** |
| Mm.338001 | Pld5 | Phospholipase D family, member 5 | **1.86 ± 0.19** |
| Mm.211477 | Phldb2 | Pleckstrin homology-like domain, family B, member 2 | **1.83 ± 0.32** |
| Mm.22948 | Ubtd1 | Ubiquitin domain containing 1 | **1.77 ± 0.25** |
| Mm.269029 | Slc7a6os | Solute carrier family 7, member 6 opposite strand | **1.63 ± 0.35** |
| Mm.484214 | LOC100503803 | PREDICTED: uncharacterized protein c2orf16-like | **1.62 ± 0.28** |
| Mm.41385 | Isoc2b | Isochorismatase domain containing 2b | **1.51 ± 0.23** |
| Mm.272746 | Cmtm2a | CKLF-like MARVEL transmembrane domain containing 2A | **1.34 ± 0.12** |
| Mm.207062 | Hoxc13 | Homeobox C13 | **1.31 ± 0.16** |
| Mm.90450 | Myo1a | Myosin IA | **1.29 ± 0.26** |
| Mm.290669 | Ndfip2 | Nedd4 family interacting protein 2 | **1.29 ± 0.11** |
| Mm.358668 | Prpf40b | PRP40 pre-mRNA processing factor 40 homolog B | **1.29 ± 0.19** |
| Mm.442861 | H2-D4 | Histocompatibility 2, D region locus 4 | **1.25 ± 0.22** |
| Mm.2121 | Ifi27l1 | Interferon, alpha-inducible protein 27 like 1 | **1.22 ± 0.15** |
| Mm.159684 | Tmpo | Thymopoietin | **1.21 ± 0.22** |
| Mm.482110 | Ly6c2 | Lymphocyte antigen 6 complex, locus C2 | **1.21 ± 0.22** |
| Mm.281079 | Wdr12 | WD repeat domain 12 | **1.16 ± 0.17** |
| Mm.27925 | Dbndd1 | Dysbindin (dystrobrevin binding protein 1) domain containing 1 | **1.11 ± 0.12** |
| Mm.220224 | Gria2 | Glutamate receptor, ionotropic, AMPA2 (alpha 2) | **1.11 ± 0.05** |
| Mm.30466 | Trps1 | Trichorhinophalangeal syndrome I | **1.10 ± 0.10** |
| Mm.271745 | Nrp1 | Neuropilin 1 | **1.09 ± 0.05** |
| Mm.482724 | Gm13102 | Predicted gene 13102 | **1.08 ± 0.22** |
| Mm.109380 | 4930583I09Rik | PREDICTED: RIKEN cDNA 4930583I09 gene | **1.08 ± 0.22** |
| Mm.42150 | Rasgrp1 | RAS guanyl releasing protein 1 | **1.05 ± 0.20** |
| Mm.9901 | Nucb2 | Nucleobindin 2 | **1.00 ± 0.16** |
| Mm.302516 | Fsip2 | PREDICTED: fibrous sheath-interacting protein 2 | **1.00 ± 0.15** |
| Mm.377863 | Olfr623 | Olfactory receptor 623 ( | **1.00 ± 0.14** |
| Mm.27503 | 1810009A15Rik | RIKEN cDNA 1810009A15 gene | **-1.01 ± 0.19** |
| Mm.29586 | Basp1 | Brain abundant, membrane attached signal protein 1 | **-1.02 ± 0.22** |
| Mm.31415 | 1700110M21Rik | RIKEN cDNA 1700110M21 gene | **-1.06 ± 0.19** |
| Mm.340163 | Myh8 | Myosin, heavy polypeptide 8, skeletal muscle, perinatal | **-1.09 ± 0.14** |
| Mm.34608 | Clybl | Citrate lyase beta like | **-1.11 ± 0.13** |
| Mm.483321 | Bmp10 | Bone morphogenetic protein 10 | **-1.13 ± 0.07** |
| Mm.208465 | Ccdc53 | Coiled-coil domain containing 53 | **-1.15 ± 0.16** |
| Mm.102080 | Gdap1l1 | Ganglioside-induced differentiation-associated protein 1-like 1 | **-1.15 ± 0.26** |
| Mm.240252 | Trim14 | Tripartite motif-containing 14 | **-1.16 ± 0.06** |
| Mm.6898 | Naip1 | NLR family, apoptosis inhibitory protein 1 | **-1.18 ± 0.13** |
| Mm.37426 | Cd163 | CD163 antigen | **-1.19 ± 0.25** |
| Mm.240830 | Dab2 | Disabled homolog 2 | **-1.19 ± 0.06** |
| Mm.202665 | Rnase4 | Ribonuclease, Rnase A family 4 | **-1.20 ± 0.04** |
| Mm.24214 | Foxk1 | Forkhead box K1 | **-1.26 ± 0.19** |
| Mm.120151 | Fam168b | Family with sequence similarity 168, member B | **-1.28 ± 0.19** |
| Mm.261984 | Glo1 | Glyoxalase 1 | **-1.28 ± 0.06** |
| Mm.391424 | Ppfia2 | Protein tyrosine phosphatase, receptor type, f polypeptide (PTPRF), interacting protein (liprin), alpha 2 | **-1.30 ± 0.14** |
| Mm.482922 | 1700055N04Rik | PREDICTED: RIKEN cDNA 1700055N04 gene, transcript variant 1 | **-1.34 ± 0.17** |
| Mm.191949 | Camk1d | Calcium/calmodulin-dependent protein kinase ID | **-1.36 ± 0.19** |
| Mm.439929 | 2810001A02Rik | PREDICTED: RIKEN cDNA 2810001A02 gene | **-1.36 ± 0.12** |
| Mm.292729 | Traf6 | TNF receptor-associated factor 6 | **-1.37 ± 0.28** |
| Mm.103439 | Gprc5b | G protein-coupled receptor, family C, group 5, member B | **-1.38 ± 0.17** |
| Mm.285685 | Cd164l2 | CD164 sialomucin-like 2 | **-1.42 ± 0.29** |
| Mm.374850 | Dclre1b | DNA cross-link repair 1B, PSO2 homolog | **-1.43 ± 0.18** |
| Mm.46561 | Lect1 | Leukocyte cell derived chemotaxin 1 | **-1.50 ± 0.25** |
| Mm.290868 | Tom1 | Target of myb1 homolog | **-1.51 ± 0.07** |
| Mm.213651 | Gk5 | Glycerol kinase 5 | **-1.52 ± 0.19** |
| Mm.235346 | Eps8 | Epidermal growth factor receptor pathway substrate 8 | **-1.55 ± 0.23** |
| Mm.368256 | Gm9758 | Predicted gene 9758 | **-1.60 ± 0.14** |
| Mm.55952 | Adam26b | A disintegrin and metallopeptidase domain 26B | **-1.75 ± 0.09** |
| Mm.30204 | Qdpr | Quinoid dihydropteridine reductase | **-1.75 ± 0.12** |
| Mm.252987 | Slc12a5 | Solute carrier family 12, member 5 | **-1.80 ± 0.25** |
| Mm.334775 | Cenpt | Centromere protein T | **-1.85 ± 0.05** |
| Mm.333851 | Vpreb3 | Pre-B lymphocyte gene 3 | **-1.85 ± 0.31** |
| Mm.196158 | Kcnk10 | Potassium channel, subfamily K, member 10 | **-1.85 ± 0.42** |
| Mm.213406 | Uggt2 | UDP-glucose glycoprotein glucosyltransferase 2 | **-1.89 ± 0.43** |
| Mm.449996 | 4921506M07Rik | RIKEN cDNA 4921506M07 gene | **-1.95 ± 0.10** |
| Mm.107441 | Zfp26 | Zinc finger protein 26 | **-2.07 ± 0.16** |
| Mm.153039 | Mpdz | Multiple PDZ domain protein | **-2.19 ± 0.19** |
| Mm.194536 | Gmfg | Glia maturation factor, gamma | **-2.25 ± 0.36** |
| Mm.34330 | Nnat | Neuronatin | **-2.26 ± 0.19** |
| Mm.6442 | Pkd2 | Polycystic kidney disease 2 | **-2.46 ± 0.51** |
| Mm.23596 | Cenpm | Centromere protein M | **-2.48 ± 0.55** |
| Mm.288567 | Beta-s | Hemoglobin subunit beta-1-like | **-2.62 ± 0.33** |
| Mm.30533 | Gapt | Grb2-binding adaptor, transmembrane | **-2.88 ± 0.53** |
| Mm.479929 | Zufsp | Zinc finger with UFM1-specific peptidase domain | **-2.95 ± 0.49** |
| Mm.19987 | Dct | Dopachrome tautomerase | **-2.95 ± 0.68** |
| Mm.234204 | Pak2 | P21 protein (Cdc42/Rac)-activated kinase 2 | **-3.16 ± 0.51** |
| Mm.475174 | Far2 | Fatty acyl coA reductase 2 | **-3.27 ± 0.62** |
| Mm.12834 | Lfng | LFNG O-fucosylpeptide 3-beta-N-acetylglucosaminyltransferase | **-3.53 ± 0.38** |
| Mm.196110 | Hba-a2 | Hemoglobin alpha, adult chain 2 | **-3.95 ± 0.43** |
| Mm.415 | Iapp | Islet amyloid polypeptide | **-4.85 ± 1.04** |
| **Genes uniquely modulated at 96 h pi** | | | |
| Mm.220224 | Gria2 | Glutamate receptor, ionotropic, AMPA2 (alpha 2) | **1.28 ± 0.12** |
| Mm.482431 | Tesk2 | Testis-specific kinase 2 | **1.22 ± 0.22** |
| Mm.159684 | Tmpo | Thymopoietin | **1.13 ± 0.10** |
| Mm.482724 | Gm13102 | Predicted gene 13102 | **1.12 ± 0.04** |
| Mm.28536 | Dpy30 | Dpy-30 homolog | **1.06 ± 0.10** |
| Mm.258 | Atp5g1 | ATP synthase, H+ transporting, mitochondrial F0 complex, subunit c1 (subunit 9) | **1.05 ± 0.08** |
| Mm.158971 | 1110001J03Rik | RIKEN cDNA 1110001J03 gene | **1.05 ± 0.09** |
| Mm.380129 | G3bp1 | Ras-GTPase-activating protein SH3-domain binding protein 1 | **1.02 ± 0.11** |
| Mm.333386 | Lpar5 | Lysophosphatidic acid receptor 5 | **1.02 ± 0.11** |
| Mm.482114 | Rab4b | RAB4B, member RAS oncogene family | **1.01 ± 0.04** |
| Mm.8739 | Sgce | Sarcoglycan, epsilon | **1.00 ± 0.04** |
| Mm.279780 | Rab17 | RAB17, member RAS oncogene family | **1.00 ± 0.14** |
| Mm.24125 | Col4a3bp | Collagen, type IV, alpha 3 (Goodpasture antigen) binding protein | **-1.00 ± 0.18** |
| Mm.55847 | Spag17 | Sperm associated antigen 17 | **-1.01 ± 0.19** |
| Mm.202665 | Rnase4 | Ribonuclease, Rnase A family 4 | **-1.01 ± 0.19** |
| Mm.483321 | Bmp10 | Bone morphogenetic protein 10 | **-1.01 ± 0.13** |
| Mm.336054 | Gm595 | Predicted gene 595 | **-1.03 ± 0.02** |
| Mm.331893 | Hmgb4 | High-mobility group box 4 | **-1.03 ± 0.14** |
| Mm.29586 | Basp1 | Brain abundant, membrane attached signal protein 1 | **-1.05 ± 0.21** |
| Mm.327442 | Ldb1 | LIM domain binding 1 | **-1.06 ± 0.22** |
| Mm.485856 | Zfp87 | Zinc finger protein 87 | **-1.07 ± 0.07** |
| Mm.119274 | Prrxl1 | Paired related homeobox protein-like 1 | **-1.11 ± 0.15** |
| Mm.484620 | Gm340 | PREDICTED: predicted gene 340 | **-1.11 ± 0.11** |
| Mm.390829 | Mtbp | Mdm2, transformed 3T3 cell double minute p53 binding protein | **-1.12 ± 0.16** |
| Mm.26908 | Csnk1a1 | Casein kinase 1, alpha 1 | **-1.12 ± 0.04** |
| Mm.269088 | Anp32a | Acidic (leucine-rich) nuclear phosphoprotein 32 family, member A | **-1.16 ± 0.18** |
| Mm.37426 | Cd163 | CD163 antigen | **-1.17 ± 0.06** |
| Mm.250428 | Cypt2-ps | Cysteine-rich perinuclear theca 2, pseudogene | **-1.19 ± 0.20** |
| Mm.235346 | Eps8 | Epidermal growth factor receptor pathway substrate 8 | **-1.21 ± 0.04** |
| Mm.223420 | Olfr711 | Olfactory receptor 711 | **-1.21 ± 0.18** |
| Mm.35650 | Tspan31 | Tetraspanin 31 | **-1.27 ± 0.14** |
| Mm.292729 | Traf6 | TNF receptor-associated factor 6 | **-1.32 ± 0.06** |
| Mm.387073 | Unc79 | Unc-79 homolog | **-1.38 ± 0.15** |
| Mm.13787 | Cp | Ceruloplasmin | **-1.50 ± 0.11** |
| Mm.308500 | Pcdha2 | Protocadherin alpha 2 | **-1.51 ± 0.23** |
| Mm.271711 | Tagln2 | Transgelin 2 | **-1.57 ± 0.24** |
| Mm.12715 | Gpx8 | Glutathione peroxidase 8 | **-1.72 ± 0.03** |
| Mm.194536 | Gmfg | Glia maturation factor, gamma | **-1.91 ± 0.24** |
| Mm.368256 | Gm9758 | Predicted gene 9758 | **-2.12 ± 0.12** |
| Mm.131237 | Stap1 | Signal transducing adaptor family member 1 | **-2.13 ± 0.31** |
| Mm.390589 | Omd | Osteomodulin | **-2.17 ± 0.22** |
