# Supplementary material for: Differential host gene responses from infection with neurovirulent and partially-neurovirulent strains of Venezuelan equine encephalitis virus
Source: BMC Infect Dis. 2017 Apr 26;17:309. doi: 10.1186/s12879-017-2355-3 (PMC5405508; doi:10.1186/s12879-017-2355-3)
Supplement: Supplementary file 8 — Primer sequences used for real-time PCR. Real-time PCR analysis was performed to confirm the microarray results for Stat1, Stat2, Zfp456, Nt5c2, NfκB2 and Samd9l post V3000 and V3034 infections at various time points. Expression values of all the genes were normalized with the house keeping gene, GAPDH. The details of primer sets used are summarized in this table. (DOCX 14 kb) [file 12879_2017_2355_MOESM8_ESM.docx]

**Additional file 8: Table S7- Primer sequences used for quantitative RT-PCR**

| **Gene** | **Unigene ID** | **Forward Primer (5`-3`)** | **Reverse Primer (5`-3`)** |
| --- | --- | --- | --- |
| **Nt5c2** | Mm.40965 | accgcacgtcagtggatttcaa | tcatggcagtgtgtgatctcct |
| **Samd9l** | Mm.196013 | tgttggtgtgcaagtcacca | acaagccctggcttcactgatt |
| **Zfp456** | Mm.461583 | ttgctgtctgctggctaccttt | Tctactccctgtgttcactgct |
| **NFκB2** | Mm.102365 | tggaacagcccaaacagc | cacctggcaaacctccat |
| **Stat1** | Mm.277406 | aaattcacctatgagcccgaccct | aatgagctgctggaagaggaggaa |
| **Stat2** | Mm.293120 | ggaacagctggaacagtggt | gtagctgccgaaggtgga |
| **GAPDH** | Mm.304088 | gggtgaggccggtgctgagt | cacccttcaagtgggccccg |
